# Liver Metastases and Immune Checkpoint Inhibitor Efficacy in Patients With Refractory Metastatic Colorectal Cancer: A Secondary Analysis of a Randomized Clinical Trial

**DOI:** 10.1001/jamanetworkopen.2023.46094

**Published:** 2023-12-05

**Authors:** Eric X. Chen, Jonathan M. Loree, Emma Titmuss, Derek J. Jonker, Hagen F. Kennecke, Scott Berry, Felix Couture, Chaudharry E. Ahmad, John R. Goffin, Petr Kavan, Mohammed Harb, Bruce Colwell, Setareh Samimi, Benoit Samson, Tahir Abbas, Nathalie Aucoin, Francine Aubin, Sheryl Koski, Alice C. Wei, Dongsheng Tu, Chris J. O’Callaghan

**Affiliations:** 1 Princess Margaret Cancer Center, Toronto, Ontario, Canada; 2 British Columbia Cancer Agency, Vancouver, Canada; 3 The Ottawa Hospital Research Institute, University of Ottawa, Ottawa, Ontario, Canada; 4 Portland Providence Cancer Center, Earle Chiles Research Institute, Portland, Oregon; 5 Department of Oncology, Queen’s University, Kingston, Ontario, Canada; 6 L’Hôtel-Dieu de Québec, Laval, Quebec, Canada; 7 Eastern Health, St John’s, Newfoundland and Labrador, Canada; 8 Juravinski Cancer Center, Hamilton, Ontario, Canada; 9 Segal Cancer Center, Montreal, Quebec, Canada; 10 Moncton Hospital, Moncton, Brunswick, Canada; 11 Queen Elizabeth II Health Sciences Center, Halifax, Nova Scotia, Canada; 12 Hôpital Sacré-Coeur de Montréal, Montreal, Quebec, Canada; 13 Charles LeMoyne Hospital Cancer Centre, Sherbrooke, Quebec, Canada; 14 Saskatoon Cancer Center, Saskatoon, Saskatoon, Canada; 15 Hôpital de la Cité-de-la-Santé, Laval, Quebec, Canada; 16 Centre Hospitalier de l’Université de Montréal, Montreal, Quebec, Canada; 17 Cross Cancer Center, Edmonton, Alberta, Canada; 18 Memorial Sloan Kettering Cancer Center, New York, New York; 19 Canadian Cancer Trials Group, Kingston, Ontario, Canada

## Abstract

**Importance:**

Immune checkpoint inhibitors (ICIs) have limited activity in microsatellite-stable (MSS) or mismatch repair–proficient (pMMR) colorectal cancer. Recent findings suggest the efficacy of ICIs may be modulated by the presence of liver metastases (LM).

**Objective:**

To investigate the association between the presence of LM and ICI activity in advanced MSS colorectal cancer.

**Design, Setting, and Participants:**

In this secondary analysis of the Canadian Cancer Trials Group CO26 (CCTG CO.26) randomized clinical trial, patients with treatment-refractory colorectal cancer were randomized in a 2:1 fashion to durvalumab plus tremelimumab or best supportive care alone between August 10, 2016, and June 15, 2017. The primary end point was overall survival (OS) with 80% power and 2-sided α = .10. The median follow-up was 15.2 (0.2-22.0) months. In this post hoc analysis performed from February 11 to 14, 2022, subgroups were defined based on the presence or absence of LM and study treatments.

**Intervention:**

Durvalumab plus tremelimumab or best supportive care.

**Main Outcomes and Measures:**

Hazard ratios (HRs) and 90% CIs were calculated based on a stratified Cox proportional hazards regression model. Plasma tumor mutation burden at study entry was determined using a circulating tumor DNA assay. The primary end point of the study was OS, defined as the time from randomization to death due to any cause; secondary end points included progression-free survival (PFS) and disease control rate (DCR).

**Results:**

Of 180 patients enrolled (median age, 65 [IQR, 36-87] years; 121 [67.2%] men; 19 [10.6%] Asian, 151 [83.9%] White, and 10 [5.6%] other race or ethnicity), LM were present in 127 (70.6%). For patients with LM, there was a higher proportion of male patients (94 of 127 [74.0%] vs 27 of 53 [50.9%]; *P* = .005), and the time from initial cancer diagnosis to study entry was shorter (median, 40 [range, 8-153] vs 56 [range, 14-181] months; *P* = .001). Plasma tumor mutation burden was significantly higher in patients with LM. Patients without LM had significantly improved PFS with durvalumab plus tremelimumab (HR, 0.54 [90% CI, 0.35-0.96]; *P* = .08; *P* = .02 for interaction). Disease control rate was 49% (90% CI, 36%-62%) in patients without LM treated with durvalumab plus tremelimumab, compared with 14% (90% CI, 6%-38%) in those with LM (odds ratio, 5.70 [90% CI, 1.46-22.25]; *P* = .03). On multivariable analysis, patients without LM had significantly improved OS and PFS compared with patients with LM.

**Conclusions and Relevance:**

In this secondary analysis of the CCTG CO.26 study, the presence of LM was associated with worse outcomes for patients with advanced colorectal cancer. Patients without LM had improved PFS and higher DCR with durvalumab plus tremelimumab. Liver metastases may be associated with poor outcomes of ICI treatment in advanced colorectal cancer and should be considered in the design and interpretation of future clinical studies evaluating this therapy.

## Key Points

**Question:**

Is microsatellite-stable or mismatch repair–proficient advanced colorectal cancer with liver metastases (LM) resistant to immune checkpoint inhibitors?

**Findings:**

In this secondary analysis of a randomized clinical trial of 180 patients, those without LM had significantly improved progression-free and overall survival. In patients without LM, durvalumab and tremelimumab treatment was associated with improved progression-free survival and disease control rate, not overall survival.

**Meaning:**

Future clinical trials of immune checkpoint inhibitors in microsatellite-stable or mismatch repair–proficient advanced colorectal cancer should stratify patients according to the presence of LM and focus on understanding the mechanism of resistance by LM.

## Teaser

This secondary analysis of a randomized clinical trial investigates whether the presence of liver metastasis is an indicator of treatment resistance to immune checkpoint inhibitors (ICIs) in patients with advanced colorectal cancer.

## Introduction

Colorectal cancer is the third most commonly diagnosed cancer and the second leading cause of cancer-related mortality worldwide. In 2020, approximately 1.93 million patients were diagnosed with colorectal cancer, resulting in 935 000 deaths.^1^ Although immune checkpoint inhibitors (ICIs) have revolutionized the treatment of many types of cancers, their benefits are restricted to patients with microsatellite instability–high (MSI-H) or mismatch repair–deficient (dMMR) colorectal cancers, representing approximately 5% of patients with advanced colorectal cancer.^2,3,4^ For most patients with microsatellite-stable (MSS) or mismatch repair–proficient (pMMR) colorectal cancer, ICIs, either alone or in combination with other agents, have not been effective.^5,6^

Recent findings suggest that advanced colorectal cancer with metastatic disease in the liver may be resistant to ICI treatment. For example, Fakih et al^7^ showed that the overall response rate was 21.7% in patients without liver metastasis (LM) vs 0% in those with LM in a single-arm, phase 2 study of nivolumab and regorafenib in advanced colorectal cancer. The present study was conducted to investigate whether the presence of LM is an indicator of treatment resistance to ICIs in advanced colorectal cancer.

## Methods

The Canadian Cancer Trials Group (CCTG) CO.26 study is a phase 2 study that randomized 180 patients with treatment-refractory advanced colorectal cancer unselected for MSI status to durvalumab, an antibody against programmed death ligand 1, plus tremelimumab, an antibody against the cytotoxic T-cell lymphocyte antigen-4 or best supportive care (BSC) in a 2:1 fashion between August 10, 2016, and June 15, 2017.^5^ The primary end point of the study was overall survival (OS), defined as the time from randomization to death due to any cause. The secondary end points included progression-free survival (PFS) and disease control rate (DCR). The study was designed to have a power of 80% and a 2-sided α of 10% to detect a 35% reduction in the continuous risk of death. The study was approved by the institutional review board of each participating center. Patients provided written informed consent prior to study participation. Self-reported race and ethnicity, collected to ensure a diverse group of patients were enrolled, were categorized as Asian, White, and other race or ethnicity (including American Indian or Alaska Native, Black, Native Hawaiian or Other Pacific Islander, and unknown race or ethnicity). The median follow-up was 15.2 (0.2-22.0) months. The trial protocol is provided in Supplement 1. This study followed the Consolidated Standards of Reporting Trials (CONSORT) reporting guideline.

### Statistical Analysis

For this retrospective secondary analysis performed from February 11 to 14, 2022, patients were divided into groups based on the presence or absence of LM and study treatments. Cohorts with and without LM were based on radiological findings at the time of study entry. Plasma tumor mutation burden (pTMB) was determined from blood samples collected prior to study therapy using a circulating tumor DNA assay (GuardantOMNI next-generation sequencing panel; Guardant Health Inc) and reported as mutations per megabase (Mb). Overall survival and PFS were analyzed according to intention to treat. Hazard ratios (HRs) and 90% CIs were calculated based on a stratified Cox proportional hazards regression model. A Cochran-Mantel-Haenszel test was used for the interaction in DCR among study groups. The presence of LM was assessed as a potential factor associated with OS, PFS, and DCR benefits using a test of interaction between treatment groups and the presence or absence of LM. Proportions between groups were compared with χ^2^ or Wilcoxon tests. SAS statistical software, version 9.0 (SAS Institute Inc), and R, version 3.6.3 (R Project for Statistical Analysis), were used for analyses. To be consistent with the statistical analysis for CCTG CO.26, a 2-sided *P* < .10 was considered statistically significant.

## Results

**Figure 1.**
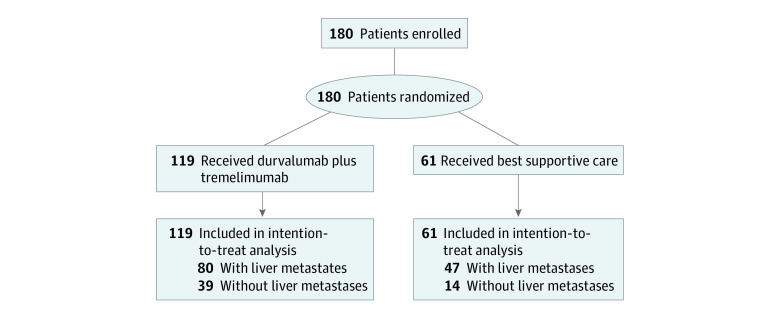
Study Flow Diagram

A total of 180 patients were enrolled and randomized, with 119 patients in the durvalumab plus tremelimumab group and 61 in the BSC group. The median age was 65 years (range, 36-87 years); there were 121 men (67.2%) and 59 women (32.8%). In terms of race and ethnicity, 19 patients (10.6%) were Asian, 151 (83.9%) were White, and 10 (5.6%) were of other race or ethnicity. At the time of study entry, LM were present in 127 patients (70.6%), including 80 of 119 (67.2%) in the durvalumab plus tremelimumab group and 47 of 61 (77.0%) in the BSC group (Figure 1). The demographics of groups with and without LM are presented in Table 1. There was a higher proportion of men with LM (94 of 127 [74.0%] vs 27 of 53 [50.9%]; *P* = .005). The median time from diagnosis to study entry was significantly longer in patients without LM than in those with LM (56 [range, 14-181] vs 40 [range, 8-153] months; *P* = .001). There were no differences in treatment history between patients with LM present or absent.

**Table 1.** Baseline Characteristics of the Intention-to-Treat Population

Characteristic	Patient group^a^		*P* value
	With LM (n = 127)	Without LM (n = 53)	
Age, median (range), y	64 (36-87)	68 (39-79)	.30
Sex			
Men	94 (74.0)	27 (50.9)	.005
Women	33 (26.0)	26 (49.1)	
Race and ethnicity			
Asian	10 (7.9)	9 (17.0)	.07
White	111 (87.4)	40 (75.5)	
Other^b^	6 (4.7)	4 (7.5)	
ECOG performance status^c^			
0	33 (26.0)	17 (32.1)	.47
1	94 (74.0)	36 (67.9)	
Time from initial cancer diagnosis, median (range), mo	40 (8-153)	56 (14-181)	.001
Study treatment group			
Durvalumab plus tremelimumab	80 (63.0)	39 (73.6)	.23
Best supportive care	47 (37.0)	14 (26.4)	
Prior systemic agents			
Fluoropyrimidine	127 (100)	53 (100)	>.99
Irinotecan hydrochloride	124 (97.6)	52 (98.1)	>.99
Oxaliplatin	110 (86.6)	44 (83.0)	.64
Anti-EGFR (if appropriate)	53 (41.7)	15 (28.3)	.10
Bevacizumab	104 (81.9)	39 (73.6)	.23
Regorafenib	32 (25.2)	15 (28.3)	.71
Tipiracil hydrochloride	0	0	>.99

Abbreviations: ECOG, Eastern Cooperative Oncology Group; EGFR, epidermal growth factor receptor; LM, liver metastases.

^a^ Unless otherwise indicated, data are expressed as No. (%) of patients.

^b^ Includes American Indian or Alaska Native, Black, Native Hawaiian or Other Pacific Islander, and unknown race or ethnicity.

^c^ Scores range from 0 to 5, with lower scores indicating fewer restrictions in daily activity.

Plasma tumor mutation burden was higher in patients with LM than in those without LM (median, 19.2 [IQR, 11.5-30.6] vs 12.3 [IQR, 7.7-20.6] mutations/Mb; *P* = .003) (eFigure in Supplement 2). Many common alterations associated with advanced colorectal cancer were detected, including loss of function events in *APC* (OMIM 611731), and hotspot *KRAS* (OMIM 190070) and *BRAF* (OMIM 164757) variations (V600E, D595G, and G469A). Among these genes, the only one that had a different frequency of alterations between groups was *APC* (eFigure in Supplement 2). Alterations in *APC* were seen in 109 patients with LM (85.8%) and 36 patients without LM (67.9%) (*P* = .03); however, *APC* alterations were also associated with higher TMB. There were 2 patients with MSI-H detected through baseline circulating tumor DNA, one in each treatment group.

**Figure 2.**
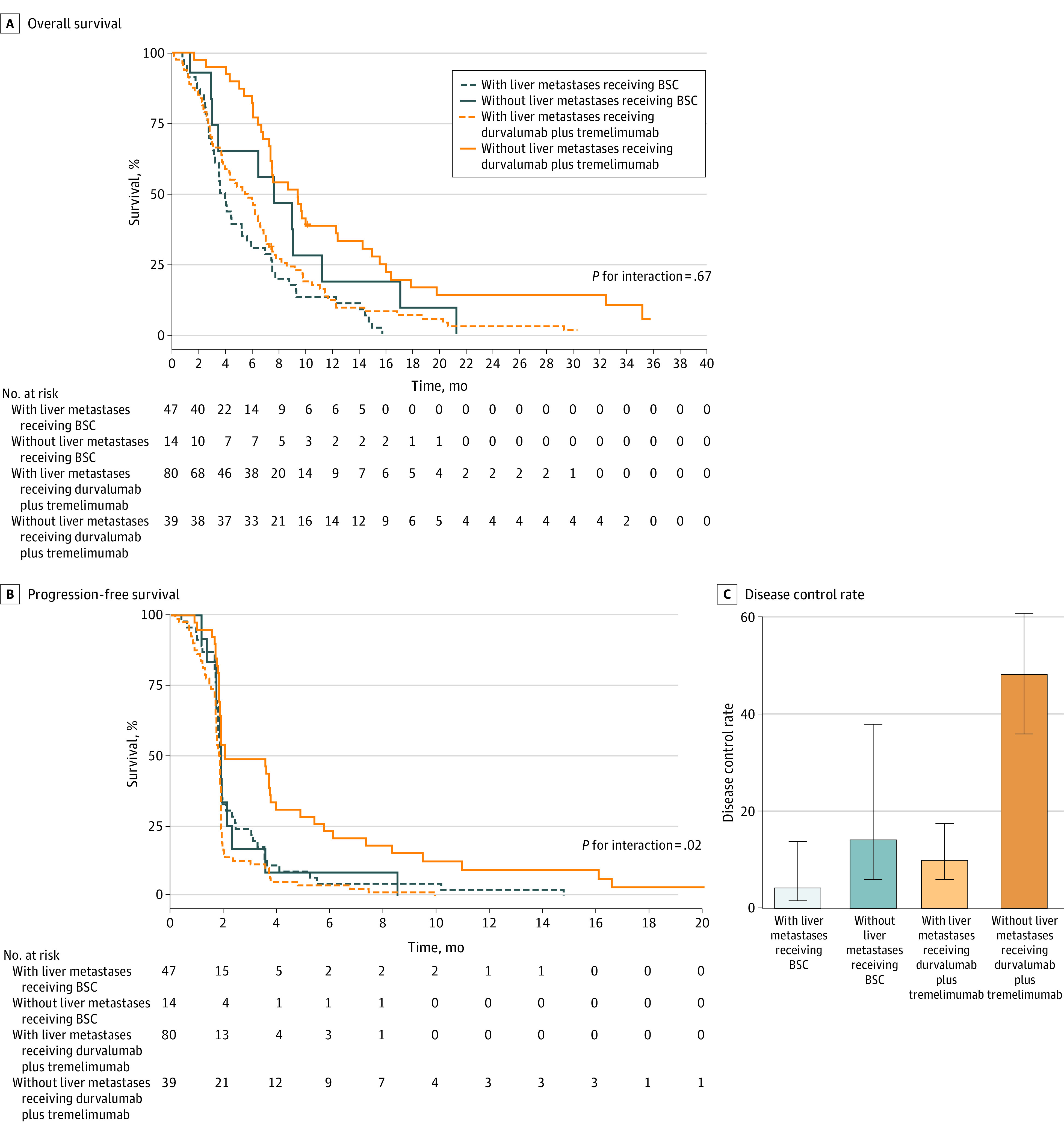
Survival by Treatment Groups and Presence of Liver Metastases Error bars indicate 90% CIs. BSC indicates best supportive care.

Among patients randomized to BSC, the median OS was 3.61 (90% CI, 3.15-4.47) months for patients with LM and 4.98 (90% CI, 1.91-9.00) for patients without LM. For patients in the durvalumab plus tremelimumab group, the median OS was 5.39 (90% CI, 3.81-6.24) months for patients with LM and 9.43 (90% CI, 7.39-10.18) months for patients without LM (Table 2 and Figure 2A). Among patients in the durvalumab plus tremelimumab group, the median PFS was 1.82 (90% CI, 1.71-1.84) months for patients with LM and 2.04 (90% CI, 1.87-3.71) months for patients without LM. For those receiving BSC, the median PFS was 1.84 (90% CI, 1.77-1.87) months for patients with LM and 1.87 (90% CI, 1.35-1.91) months for patients without LM (Table 2 and Figure 2B). The median DCR was 49% (90% CI, 36%-62%) in the durvalumab plus tremelimumab group and 14% (90% CI, 0-30%) in the BSC group for patients without LM and 10% (90% CI, 4%-16%) and 4% (90% CI, 0-9%), respectively, for patients with LM (Table 2 and Figure 2C).

**Table 2.** Univariate Analysis of OS and PFS by Treatment and Presence of LM

End point	Durvalumab plus tremelimumab		BSC		HR (90% CI)	OR	*P* value	*P* value for interaction
	No.	Median (90% CI)	No.	Median (90% CI)				
OS, mo								
With LM	80	5.39 (3.81-6.24)	47	3.61 (3.15-4.47)	0.79 (0.58-1.08)	NA	.22	.68
Without LM	39	9.43 (7.39-10.18)	14	4.98 (1.91-9.00)	0.65 (0.37-1.16)	NA	.22	
PFS, mo								
With LM	80	1.82 (1.71-1.84)	47	1.84 (1.77-1.87)	1.39 (1.02-1.90)	NA	.08	.02
Without LM	39	2.04 (1.87-3.71)	14	1.87 (1.35-1.91)	0.54 (0.35-0.96)	NA	.08	
DCR, %								
With LM	80	10 (4-16)	47	4 (0-9)	NA	2.50 (0.66-9.52)	.32	.48
Without LM	39	49 (36-62)	14	14 (0-30)	NA	5.70 (1.46-22.25)	.03	

Abbreviations: DCR, disease control rate; HR, hazard ratio; LM, liver metastases; NA, not applicable; OR, odds ratio; OS, overall survival; PFS, progression-free survival.

**Figure 3.**
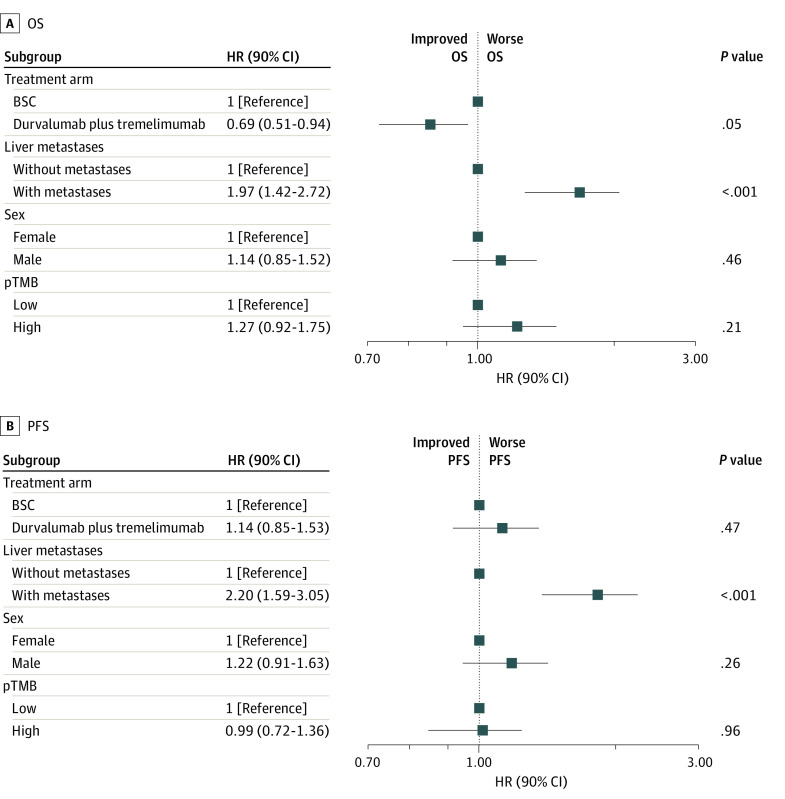
Subgroup Analysis of Overall Survival (OS) and Progression-Free Survival (PFS) in Multivariable Analysis BSC indicates best supportive care; HR, hazard ratio; pTMB, plasma tumor mutation burden.

On univariable analysis, there were no differences in OS between the durvalumab plus tremelimumab and BSC groups, regardless of LM, and a test of interaction was negative. Progression-free survival was improved with in the durvalumab plus tremelimumab group among patients without LM (HR, 0.54 [90% CI, 0.35-0.96]; *P* = .08; *P* = .02 for interaction) based on the trial’s statistical plan, including a 2-sided α = .10 (Table 2). The DCR was significantly higher in the durvalumab plus tremelimumab group among patients without LM (odds ratio, 5.70 [90% CI, 1.46-22.25]; *P* = .03). On multivariable analysis including sex and pTMB, patients without LM had significantly improved OS and PFS compared with patients with LM (Figure 3). Although durvalumab plus tremelimumab treatment was not associated with improved PFS in the multivariate analysis, OS was improved (HR, 0.69 [90% CI, 0.51-0.94]; *P* = .05).

## Discussion

Metastatic disease involving the liver is common in advanced solid malignant neoplasms. The proportion of patients with LM is particularly high in advanced colorectal cancer, with approximately 70% having liver involvement either alone or in combination with other sites of metastatic disease.^6,8,9,10^

The presence of LM is associated with unfavorable outcomes in multiple cancers.^11,12^ Although liver involvement has not been definitively shown to affect the efficacy of cytotoxic chemotherapy or targeted therapy,^9,13,14^ Cohen et al^15^ recently reported that liver involvement is a poor prognostic factor in advanced colorectal cancer. Its adverse impact increases as disease progresses. Furthermore, recent findings^7,16^ suggest that liver involvement in advanced colorectal cancer confers resistance to ICIs. In a single-arm, phase 2 study of regorafenib plus nivolumab in patients with advanced MSS-pMMR colorectal cancer, Fakih et al^7^ reported that the median OS, PFS, and overall response rate were higher in patients without LM compared with patients with LM. In a follow-up study,^16^ the combination of regorafenib, ipilimumab, and nivolumab appeared to be more active in patients without LM. In retrospective analyses, ICIs alone or in combination with other agents were reported to have improved outcomes in patients without LM for both MSI-H–dMMR and MSS-pMMR groups and in both first-line or refractory settings.^17,18,19,20^ In the recently reported LEAP-017 study,^10^ pembrolizumab and lenvatinib produced improved OS, PFS, and difference in overall response rate in patients without LM compared with investigators’ choice of regorafenib or trifluridine and tiparicil.

In addition to ICIs, the presence of LM may confer resistance to other immune-modulating agents. El-Khoueiry et al^21^ reported that the combination of botensilimab (an innate and/or adaptive immune activator) and balstilimab (an anti–programmed cell death protein 1 antibody) was more active in patients without LM. Our findings are consistent with these results; patients without LM had improved outcomes compared with patients with LM, regardless of treatment, and durvalumab plus tremelimumab appeared to be more active in patients without LM. Overall survival was numerically improved with durvalumab plus tremelimumab in patients without LM. Our analysis had limited statistical power since there were only 53 patients without LM. Studies reporting differential impacts with regard to the presence of LM are heterogeneous. It is possible that different ICIs may have different activity levels, and our results may be specific to durvalumab plus tremelimumab.

Patients with LM have been reported to respond poorly to ICIs in other solid malignant neoplasms such as melanoma and non–small-cell lung cancer,^22,23^ and the mechanisms of resistance to ICIs in this patient population are poorly understood. The liver is postulated to be an immunoprivileged site, resulting in tolerance to various antigens. Yu et al^24^ reported an increase in CD11b^+^F4/80^+^ myeloid cells in the livers of mice with LM, leading to increased apoptosis of antigen-specific CD8^+^ T cells and diminished numbers of these cells in the tumor microenvironment. Hou et al^25^ showed that the presence of LM increased tumor-induced CD45^−^Ter119^+^CD71^+^ erythroid progenitor cells, leading to overproduction of artemin, a neutrophic peptide. Artemin promotes tumor progression and resistance to ICIs by activating the rearranged during transfection kinase. Interestingly, both groups showed that liver-directed radiotherapy could restore sensitivity to ICIs by eliminating these immunosuppressive macrophages. A phase 2 study of durvalumab and tremelimumab with concurrent radiotherapy in advanced MSS-pMMR colorectal cancer^26^ did not meet its prespecified end points; however, only 29% of patients received radiotherapy to the liver. Interestingly, 1 of 2 partial responses observed in the study occurred in a patient who received radiotherapy to the liver. Lee et al^27^ reported that LM-associated immune suppression was antigen-specific and mediated through the activation of regulatory T cells and modulation of intratumoral CD11b^+^ monocytes. Depletion of regulatory T cells through anti–cytotoxic T-cell lymphocyte antigen-4 inhibition can restore the dysfunctional immune state.

Although pTMB was significantly higher in patients with LM, there were no significant differences in alterations of genes commonly found in advanced colorectal cancer other than *APC*. On multivariate analysis, pTMB was not associated with either PFS or OS. Plasma tumor mutational burden has been shown to be discordant with tissue TMB.^28^ There is a lack of standardization in pTMB assays across different platforms, and pTMB has not been approved as a biomarker for response to ICIs, in contrast to tissue TMB.^29^ Patients without LM had longer intervals from diagnosis to study entry, consistent with better OS observed for this group of patients in this analysis.

Recently, ICIs have shown significant efficacy and changed the treatment landscapes in hepatocellular carcinoma and cholangiocarcinoma.^30,31,32^ Hepatocellular carcinoma and cholangiocarcinoma arise as a result of chronic liver damage, and it is possible that these diseases have different immunological environments compared with the setting of metastatic disease to the otherwise normal liver.

Previous reports of the lack of efficacy of ICIs in patients with LM and advanced colorectal cancer are from single-arm phases 1 and 2 studies^7,16,21^ or retrospective case series with a limited number of patients.^17,18,19,20^ The current analysis was based on a randomized phase 2 study with a control arm of BSC. Our findings are consistent with those from the recently reported randomized phase 3 LEAP-017 study.^10^ These findings have significant implications for the development of ICIs and design of future clinical trials evaluating ICIs in MSS-pMMR advanced colorectal cancer. The presence of LM should be considered a stratifying factor. It might be reasonable to exclude patients with LM from trials evaluating ICIs in advanced colorectal cancer unless these trials include specific strategies to overcome this resistance. Future research should focus on understanding the mechanisms underlying resistance to ICIs in the presence of LM.

### Limitations

This study has some limitations. Our analysis was unplanned and exploratory in nature. Additionally, it had limited statistical power because there were only 53 patients without LM. Studies reporting differential impact with regard to the presence of LM are heterogeneous. It is possible that different ICIs may have different activity levels and our findings may be specific to durvalumab plus tremelimumab.

## Conclusions

In this secondary analysis of the CCTG CO.26 trial, LMs were associated with worse survival outcomes among patients with advanced colorectal cancer. Liver metastases should be considered in the design and interpretation of future clinical studies evaluating ICI therapy.

## Notes

Supplement 1.
Trial Protocol

Supplement 2. **eFigure.** Comparisons of pTMB and Common Gene Alterations Between Patients With and Without Liver Metastases

Supplement 3.
Data Sharing Statement
