# Supplementary material for: Liver Metastases and Immune Checkpoint Inhibitor Efficacy in Patients With Refractory Metastatic Colorectal Cancer: A Secondary Analysis of a Randomized Clinical Trial
Source: JAMA Netw Open. 2023 Dec 5;6(12):e2346094. doi: 10.1001/jamanetworkopen.2023.46094 (PMC10698621; doi:10.1001/jamanetworkopen.2023.46094)
Supplement: Supplement 1. — Trial Protocol [file jamanetwopen-e2346094-s001.pdf]

PROTOCOL DATE: 2016-JUL-20
CCTG TRIAL: CO.26

HEALTH CANADA SUBMISSION
ADMINISTRATIVE UPDATE #1: 2018-JAN-24

ADMINISTRATIVE UPDATE #2: 2018-JAN-31

CANADIAN CANCER TRIALS GROUP (CCTG)

A PHASE II RANDOMIZED STUDY OF DURVALUMAB AND TREMELIMUMAB AND BEST SUPPORTIVE CARE VS BEST SUPPORTIVE CARE ALONE IN PATIENTS WITH ADVANCED COLORECTAL ADENOCARCINOMA REFRACTORY TO STANDARD THERAPIES

CCTG Protocol Number: **CO.26**

STUDY CHAIR:	Eric Chen
TRIAL COMMITTEE:	Derek Jonker<br>Hagen Kennecke<br>Sheryl Koski
SENIOR INVESTIGATOR:	Chris O'Callaghan
BIOSTATISTICIAN:	Dongsheng Tu
QUALITY OF LIFE COORDINATOR:	Alice Wei
STUDY COORDINATOR:	Nadine Magoski
REGULATORY SPONSOR:	CCTG
SUPPORTED BY:	AstraZeneca

(For contact information of study personnel see Final Page.)

TABLE OF CONTENTS

STUDY ACKNOWLEDGMENT/DISCLOSURE (SA/D) ... 1

TREATMENT SCHEMA ... 2

1.0 OBJECTIVES ... 3
1.1 Primary Objective ... 3
1.2 Secondary Objectives ... 3
1.3 Tertiary Objectives ... 3

2.0 BACKGROUND INFORMATION AND RATIONALE ... 4

3.0 BACKGROUND THERAPEUTIC INFORMATION ... 10
3.1 Durvalumab ... 10
3.2 Tremelimumab ... 11
3.3 Fixed Dosing in Durvalumab and Tremelimumab ... 13

4.0 STUDY POPULATION ... 14
4.1 Eligibility Criteria ... 14
4.2 Ineligibility Criteria ... 18

5.0 PATIENT EVALUATION FLOWSHEET: PRE-TREATMENT, ON STUDY, AND AFTER TREATMENT ... 20
5.1 Patient Evaluation Flowsheet: Pre-study (Baseline) and Post-randomization Follow-up ... 20
5.2 Patient Evaluation Flowsheet: Pre-Treatment, On-Study, and after Protocol Treatment ... 22
5.3 Follow-up for Ineligible Patients ... 24

6.0 ENTRY/RANDOMIZATION PROCEDURES ... 25
6.1 Entry Procedures ... 25
6.2 Stratification ... 25
6.3 Randomization ... 26

7.0 TREATMENT PLAN ... 27
7.1 Protocol Treatment Plan ... 27
7.2 Concomitant Therapy ... 31

8.0 CRITERIA FOR MEASUREMENT OF STUDY ENDPOINTS ... 33
8.1 Definitions ... 33
8.2 Response and Evaluation Endpoints ... 33
8.3 Response Duration ... 38
8.4 Stable Disease Duration ... 38
8.5 Methods of Measurement ... 38

9.0 SERIOUS ADVERSE EVENT REPORTING ... 40
9.1 Definition of a Reportable Serious Adverse Event *(Durvalumab +/- Tremelimumab Arm)* ... 40
9.2 Serious Adverse Event Reporting Instructions ... 42
9.3 Other Protocol Reportable Events – Pregnancy Reporting and Exposure Reporting ... 43
9.4 CCTG Responsibility for Reporting Serious Adverse Events to Health Canada ... 44
9.5 CCTG Reporting Responsibility to AstraZeneca ... 44
9.6 AstraZeneca Reporting Responsibilities ... 44
9.7 Reporting Safety Reports to Investigators ... 44

10.0 PROTOCOL TREATMENT DISCONTINUATION AND THERAPY AFTER STOPPING 46
10.1 Criteria for Discontinuing Protocol Treatment 46
10.2 Duration of Protocol Treatment 46
10.3 Therapy After Protocol Treatment is Stopped 46

11.0 CENTRAL REVIEW PROCEDURES 47
11.1 Central Radiology Review 47
11.2 Central Pathology Review 47
12.0 CORRELATIVE STUDIES 48
12.1 Protocol-Mandated Correlative Studies 48
12.2 Optional Banking of Samples 50

13.0 STATISTICAL CONSIDERATIONS 51
13.1 Objectives and Design 51
13.2 Study Endpoints and Analysis 51
13.3 Sample Size and Duration of Study 52
13.4 Safety Monitoring 52
13.5 Interim Analysis 52

14.0 PUBLICATION POLICY 53
14.1 Authorship of Papers, Meeting Abstracts, Etc. 53
14.2 Responsibility for Publication 53
14.3 Submission of Material for Presentation or Publication 53

15.0 ETHICAL, REGULATORY AND ADMINISTRATIVE ISSUES 54
15.1 Regulatory Considerations 54
15.2 Inclusivity in Research 54
15.3 Obtaining Informed Consent 55
15.4 Discontinuation of the Trial 56
15.5 Retention of Patient Records and Study Files 56
15.6 Centre Performance Monitoring 56
15.7 On-Site Monitoring/Auditing 57
15.8 Case Report Forms 57

16.0 REFERENCES 58

APPENDIX I - PERFORMANCE STATUS SCALES/SCORES 61
APPENDIX II - DRUG DISTRIBUTION, SUPPLY AND CONTROL 62
APPENDIX III - DOSE MODIFICATION AND TOXICITY MANAGEMENT GUIDELINES FOR IMMUNE-MEDIATED, INFUSION RELATED AND NON IMMUNE-MEDIATED REACTIONS 63
APPENDIX IV - DOCUMENTATION FOR STUDY 63
APPENDIX V - NCI COMMON TERMINOLOGY CRITERIA FOR ADVERSE EVENTS 88
APPENDIX VI - QUALITY OF LIFE ASSESSMENT 89

LIST OF CONTACTS .... Final Page

STUDY ACKNOWLEDGMENT/DISCLOSURE (SA/D)

I understand that this protocol contains information that is confidential and proprietary to CCTG and AstraZeneca.

I have read the protocol and agree that it contains all necessary details for carrying out the study as described. I will conduct this protocol as outlined therein, and according to Good Clinical Practice and any applicable local regulations. I will make a reasonable effort to complete the study within the time designated. I confirm that I and study personnel participating under my supervision have adequate resource to fulfill their responsibilities as outlined in this protocol. I will maintain documentation of any investigator responsibilities assigned to participating study personnel. I confirm that all data will be submitted in a timely manner and will be accurate, complete and supported by source documents. I will complete any protocol specific training required by the sponsor and that I understand the requirement to inform additional site personnel with delegated duties of this information.

I will provide copies of the protocol and access to all information furnished by CCTG and CCTG and AstraZeneca to study personnel under my supervision. I will discuss this material with them to ensure that they are fully informed about the investigational product and the study.

I understand that this trial will be registered on a public trial registry and that my contact information and site name will be included in the registry listing.

I will provide protocol information to my Research Ethics Board (REB), Institutional Review Board(s) [IRB(s)] or Independent Ethics Committee(s) [IEC(s)], subject to the following condition: The contents of this protocol may not be used in any other clinical trial and may not be disclosed to any other person or entity without the prior written permission of AstraZeneca and CCTG. The foregoing shall not apply to disclosure required by governmental regulations or laws; however, I will give prompt notice to AstraZeneca and CCTG of any such disclosure.

I understand that I may terminate or suspend enrolment of the study at any time if it becomes necessary to protect the best interests of the study subjects, however I will give prompt notice to CCTG. The study may be terminated at any time by CCTG or AstraZeneca with or without cause.

Any supplemental information that may be added to this document is also confidential and proprietary to AstraZeneca and CCTG and must be kept in confidence in the same manner as the contents of this protocol.

____ ___________________________

Qualified Investigator (printed name and signature) Date

Protocol Number: CCTG CO.26

CENTRE: ____

TREATMENT SCHEMA

This is a multi-centre, phase II study of doublet immunotherapy (durvalumab + tremelimumab) and best supportive care *versus* best supportive care only in patients with advanced colorectal cancer who are refractory to all available therapy, conducted by the Canadian Cancer Trials Group with the support of AstraZeneca.

Patients with advanced CRC, refractory to all available therapy → Randomize 1:2

↗ ARM 1:
Best Supportive Care
N=60

↘ ARM 2:
Durvalumab + Tremelimumab*
and Best Supportive Care
N=120

Sample Size: 180
Primary Endpoint: Overall Survival

* *Tremelimumab and Durvalumab every 4 weeks for 4 cycles (1 cycle = 4 weeks (28 days), followed by Durvalumab monotherapy to objective disease progression. See Section 7 for details.*

Patients will be stratified by:

- ECOG Performance Status: 0 *vs* 1
- Site of tumour:
  - right colon (caecum, ascending colon, hepatic flexure) *vs*
  - transverse colon *vs*
  - left colon (within the splenic flexure, descending colon, sigmoid colon, or rectosigmoid junction) *vs*
  - rectum *vs*
  - unknown

1.0 OBJECTIVES

1.1 Primary Objective

The primary objective is to determine the effect on overall survival (OS) of the combination of durvalumab and tremelimumab and best supportive care *versus* best supportive care only in patients with refractory, advanced colorectal cancer.

1.2 Secondary Objectives

- To determine the effect on progression-free survival (PFS) of the combination of durvalumab and tremelimumab and best supportive care versus best supportive care only in patients with refractory, advanced colorectal cancer.
- To assess the toxicity and safety of the combination of durvalumab and tremelimumab and best supportive care in patients with refractory, advanced colorectal cancer.
- To determine the effect on objective response rate (ORR) of the combination of durvalumab and tremelimumab and best supportive care *versus* best supportive care only in patients with refractory, advanced colorectal cancer.

1.3 Tertiary Objectives

- To determine the effect on quality of life (QoL) of the combination of durvalumab and tremelimumab and best supportive care *versus* best supportive care only in patients with refractory, advanced colorectal cancer.
- To determine the effect of tumour PD-L1 expression assessed by IHC on efficacy of the combination of durvalumab and tremelimumab and best supportive care *versus* best supportive care only in patients with refractory, advanced colorectal cancer.
- To explore association between putative biomarkers in archival tumour specimens and the potential for clinical benefit between the combination of durvalumab and tremelimumab and best supportive care *versus* best supportive care only in patients with refractory, advanced colorectal cancer.
- To explore association with baseline values and changes in putative biomarkers in blood, serum and plasma and the potential for clinical benefit between the combination of durvalumab and tremelimumab and best supportive care *versus* best supportive care only in patients with refractory, advanced colorectal cancer.

2.0 BACKGROUND INFORMATION AND RATIONALE

Colorectal Cancer

Cancer of the colon and rectum is the 2nd leading cause of cancer death in Canada *[Canadian Cancer Society Statistics 2015]*. Each year, there are 25,100 new cases and 9100 deaths from colorectal cancer. For patients with advanced colorectal cancer, the overall survival is approximately 8-10 months without treatment. Over the last 10-15 years, there have been unprecedented improvements in the overall survivals of patients with advanced colorectal cancer, with median survivals of approaching 30 months *[Venook 2014]*. These improvements in survival are a result of increased availability of drugs, including cytotoxic chemotherapeutic agents (5-FU, irinotecan, oxaliplatin, TAS-102), those targeting the VEGF pathway (bevacizumab, aflibercept, ramucirumab, and regorafenib) and those targeting the EGFR pathway (cetuximab and panitumumab).

Unfortunately, the majority of patients with advanced colorectal cancer will succumb to their disease, with 5-year survival being only approximately 5%. Once they progress on standard therapies, the estimated overall survival for patients with advanced colorectal cancer is 5-6 months with best supportive care *[Grothey 2013; Mayer 2015]*. In this patient population, treatment with regorafenib or TAS-102 leads to improvement in the median overall survival of only 2.5 months or 1.8 months respectively. Therefore, new treatment options/strategies are urgently needed in patients with advanced colorectal cancer who progressed on standard therapies.

Immunotherapy & Immune Checkpoint Inhibitors

Immune responses directed against tumours are one of the body's natural defenses against the growth and proliferation of cancer cells. However, over time and under pressure from constant immune attacks, cancers develop strategies to evade the immune system allowing them to develop unchecked. One such mechanism involves upregulation of surface proteins that deliver inhibitory signals to cytotoxic T cells. Programmed cell death ligand 1 (PD-L1) is one such protein, and it is upregulated in a broad range of cancers with a high frequency, with up to 88% expression in some tumour types. In a number of these cancers, including lung *[Mu 2011]*, renal *[Thompson 2005; Thompson 2006; Krambeck 2007]*, pancreatic *[Nomi 2007; Loos 2008; Wang 2010],* ovarian cancer *[Hamanishi 2007]*, and hematologic malignancies *[Andorsky 2011; Brusa 2013]*, tumour cell expression of PD-L1 is associated with reduced survival and an unfavorable prognosis.

PD-L1 is part of a complex system of receptors and ligands that are involved in controlling T-cell activation. It acts at multiple sites in the body to help regulate normal immune responses and is utilized by tumours to help evade detection and elimination by the host immune system. In the lymph nodes, PD-L1 on antigen-presenting cells binds to PD-1 or CD80 on activated T cells and delivers an inhibitory signal to the T cell *[Keir 2008; Park 2010]*. This results in reduced T-cell activation and fewer activated T cells in circulation. In the tumour microenvironment, PD-L1 expressed on tumour cells binds to PD-1 and CD80 on activated T cells reaching the tumour. This delivers an inhibitory signal to those T cells, preventing them from killing target cancer cells and protecting the tumour from immune elimination *[Zou 2008]*.

Blockade of PD-1 engagement with its ligand PD-L1 induces immune responses in vitro and has been shown to mediate anticancer activity preclinically *[Fife 2009]*. Clinically, blockade of the PD-1 inhibitory checkpoint pathway by inhibiting PD-L1/ PD-1 engagement has been shown to induce tumour regression across many cancer types, including melanoma and renal cell, colon and lung cancers *[Pardoll 2012; Brahmer 2012]*. Single agent immunotherapy with anti-PD-1 or anti-PDL-1 antibodies across many tumour types has been generally well tolerated, with common drug related adverse events mainly limited to grade 1 or 2 fatigue, diarrhea, rash, pruritus, nausea and decreased appetite. Immune-related adverse events are uncommon (< 2%), and include pneumonitis, vitiligo, colitis, hepatitis and hypophysitis and thyroiditis *[Antonia 2014b]*.

CTLA-4 is another co-inhibitory receptor expressed on activated T cells and regulates early stage T cell activation, reducing the amplitude of T-cell activation. Inhibition of CTLA-4 signaling is a validated approach to cancer therapy, as shown by the approval in 2011 of ipilimumab for the treatment of metastatic melanoma based on statistically significant and clinically meaningful improvement in overall survivals of patients with advanced melanoma *[Hodi 2010; Robert 2011]*.

In general, tumour response rates to anti-CTLA-4 therapy are low (~10%). However, in patients who respond, the responses are generally durable, lasting several months even in patients with aggressive tumours such as refractory metastatic melanoma. Because these agents work through activation of the immune system and not by directly targeting the tumour, responses can occur late and some patients may have perceived progression of their disease in advance of developing disease stabilization or a tumour response. In some cases, early growth of pre-existing lesions or the appearance of new lesions may have been due to immune-cell infiltration into the tumour and not due to proliferation and extension of neoplastic cells, per se *[Wolchok 2009]*. Overall, although the impact on conventionally-defined PFS can be small, durable response or stable disease seen in a proportion of patients can lead to significant prolongation of OS. The melanoma data with ipilimumab clearly demonstrate that a small proportion of patients with an objective response had significant prolongation of OS, supporting the development of this class of agents in other tumours. Although Phase 2 and Phase 3 studies of tremelimumab in metastatic melanoma did not meet the primary endpoints of response rate and OS respectively, the data suggest activity of tremelimumab in melanoma *[Kirkwood 2010; Ribas 2013]*. In a large Phase 3 randomized study comparing tremelimumab with dacarbazine (DTIC)/temozolomide in patients with advanced melanoma, the reported median OS in the final analysis was 12.6 months for tremelimumab versus 10.7 months for DTIC/temozolomide (HR = 1.14; p=0.13) *[Ribas 2013]*.

Tremelimumab

Tremelimumab is a human IgG2 monoclonal antibody directed against the T-cell receptor protein cytotoxic T-lymphocyte-associated protein 4 (CTLA4) *[Tarhini 2013]*. Tremelimumab binds to CTLA4 and blocks the binding of the antigen-presenting cell ligands B7-1 and B7-2 to CTLA4, resulting in inhibition of B7-CTLA4-mediated downregulation of T-cell activation. In addition, blockade of CTLA-4 binding to B7 by anti-CTLA-4 antibodies results in markedly enhanced T-cell activation and anti-tumour activity in animal models, including killing of established murine solid tumours and induction of protective anti-tumour immunity. Therefore, it is expected that treatment with an anti-CTLA-4 antibody, such as tremelimumab, will lead to increased activation of the human immune system, increasing anti-tumour activity in patients with solid tumours.

An extensive program of non-clinical and clinical studies has been conducted for tremelimumab both as monotherapy and combination therapy with conventional anticancer agents to support various cancer indications using different dose schedules. As of the data cut-off date of 12 November 2014 (for all studies except D4190C00006, which has a cut-off date of 4 December 2014), 973 patients have received tremelimumab monotherapy (not including 497 patients who have been treated in the blinded phase IIb study, D4880C00003) and 208 patients have received tremelimumab in combination with other agents.

Tremelimumab exhibited a biphasic PK profile with a long terminal phase half-life of 22 days. Overall, a low incidence of ADAs (<6%) was observed for treatment with tremelimumab.

Durvalumab

Durvalumab is a human monoclonal antibody (MAb) of the immunoglobulin G1 kappa (IgG1κ) subclass that inhibits binding of programmed cell death ligand 1 (PD-L1) (B7 homolog 1 [B7-H1], cluster of differentiation [CD]274) to programmed cell death 1 (PD-1; CD279) and CD80 (B7-1). Durvalumab is composed of 2 identical heavy chains and 2 identical light chains, with an overall molecular weight of approximately 149 kDa. Durvalumab contains a triple mutation in the constant domain of the immunoglobulin (Ig) G1 heavy chain that reduces binding to complement protein C1q and the fragment crystallizable gamma (Fcγ) receptors involved in triggering effector function.

Durvalumab binds with high affinity and specificity to human PD-L1 and blocks its interaction with PD-1 and CD80. *In vitro* studies demonstrate that durvalumab antagonizes the inhibitory effect of PD-L1 on primary human T cells, resulting in their restored proliferation and release of interferon gamma (IFN-γ). Additionally, durvalumab demonstrated a lack of antibody-dependent cell-mediated cytotoxicity (ADCC) and complement-dependent cytotoxicity (CDC) in cell-based functional assays. *In vivo* studies show that durvalumab inhibits tumour growth in a xenograft model via a T lymphocyte (T-cell) dependent mechanism. Moreover, an anti-mouse PD-L1 antibody demonstrated improved survival in a syngeneic tumour model when given as monotherapy and resulted in complete tumour regression in > 50% of treated mice when given in combination with chemotherapy.

Combination of Durvalumab and Tremelimumab

Targeting both PD-1 and CTLA-4 pathways may have additive or synergistic activity because the mechanisms of action of CTLA-4 and PD-1 are non-redundant *[Pardoll 2014]*. In fact, combining immunotherapy agents has been shown to result in improved response rates (RRs) relative to monotherapy. For example, the concurrent administration of nivolumab and ipilimumab to patients with advanced melanoma induced higher objective response rates (ORRs) than those obtained with single-agent therapy *[Larkin 2015]*. Importantly, responses appeared to be deep and durable *[Wolchok 2014]*. Similar results have been observed in an ongoing study of durvalumab + tremelimumab in NSCLC *[Antonia 2014a]*.

Study D4190C00006 is a phase Ib dose-escalation study to establish safety, PK/PDx, and preliminary anti-tumour activity of durvalumab + tremelimumab combination therapy in patients with advanced NSCLC. The dosing schedule utilized is durvalumab every 2 weeks (q2w) or every 4 weeks (q4w) up to Week 50 and 48 (12 months), combined with tremelimumab q4w up to Week 24 for 7 doses then every 12 weeks for 2 additional doses for up to 12 months. Objectives of this study include evaluating the safety profiles of the combination of durvalumab and tremelimumab, collecting preliminary evidence of anti-tumour effects and selecting an optimal combination dose of durvalumab and tremelimumab that would yield sustained target suppression (sPD-L1). The study is ongoing and continues to accrue.

As of 24 October 2016, a total of 835 patients provided evaluable samples for ADA analysis. Overall, 26 of 835 patients (3.1%) tested positive for treatment-emergent ADAs in the ADA evaluable population. Three patients (0.4%, in 3/835 patients) were neutralizing ADA (nAb) positive. Based on population PK covariate analysis, ADA positive status was not associated with a clinically relevant reduction of exposure to durvalumab. At the 10 mg/kg Q2W dose, sPD-L1 suppression in ADA positive patients was similar to that observed in ADA negative patients.

In order to reduce the dosing frequency of durvalumab to align with the q4w dosing of tremelimumab, while ensuring an acceptable PK/PDx, safety, and efficacy profile, cohorts were narrowed to 15 and 20 mg/kg durvalumab q4w. PK simulations from the durvalumab monotherapy data indicated that a similar area under the plasma drug concentration-time curve at steady state (AUCss; 4 weeks) was expected following both 10 mg/kg q2w and 20 mg/kg q4w durvalumab. The observed durvalumab PK data from the D4190C00006 study were well in line with the predicted monotherapy PK data developed preclinically. This demonstrates similar exposure of durvalumab 20 mg/kg q4w and 10 mg/kg q2w, with no alterations in PK when durvalumab and tremelimumab (doses ranging from 1 to 3 mg/kg) are dosed together. While the median Cmax at steady state (Cmax,ss) is expected to be higher with 20 mg/kg q4w (approximately 1.5 fold) and median trough concentration at steady state (Ctrough,ss) is expected to be higher with 10 mg/kg q2w (approximately 1.25 fold), this is not expected to impact the overall safety and efficacy profile, based on existing preclinical and clinical data.

Monotonic increases in PDx activity were observed with increasing doses of tremelimumab relative to the activity observed in patients treated with durvalumab monotherapy. There was evidence of augmented PDx activity relative to durvalumab monotherapy with combination doses containing 1 mg/kg tremelimumab, inclusive of both the 15 and 20 mg/kg durvalumab plus 1 mg/kg tremelimumab combinations.

Patients treated with doses of tremelimumab above 1 mg/kg had a higher rate of adverse events (AEs), including discontinuations due to AEs, serious AEs (SAEs), and severe AEs. Between the 10 mg/kg durvalumab + 1 mg/kg tremelimumab and 10 mg/kg durvalumab + 3 mg/kg tremelimumab cohorts treated at the q2w schedule, the number of patients reporting any AE, Grade 3 AEs, SAEs, and treatment-related AEs was higher in the 10 mg/kg durvalumab + 3 mg/kg tremelimumab cohort than the 10 mg/kg durvalumab + 1 mg/kg tremelimumab cohort. A similar pattern was noted in the q4w regimens, suggesting that, as the dose of tremelimumab increased above 1 mg/kg, a higher rate of treatment-related events may be anticipated. Further, the SAEs frequently attributed to immunotherapy, pneumonitis and colitis, were more commonly seen in cohorts using either 3 or 10 mg/kg of tremelimumab compared to the 1-mg/kg dose cohorts. Together, these data suggest that a combination using a tremelimumab dose of 1 mg/kg appeared to minimize the rate of toxicity when combined with durvalumab. As a result, all combination doses utilizing either the 3 or 10 mg/kg doses of tremelimumab were eliminated in the final dose selection.

In contrast, cohorts assessing higher doses of durvalumab with a constant dose of tremelimumab did not show an increase in the rate of AEs. The data suggested that increasing doses of durvalumab may not impact the safety of the combination as much as the tremelimumab dose. Further, safety data between the 10-mg/kg and 20-mg/kg cohorts were similar, with no change in safety events with increasing dose of durvalumab.

In Study D4190C00006, of all treatment cohorts, the cohort of 11 patients treated in the 20 mg/kg durvalumab + 1 mg/kg tremelimumab group had the fewest AEs, Grade ≥ 3 AEs, SAEs, and treatment discontinuations due to AEs, but still showed strong evidence of clinical activity. This cohort had a lower number of treatment-related Grade ≥ 3 AEs or treatment-related SAEs. No dose-limiting toxicities were reported.

Preliminary clinical activity of the durvalumab and tremelimumab combination did not appear to change with increasing doses of tremelimumab. The 15 and 20 mg/kg durvalumab q4w cohorts demonstrated objective responses at all doses of tremelimumab, and increasing doses of tremelimumab did not provide deeper or more rapid responses.

Efficacy data suggested that the 20 mg/kg durvalumab + 1 mg/kg tremelimumab dose cohort may demonstrate equivalent clinical activity to other dose combinations. A total of 5 of 11 patients in the 20 mg/kg durvalumab + 1 mg/kg tremelimumab cohort were evaluable for efficacy with at least 8 weeks of follow-up. Of these, there were 2 patients (40%) with partial response (PR), 1 patient (20%) with stable disease (SD), and 1 patient (20%) with progressive disease (PD). (The fifth patient had only a single scan, which was conducted outside the window for these evaluations.)

Additionally, of all cohorts, the 20 mg/kg durvalumab + 1 mg/kg tremelimumab dose cohort had the fewest AEs, Grade ≥ 3 AEs, SAEs, and treatment discontinuations due to AEs, but still showed some evidence of clinical activity. Altogether, the data suggested that a 20 mg/kg durvalumab + 1 mg/kg tremelimumab dose combination should be selected for further development.

In summary, combinations of durvalumab and tremelimumab appear tolerable at doses of durvalumab 20 mg/kg q4w and tremelimumab 1 mg/kg q4w. Higher doses did not result in greater antitumour activity but were generally associated with higher rates of AEs. Related Grade 3/4 events were reported in 4/18 (22%) patients, while the most frequently reported events were diarrhea, pruritus, rash, and elevated AST/ALTs (11% for each AE). Only one patient discontinued study therapy due to drug-related AEs *[Antonia 2015]*.

Immune Therapy in Advanced Colorectal Cancer

The role of immune therapy in CRC has recently been reported. PD-1 positive lymphocytes are present in 47% of CRC *[Gatalica 2014]*. Overexpression of PD-L1 in CRC is associated with poor prognosis *[Song 2013]*. Chemotherapy induced PD-1 expression has been associated with improved PFS in CRC suggesting an indirect chemotherapy induced antitumour immune response *[Formica 2013]*. Microsatellite instability (MSI-H), a feature of a subset of CRC, is also associated with enhanced CLTA-4 and PD1 expression *[Llosa 2015]*.

A recent publication in the New England Journal of Medicine (NEJM) *[Le 2015]* demonstrated that the anti PD-1 antibody pembrolizumab has significant activity in MSI-H CRC, which makes up less that 5% of patients with advanced refractory disease. In this trial, 78% of MSI-H patients had disease control, There was superior OS (HR 0.22) and PFS (HR 0.10) in the MSI-H vs MSI-S patients (see Figures A & B below).

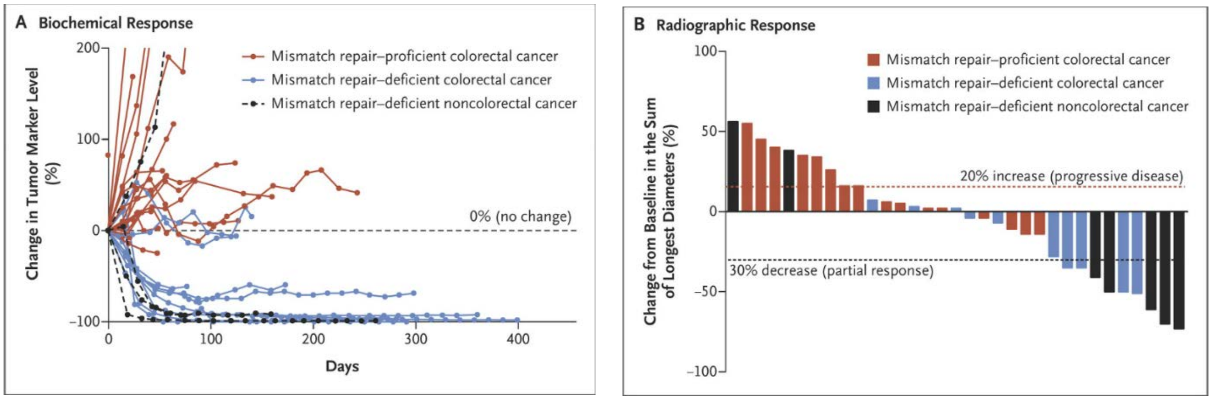

Figure 1: Biochemical (A) and Radiographic (B) responses in patients with colorectal cancer treated with the anti-PD-1 antibody pembrolizumab *[Le 2015]*.

Further exploration of the value of immune therapy in CRC is desirable, given the large unmet need.

Despite the exciting results of pembrolizumab in patients with advanced MSI-H colorectal cancer, inhibiting PD-1/PD-L1 interaction alone is likely of limited therapeutic value in colorectal cancer since only 5% patients with advanced colorectal cancer harboring MSI-H. Targeting both PD-1/PD-L1 and CTLA-4 pathways may have additive or synergistic activity because the mechanisms of action of CTLA-4 and PD-1/PD-L1 inhibition are non-redundant. This study is designed to evaluate whether combining PD-1/PD-L1 and CTLA-4 inhibition will lead to improved patient survivals compared to best supportive care in advanced colorectal cancer, regardless of MSI status.

3.0 BACKGROUND THERAPEUTIC INFORMATION

3.1 Durvalumab

3.1.1 *Name and Chemical Information*

Durvalumab is a human monoclonal antibody of the immunoglobulin G1 kappa (IgG1κ) subclass that inhibits binding of programmed cell death ligand (PD-L1) (B7 homolog 1[B7-H1], cluster of differentiation [CD]274 to programed cell death 1 (PD-1; CD279) and CD80 (B7).
See the current durvalumab Investigator Brochure for additional details and the most up to date information.

3.1.2 *Chemical Structure*

Durvalumab is composed of 2 identical heavy chains and 2 identical light chains, with an overall molecular weight of approximately 149 kDa. Durvalumab contains a triple mutation in the constant domain of the immunoglobulin (Ig) G1 heavy chain that reduces binding to complement protein C1q and the fragment crystallizable gamma (Fcγ) receptors involved in triggering effector function.

3.1.3 *Mechanism of Action*

Durvalumab binds with high affinity and specificity to human PD-L1 and blocks its interaction with PD-1 and CD80. In vitro studies demonstrate that durvalumab antagonizes the inhibitory effect of PD-L1 on primary human T cells, resulting in their restored proliferation and release of interferon gamma (IFN-γ).

3.1.4 *Experimental Antitumour Activity*

- In a xenograft model durvalumab inhibited human tumour growth via a T-cell-dependent mechanism.
- An anti-mouse PD-L1 antibody demonstrated improved survival in a syngeneic tumour model when given as monotherapy and resulted in complete tumour regression in > 50% of treated mice when given in combination with chemotherapy.
- Combination therapy (dual targeting of PD-L1 and CTLA-4) resulted in tumour regression in a mouse model of colorectal cancer.
- Dual targeting of PD-1 and PD-L1 in a syngeneic model of sarcoma in mice demonstrated statistically significant mean tumour growth delay relative to the control group.

3.1.5 *Animal Toxicology*

In general, treatment of cynomolgus monkeys with durvalumab was not associated with any durvalumab-related adverse effects that were considered to be of relevance to humans.

Data from the pivotal 3-month GLP toxicity study with durvalumab in cynomolgus monkeys showed that subchronic dosing of durvalumab was not associated with any adverse effects. Therefore, the NOAEL of durvalumab in all the general toxicity studies was considered to be 100 mg/kg, the highest dose tested in these studies. In addition to the in vivo toxicology data, no unexpected membrane binding of durvalumab to human or cynomolgus monkey tissues was observed in GLP tissue cross-reactivity studies using normal human and cynomolgus monkey tissues.

3.1.6 *Clinical Trials*

As of the most recent Investigator's Brochure, over 5000 subjects have been enrolled and treated in ongoing durvalumab clinical studies No studies have been completed or terminated prematurely due to toxicity.

The safety profile of durvalumab as monotherapy and combined with other anticancer agents appears consistent with the pharmacology of the target and other agents in the immune checkpoint inhibitor class. No tumour types appeared to be associated with unique AEs. Immune-related AEs (irAEs), which are important risks of immune checkpoint inhibitors, have been observed with durvalumab and include pneumonitis, hepatitis, diarrhea/colitis and intestinal perforation, endocrinopathies (hypo- and hyperthyroidism, adrenal insufficiency, hypophysitis / hypopituitarism diabetes insipidus and Type 1 diabetes mellitus), nephritis, rash/dermatitis, pancreatitis, myocarditis, myositis/polymyositis and rare/less frequent irAEs including neuromuscular toxicities such as myasthenia gravis and Guillain-Barre syndrome. Other inflammatory responses that are rare with a potential immune-mediated etiology include, but are not limited to, pericarditis, sarcoidosis, uveitis and other events involving the eye, skin, haematological and rheumatological events. It is possible that events with an inflammatory or immune-mediated mechanism could occur in nearly all organs. Please refer to the most recent version of the Investigator Brochure for incidence.

3.1.7 *Pharmaceutical Data - Durvalumab*

Supplied:
Supplied as a vialed liquid solution containing 500 mg (nominal) durvalumab. The solution contains 50 mg/mL durvalumab, 26 mM histidine/histidine-HCI, 275 mM trehalose dihydrate, 0.02% (w/v) polysorbate 80, at pH 6.0.

Storage:
Durvalumab must be stored at 2°C to 8°C.

Route of Administration:
Intravenous.

Please refer to the CO.26 Pharmacy Manual for additional details.

3.2 Tremelimumab

3.2.1 *Name and Chemical Information*

Tremelimumab is a human monoclonal antibody of the immunoglobulin G2 (IgG2) subclass that inhibits binding of B7 ligands (B7.1 (CD80) or B7.2 (CD86)) to cytotoxic T lymphocyte-associated antigen 4 (CTLA-4; cluster of differentiation [CD]152).

See the current tremelimumab Investigator Brochure for additional details and the most up to date information.

3.2.2 *Chemical Structure*

Tremelimumab has an overall molecular weight of approximately 149 kDa including oligosaccharides.

3.2.3 *Mechanism of Action*

Tremelimumab binds with high affinity and specificity to human CTLA-4, a cell surface receptor expressed primarily on activated T cells. Binding of CTLA-4 to its target ligands (B7.1 and B7.2) on antigen-presenting cells, provides a negative regulatory signal, which limits T-cell activation. Tremelimumab blocks this interaction of B7 ligands with CTLA-4, thus leading to prolongation and enhancement of T-cell activation and expansion. This mechanism is supported by in vitro studies where tremelimumab antagonizes binding of CTLA-4 to B7 ligands and enhances human T-cell activation as demonstrated by increased cytokine (IL-2, IFN-γ) production.

3.2.4 *Experimental Antitumour Activity*

In a mouse model of fibrosarcoma, an anti-mouse CTLA-4 antibody demonstrated dose-dependent antitumour activity and, at the maximum dose tested, resulted in complete tumour regression in 4 of 5 treated animals. Also these animals were resistant to tumour rechallenge, demonstrating a durable antitumour immunity. Finding was corroborated in other mouse models of cancer.

In a mouse model of colon cancer, the combination of anti-mouse PD-L1 and anti-mouse CTLA-4 resulted in greatly increased activity with tumour regression observed in all mice treated relative to control.

3.2.5 *Clinical Trials*

To date, 34 clinical studies have been conducted in over 1500 patients in both monotherapy and combination therapy clinical trials. Full details are described in the current tremelimumab Investigator Brochure.

To date, no tumour type or stage appears to be associated with unique AEs (except for vitiligo that appears to be confined to subjects with melanoma). Treatment-related AEs were reported at similar rates in the 10 and 15 mg/kg groups, and were mostly Grade 1 or 2 in severity. The most frequent (in > 5% of subjects) treatment-related AEs (all grades) in patients with tremelimumab monotherapy were diarrhea, rash, pruritus, fatigue, nausea, vomiting, decreased appetite, headache, pyrexia, abdominal pain, and colitis. Please refer to the most recent version of the Investigator Brochure for incidence.

Across clinical trials, a pattern of efficacy has emerged that is similar to the anti-CTLA-4 antibody, ipilimumab, which appears to be consistent across tumour types. Response rates to anti-CTLA-4 antibodies are generally low, approximately 10%. However, in subjects who respond, the responses are generally durable, lasting several months even in subjects with aggressive tumours such as refractory metastatic melanoma.

3.2.6 *Pharmaceutical Data - Tremelimumab*

Supplied:
Supplied as a vialed solution containing 400 mg (nominal) tremelimumab. The solution contains 20 mg/mL tremelimumab, 20 mM histidine/histidine-HCI, 222 mM trehalose dihydrate, 0.02% (w/v) polysorbate 80, 0.27 mM disodium edetate dehydrate (EDTA), pH 5.5.

Storage:
Tremelimumab must be stored at 2°C to 8°C and must not be frozen. The product should be protected from light when not in use.

Route of Administration:
Intravenous.

Please refer to the CO.26 Pharmacy Manual for additional details.

3.3 Fixed Dosing in Durvalumab and Tremelimumab

A population PK model was developed for durvalumab using monotherapy data from a phase I study (study 1108; N=292; doses= 0.1 to 10 mg/kg Q2W or 15 mg/kg Q3W; solid tumours). Population PK analysis indicated only minor impact of body weight (WT) on PK of durvalumab (coefficient of ≤ 0.5). The impact of body WT-based (10 mg/kg Q2W) and fixed dosing (750 mg Q2W) of durvalumab was evaluated by comparing predicted steady state PK concentrations (5th, median and 95th percentiles) using the population PK model. A fixed dose of 750 mg was selected to approximate 10 mg/kg (based on median body WT of ~75 kg). A total of 1000 patients were simulated using body WT distribution of 40–120 kg. Simulation results demonstrate that body WT-based and fixed dosing regimens yield similar median steady state PK concentrations with slightly less overall between-subject variability with fixed dosing regimen.

Similarly, a population PK model was developed for tremelimumab using data from Phase 1 through Phase 3 (N=654; doses= 0.01 to 15 mg/kg Q4W or Q90D; metastatic melanoma) [Wang et al. 2014]. Population PK model indicated minor impact of body WT on PK of tremelimumab (coefficient of ≤ 0.5). The WT-based (1 mg/kg Q4W) and fixed dosing (75 mg/kg Q4W; based on median body WT of ~75 kg) regimens were compared using predicted PK concentrations (5th, median and 95th percentiles) using population PK model in a simulated population of 1000 patients with body weight distribution of 40 to 120 kg. Similar to durvalumab, simulations indicated that both body WT-based and fixed dosing regimens of tremelimumab yield similar median steady state PK concentrations with slightly less between-subject variability with fixed dosing regimen.

Similar findings have been reported by others *[Ng 2006, Wang 2009, Zhang 2012, Narwal 2013]*. Wang and colleagues investigated 12 monoclonal antibodies and found that fixed and body size-based dosing perform similarly, with fixed dosing being better for 7 of 12 antibodies. In addition, they investigated 18 therapeutic proteins and peptides and showed that fixed dosing performed better for 12 of 18 in terms of reducing the between-subject variability in pharmacokinetic / pharmacodynamics parameters *[Zhang 2012]*.

A fixed dosing approach is preferred by the prescribing community due to ease of use and reduced dosing errors. Based on average body WT of 75 kg, a fixed dose of 1500 mg Q4W and 75 mg tremelimumab (equivalent to 1 mg/kg) is planned.

Fixed dosing of durvalumab and tremelimumab is recommended only for subjects with > 30 kg body weight due to endotoxin exposure. Patients with a body weight less than or equal to 30 kg should be dosed using a weight-based dosing schedule. This is not expected to be applicable to this trial in an adult patient population.

4.0 STUDY POPULATION

The trial population will consist of patients with advanced (metastatic or locally advanced) histologically confirmed colorectal cancer that is unresectable and who have exhausted standard treatment options. Patients will have progressed with or after standard chemotherapy based regimens containing a fluoropyrimidine, irinotecan and oxaliplatin, or are deemed unsuitable for such regimens by their treating physicians. Subjects with *RAS* wild type tumours must have previously received cetuximab or panitumumab. Subjects treated previously with the following agents will be eligible, though previous treatment with these agents is not a requirement for enrolment:

- anti-VEGF therapy, such as bevacizumab and/or aflibercept and/or regorafenib
- TAS-102

This study is designed to include women and minorities as appropriate, but is not designed to measure differences in intervention effects in these patient populations.

4.1 Eligibility Criteria

There will be NO EXCEPTIONS to eligibility requirements at the time of randomization. Questions about eligibility criteria should be addressed prior to calling for randomization.

The eligibility criteria for this study have been carefully considered. Eligibility criteria are standards used to ensure that patients who enter this study are medically appropriate candidates for this therapy. For the safety of the patients, as well as to ensure that the results of this study can be useful for making treatment decisions regarding other patients with similar diseases, it is important that no exceptions be made to these criteria for admission to the study.

Patients must fulfil all of the following criteria to be eligible for admission to the study:

4.1.1 Must have histologically or pathologically confirmed advanced (metastatic or locally advanced) colorectal cancer that is unresectable.

4.1.2 Received a prior thymidylate synthase inhibitor (e.g. 5-fluorouracil (5-FU), capecitabine, raltitrexed, UFT) for metastatic disease or as adjuvant therapy. A thymidylate synthase inhibitor may have been given in combination with oxaliplatin or irinotecan.

The intention of this criterion is to ensure that it is the conclusion of the investigator that this patient would not benefit from further therapy with a thymidylate synthase inhibitor and is therefore an appropriate candidate for treatment with best supportive care only.

4.1.3 Received and failed an irinotecan -containing regimen (i.e. single-agent or in combination) for treatment of metastatic disease, OR relapsed within 6 months of completion of an irinotecan-containing adjuvant therapy, OR have documented unsuitability for an irinotecan-containing regimen.

Failure is defined as either progression of disease (clinical or radiologic) or intolerance to the irinotecan-containing regimen, where intolerance is defined as discontinuation due to any of the following: severe allergic reaction or delayed recovery from toxicity preventing retreatment.

Documented unsuitability for irinotecan includes (but is not confined to) known hypersensitivity to irinotecan, abnormal glucuronidation of bilirubin, Gilbert's syndrome or previous pelvic/abdominal irradiation.

The intention of this criterion is to ensure that it is the conclusion of the investigator that this patient would not benefit from further therapy with irinotecan and is therefore an appropriate candidate for treatment with best supportive care measures only.

4.1.4 Received and failed an oxaliplatin-containing regimen (i.e. single-agent or in combination) for treatment of metastatic disease, OR relapsed within 6 months of completion of an oxaliplatin-containing adjuvant therapy OR have documented unsuitability for an oxaliplatin-containing regimen.

Failure is defined as either progression of disease (clinical or radiological) or intolerance to the oxaliplatin-containing regimen, where intolerance is defined as discontinuation due to any of the following: severe allergic reaction, persistent severe neurotoxicity or delayed recovery from toxicity preventing retreatment.

Documented unsuitability for oxaliplatin includes (but is not confined to) known hypersensitivity to oxaliplatin or other platinum compounds, pre-existing renal impairment, or Grade 2 or greater neurosensory neuropathy.

The intention of this criterion is to ensure that it is the conclusion of the investigator that this patient would not benefit from further therapy with oxaliplatin and is therefore an appropriate candidate for treatment with best supportive care measures only.

4.1.5 *For patients with colorectal cancer that is RAS-wild type:*
Received and failed a cetuximab or panitumumab-containing regimen (i.e. single-agent or in combination) for treatment of metastatic disease OR have documented unsuitability for a cetuximab or panitumumab-containing regimen

Failure is defined as either progression of disease (clinical or radiological) or intolerance to the cetuximab- or panitumumab-containing regimen, where intolerance is defined as discontinuation due to any of the following: severe infusion reaction, persistent severe skin toxicity or delayed recovery from toxicity preventing retreatment.

Documented unsuitability for cetuximab includes (but is not confined to) known hypersensitivity to cetuximab or the presence of tumours with an activating *RAS or RAF* mutation.

Documented unsuitability for panitumumab includes (but is not confined to) known hypersensitivity to panitumumab or the presence of tumours with an activating *RAS* mutation.

The intention of this criterion is to ensure that it is the conclusion of the investigator that this patient would not benefit from further therapy with either cetuximab or panitumumab and is therefore an appropriate candidate for treatment with best supportive care measures only.

4.1.6 Patient prior treatment with VEGF targeting therapy, such as bevacizumab, aflibercept, ramucirumab, or regorafenib, is permitted but not mandatory. Reasons not used are to be documented.

4.1.7 Patient prior treatment with TAS-102 (an agent composed of a combination of trifluorothymidine (FTD) and tipiracil hydrochloride (TPI)), is permitted but not mandatory. Reasons not used are to be documented.

4.1.8 The only remaining standard available therapy as recommended by the Investigator, in consultation with the patient, is best supportive care.

4.1.9 Must have presence of measurable or evaluable disease as defined by Response Evaluation Criteria in Solid Tumours (RECIST 1.1).

4.1.10 Imaging investigations including CT/MRI of chest/abdomen/pelvis or other scans as necessary to document all sites of disease done within 28 days prior to randomization.

4.1.11 Must have an Eastern Cooperative Oncology Group (ECOG) performance status of 0 or 1.

4.1.12 Life expectancy of ≥ 12 weeks at the time of study entry.

4.1.13 Must be ≥ 18 years of age. (Note that the lower age limit at each centre will be determined by that centre’s policy regarding the age at which an individual may sign their own consent.)

4.1.14 Women/men of childbearing potential must have agreed to use a highly effective contraceptive method. A woman is considered to be of "childbearing potential" if she has had menses at any time in the preceding 12 consecutive months. In addition to routine contraceptive methods, "effective contraception" also includes heterosexual celibacy and surgery intended to prevent pregnancy (or with a side-effect of pregnancy prevention) defined as a hysterectomy, bilateral oophorectomy or bilateral tubal ligation, or vasectomy/vasectomized partner. However, if at any point a previously celibate patient chooses to become heterosexually active during the time period for use of contraceptive measures outlined in the protocol, he/she is responsible for beginning contraceptive measures.

Female patients of childbearing potential who are sexually active with a non-sterilized male partner must use at least one highly effective method of contraception while on study and for 6 months after the last dose of durvalumab and tremelimumab or for 3 months after the last dose of durvalumab alone. Male partners of a female subject and non-sterilized male patients who are sexually active with a female partner of childbearing potential must use male condom plus spermicide while on study and for 6 months after the last dose of durvalumab and tremelimumab or for 3 months after the last dose of durvalumab alone. Female partners of a male subject must use a highly effective method of contraception throughout this period. Cessation of birth control after this point should be discussed with a responsible physician. See Section 9.3.1 for additional details.

Women of childbearing potential will have a pregnancy test to determine eligibility as part of the Pre-Study Evaluation (see Sections 5.1 and 5.2); this may include an ultrasound to rule-out pregnancy if a false-positive is suspected. For example, when beta-human chorionic gonadotropin is high and partner is vasectomized, it may be associated with tumour production of hCG, as seen with some cancers. Patient will be considered eligible if an ultrasound is negative for pregnancy.

Male patients should also refrain from donating sperm during the study and for 6 months after the last dose of durvalumab and tremelimumab or for 3 months after the last dose of durvalumab alone.

4.1.15 Patient must consent to provision of, and investigator(s) must confirm adequacy of tissue, and confirm access to and agree to submit within 4 weeks of randomization to the CCTG Central Tumour Bank, a representative formalin fixed paraffin block of tumour tissue in order that the specific correlative marker assays proscribed in Section 12 (Correlative Studies) may be conducted. Where adequate amount and quality of tissue exists but local centre regulations prohibit submission of blocks of tumour tissue, the approval of the CCTG must be sought prior to randomization of the first patient to allow cores (two 2 mm cores of tumour from the block) and a predetermined number of slides of representative tumour tissue to be substituted. Failure to submit any tissue samples on request will result in the patient being considered ineligible. Where no previously resected or biopsied tumour tissue exists or is found to be of inadequate amount or quality, additional biopsy of the primary or metastatic tumour will be required for the patient to be considered eligible for the study and will be required to be done prior to randomization. Please refer to the CO.26 Correlative Studies Manual for details concerning adequacy of amount and quality of tumour tissue.

4.1.16 Patient must consent to provision of samples of blood in order that the specific correlative marker assays proscribed in Section 12 (Correlative Studies) may be conducted.

4.1.17 Patient is able (i.e. sufficiently fluent) and willing to complete the quality of life questionnaires in either English or French. The baseline assessment must be completed within 14 days prior to randomization. Inability (lack of comprehension in English or French, or other equivalent reason such as cognitive issues or lack of competency) to complete the questionnaires will not make the patient ineligible for the study. However, ability but unwillingness to complete the questionnaires will make the patient ineligible.

4.1.18 Patients must be accessible for treatment and follow-up. Investigators must assure themselves the patients randomized on this trial will be available for complete documentation of the treatment, adverse events, and follow-up.

4.1.19 In accordance with CCTG policy, protocol treatment is to begin within 2 working days of patient randomization.

4.1.20 The patient is not receiving therapy in a concurrent clinical study and the patient agrees not to participate in other clinical studies during their participation in this trial while on study treatment.

4.1.21 Adequate normal organ and marrow function as defined below (must be done within 14 days prior to randomization):

Hematology	Absolute neutrophils	≥ 1.5 x 10^9/L
	Platelets	≥ 100 x 10^9/L
	Hemoglobin	≥ 90 g/L
	Lymphocytes	*Note: no range is specified for eligibility*
Chemistry	Bilirubin	≤ 1.5 x ULN (upper limit of normal)*
	AST and ALT **	≤ 2.5 x ULN
	Serum creatinine<br>*or:*<br>Creatinine clearance***	< 1.25 x ULN<br><br>≥ 40 mL/min

* If confirmed Gilbert's, eligible providing ≤ 3 x ULN.

** < 3 x UNL in presence of liver metastases

*** Creatinine clearance to be measured directly by 24 hour urine sampling or as calculated by Cockcroft and Gault equation below:

Females: GFR = 1.04 x (140-age) x weight in kg / serum creatinine in μmol/L

Males: GFR = 1.23 x (140-age) x weight in kg / serum creatinine in μmol/L

4.1.22 Patient consent must be appropriately obtained in accordance with applicable local and regulatory requirements. Each patient must sign a consent form prior to enrolment in the trial to document their willingness to participate.

4.2 Ineligibility Criteria

Patients who fulfill any of the following criteria are not eligible for admission to the study:

4.2.1 Patients with a history of other malignancies, except: adequately treated non-melanoma skin cancer, curatively treated in-situ cancer of the cervix, or other solid tumours curatively treated with no evidence of disease for ≥ 5 years.

4.2.2 Any previous treatment with a PD1 or PD-L1 inhibitor, including durvalumab, or an anti-CTLA4, including tremelimumab.

4.2.3 History of primary immunodeficiency, history of organ transplant that requires therapeutic immunosuppression or prior history of severe (grade 3 or 4) immune mediated toxicity from other immune therapy.

4.2.4 Current or prior use of immunosuppressive medication within 28 days before the first dose of durvalumab or tremelimumab, with the exceptions of intranasal and inhaled corticosteroids or systemic corticosteroids at physiological doses, which are not to exceed 10 mg/day of prednisone, or an equivalent corticosteroid.

4.2.5 Active or prior documented autoimmune or inflammatory disorders (including inflammatory bowel disease (e.g. Crohn's disease, ulcerative colitis) within the past 2 years NOTE: Subjects with vitiligo, Grave's disease, or psoriasis not requiring systemic treatment (within the past 2 years) are not excluded.

4.2.6 Patients with active or uncontrolled intercurrent illness including, but not limited to:

- cardiac dysfunction (symptomatic congestive heart failure, uncontrolled hypertension, unstable angina pectoris, cardiac arrhythmia)
- active peptic ulcer disease or gastritis,
- active bleeding diatheses
- psychiatric illness/social situations that would limit compliance with study requirements or compromise the ability of the subject to give written informed consent
- known history of previous clinical diagnosis of tuberculosis
- known human immunodeficiency virus infection (positive HIV 1/2 antibodies)
- known active hepatitis B infection (positive HBV surface antigen (HBsAg). Patients with a past or resolved HBV infection (defined as presence of hepatitis B core antibody (anti-HBc) and absence of HBsAg) are eligible.
- known active hepatitis C infection. Patients positive for hepatitis C (HCV) antibody are eligible only if polymerase chain reaction is negative for HCV RNA.

4.2.7 History of leptomeningeal carcinomatosis.

4.2.8 Symptomatic or uncontrolled brain metastases requiring concurrent treatment, inclusive of but not limited to surgery, radiation and/or corticosteroids.

4.2.9 Receipt of live attenuated vaccination (examples include, but are not limited to, vaccines for measles, mumps, and rubella, live attenuated influenza vaccine (nasal), chicken pox vaccine, oral polio vaccine, rotavirus vaccine, yellow fever vaccine, BCG vaccine, typhoid vaccine and typhus vaccine) within 30 days prior to randomization.

4.2.10 Pregnant or lactating women.

4.2.11 Any active disease condition which would render the protocol treatment dangerous or impair the ability of the patient to receive protocol therapy.

4.2.12 Any condition (e.g. psychological, geographical, etc.) that does not permit compliance with the protocol.

4.2.13 Receipt of anti-cancer chemotherapy or biologic therapy within the lesser of i) 21 days, or ii) the usual cycle length of the regimen (e.g. 14 days for FOLFOX), prior to the first planned dose of durvalumab or tremelimumab. An exception is made for capecitabine and regorafenib, where a minimum of 10 days since last dose must be observed prior to the first planned dose of durvalumab or tremelimumab.

4.2.14 Receipt of radiotherapy or investigational agents within four weeks of first planned dose of durvalumab or tremelimumab, with the exception of a single dose of radiation up to 8 Gray (equal to 800 RAD) with palliative intent for pain control up to 14 days before randomization.

4.2.15 Any unresolved toxicity (CTCAE grade 2) from previous anti-cancer therapy. However, patients with irreversible toxicity that is not reasonably expected to be exacerbated by the investigational products in the Investigator’s opinion may be included (e.g. hearing loss, peripheral neuropathy).

5.0 PATIENT EVALUATION FLOWSHEET: PRE-TREATMENT, ON STUDY, AND AFTER TREATMENT

All patients entered on study must be evaluated according to the schedule outlined below with documentation submitted according to the schedule in Appendix IV.

5.1 Patient Evaluation Flowsheet: Pre-study (Baseline) and Post-randomization Follow-up

For Patients Randomized to Best Supportive Care (Arm 1)

Required Investigations	PRE-STUDY (≤ 14 days prior to randomization, unless otherwise indicated)	PRIOR to objective disease progression (timing from randomization)		AFTER objective disease progression (timing from randomization)
		Every 4 weeks until objective disease progression	Every 8 weeks until objective disease progression (maintain schedule even if clinic visits are delayed)[3]	Every 12 weeks
**History and Physical Exam**				
Including: height (baseline only), weight, ECOG performance status, clinical tumour measurements (if applicable)	X	X		
Vital Signs (blood pressure, heart rate, temperature)	X	X		
Concomitant Medications	X	X		
**Overall Survival**				
Survival Status Assessment		X	X	X[1]
**Hematology**				
CBC, differential (including lymphocytes), platelets	X	X		
**Coagulation**				
PTT, PT/INR	X	X		
**Biochemistry**				
Serum Creatinine, Chloride, Sodium, Potassium, Calcium, Magnesium, Bilirubin, ALP, AST and ALT, LDH, Albumin, TSH[2], Amylase, Lipase, Serum CEA	X	X		
Creatinine Clearance (calculated)	X	as clinically indicated		
Glucose	X	as clinically indicated		
**Radiology[3]**				
Chest/abdomen/pelvis CT or MRI scan[3]	X ≤ 28 days		X[3]	
Other scans as necessary to document all measurable and non-measurable disease[3]	X ≤ 28 days			
**Other Investigations**				
Pregnancy Test[4]	X			
ECG	X	as clinically indicated		
Dipstick Urinalysis (including protein, specific gravity, glucose and blood)	X			

table continued on next page ...

Correlative Studies				
Archival Tissue Sample[5] (mandatory)	Availability of sufficient tissue must be confirmed prior to randomization	Submit tissue within 4 weeks of randomization		
Whole blood, plasma and serum[5,6] (mandatory)	X	To be done at 8 weeks, and at the time of objective disease progression[7], if not already done		
Adverse Events				
Adverse Event Assessment[8]	X (To document residual adverse events form previous therapy and baseline symptoms)	X To be evaluated continuously for adverse events (until objective disease progression)		
Quality of Life				
EORTC QLQ C30[9]	X	At 4, 8, 12, 16 and 24 weeks after randomization, then every 12 weeks thereafter (until deterioration to ECOG PS 4 or hospitalization for end of life care)		

1. In the event that patient is unable to attend clinic, post-progression follow-up may be by means of telephone contact.
2. If abnormal, T3 and T4 must be measured.
3. To ensure comparability, scans must identify and report each lesion at baseline and at reassessment. These scans must be performed using identical technique (i.e. scans performed immediately following bolus contrast administration using a standard volume of contrast, the identical contrast agent, and preferably the same scanner). Tumour evaluations must be consistently performed every 8 weeks (i.e. at 8 weeks, 16 weeks, 24 weeks, 32 weeks etc. from randomization) until objective disease progression is documented (as described in Section 8). Sites should adhere to this calendar-based schedule regardless of any delays in clinic visits . If a radiology scan is done off schedule, future protocol-required scans should still be performed based on the original schedule, i.e. every 8 weeks counting from randomization (not from the date of the off schedule scan).
4. For women of childbearing potential only. May be urine or serum. Pregnancy test (in women of childbearing potential), as part of Pre-Study Evaluation, may include an ultrasound to rule-out pregnancy.
5. See Section 12.0 and the CO.26 Correlative Studies Laboratory Manual for details.
6. Details for collection, processing, storing, and shipping these samples will be provided in the CO.26 Correlative Studies Manual
7. Whole blood, plasma and serum should be obtained as close to the time of objective progression as possible and should be done within 28 days (e.g. at the next clinic visit). Blood for correlatives done within 28 days PRIOR to date of progression does not need to be repeated.
8. Adverse Events to be evaluated using the NCI Common Terminology Criteria for Adverse Events (CTCAE v4.0) (see Appendix V).
9. To be completed by patient in clinic.

5.2 Patient Evaluation Flowsheet: Pre-Treatment, On-Study, and after Protocol Treatment

For Patients Randomized to Durvalumab and Tremelimumab (Arm 2)

Required Investigations	PRE-STUDY (≤14 days prior to randomization, unless otherwise indicated)	DURING Protocol Treatment	AFTER Protocol Treatment is Permanently Discontinued[1]			
			PRIOR to objective disease progression		AFTER objective disease progression	
		Day 1 each cycle, and as clinically indicated (unless otherwise indicated)	4 weeks after end of last cycle date	Every 4 weeks thereafter	4 weeks after end of last cycle date[1]	Every 12 weeks thereafter
**History and Physical Exam[2]**						
Including: height (baseline only), weight, ECOG performance status, clinical tumour measurements (if applicable)	X	X	X	X	X	
Vital Signs (blood pressure, heart rate, temperature)	X	X[3]				
Concomitant Medications	X	X	X		X	
**Overall Survival**						
Survival status assessment		X	X	X	X	X[4]
**Hematology[2,5]**						
CBC, differential (including lymphocytes), platelets	X	X[2]	X	X	X	X[5]
**Coagulation[2,5]**						
PTT, PT/INR	X	X[2]	X	X	X	X[5]
**Biochemistry[2,5]**						
Serum Creatinine, Chloride, Sodium, Potassium, Calcium, Magnesium, Bilirubin, ALP, AST and ALT, LDH, Albumin, TSH[6], Amylase, Lipase, Serum CEA	X	X[2]	X	X	X	X[5]
Creatinine Clearance (calculated)	X	as clinically indicated				
Glucose	X	as clinically indicated				
**Radiology[7]**						
Chest/abdomen/pelvis CT or MRI scan	X ≤ 28 days	X[7] Every 8 weeks from randomization until objective disease progression (maintain schedule even if cycles are delayed)				
Other scans as necessary to document all measurable and non-measurable disease	X ≤ 28 days					
**Other Investigations**						
Pregnancy Test[8]	X					
ECG	X	as clinically indicated				
Dipstick Urinalysis (including protein, specific gravity, glucose and blood)	X					

table continued on next page ...

Required Investigations	PRE-STUDY (≤14 days prior to randomization, unless otherwise indicated)	DURING Protocol Treatment	AFTER Protocol Treatment is Permanently Discontinued[1]			
			PRIOR to objective disease progression		AFTER objective disease progression	
		Day 1 each cycle, and as clinically indicated (unless otherwise indicated)	4 weeks after end of last cycle date	Every 4 weeks thereafter	4 weeks after end of last cycle date[1]	Every 12 weeks thereafter
**Correlative Studies**						
Archival Tissue Sample[9] (mandatory)	Availability of sufficient tissue must be confirmed prior to randomization	Submit tissue within 4 weeks of randomization				
Whole blood, plasma and serum[9] (mandatory)	After randomization but before first dose of study treatment	To be done at 8 weeks and at the time of objective disease progression[10], if not already done				
**Adverse Events**						
Adverse Event Assessment[11]	X (To document residual adverse events form previous therapy and baseline symptoms)	X[12] (To be evaluated continuously for adverse events)	X	X[13]	X[1]	X[13]
**Quality of Life**						
EORTC QLQ C30[14]	X	At 4, 8, 12, 16 and 24 weeks after randomization, then every 12 weeks thereafter (until deterioration to ECOG PS 4 or hospitalization for end of life care)				

1. All patients will be seen at 4 weeks after the end of the last cycle date. Thereafter, continued follow-up is not required for patients who go off protocol treatment with unequivocal progressive disease, except to document ongoing toxicities (until resolved to < grade 2) and late toxicities (including second malignancies). For patients who go off protocol treatment with CR, PR, or SD ongoing, follow-up will be required every 12 weeks until relapse (see Appendix I for investigations to be performed). Death Report will be required for all patients. Due within 2 weeks of knowledge of death (see Appendix IV - Documentation for Study).
2 Timing of Day 1 Assessments: Pre-treatment blood draws and physical exams may be done one working day prior to treatment if necessary , and when treatment is to begin on a Monday, may be done on the previous Friday (maximum 72 hours prior to treatment). In order to ensure that nadir counts are not missed, every effort should be made to do interim blood draws within 24 hours of the day specified in the protocol. If a patient shows an AST or ALT ≥ 3 x ULN together with total bilirubin ≥ 2 x ULN, refer to Appendix III for further instructions on cases of increases in liver biochemistry and evaluation of Hy's Law. These cases should be reported as SAEs if, after evaluation, they meet the criteria for a Hy's law case or if any of the individual liver test parameters fulfill any of the SAE criteria. Note: Labs do not need to be repeated Day 1, Cycle 1. In order to ensure that nadir counts are not missed, every effort should be made to do interim blood draws within 24 hours prior to the day specified in the protocol.
3. Vital signs are to be monitored before, during, and after the infusion of tremelimumab and durvalumab with assessment of vital signs to be collected ≤ 30 minutes prior to start of infusion then every 30 ±5 minutes during infusion and observation periods. A 1-hour observation period is recommended after the first infusion of tremelimumab, during which time durvalumab may be administered (see Section 7.1.3). A second 1-hour observation period is recommended after the first infusion of durvalumab. If no clinically significant infusion reactions are observed during or after the first cycle of tremelimumab and durvalumab, subsequent infusion observation periods can be at the Investigator's discretion but are recommended to be of 30 minutes, i.e., 30 minutes after tremelimumab infusion, during durvalumab infusion, and for 30 minutes after durvalumab infusion. For patients who receive only durvalumab, the recommended observation period is 30 minutes after infusion.
4. In the event that patient is unable to attend clinic, post-progression follow-up may be by means of telephone contact.
5. Hematology, Coagulation and Biochemistry investigation to be done after objective progression ONLY if there are ongoing hematologic AEs that are related to protocol treatment.
6. If abnormal, T3 and T4 must be measured.
7. To ensure comparability, scans must identify and report each lesion at baseline and at reassessment during treatment. These scans must be performed using identical technique (i.e. scans performed immediately following bolus contrast administration using a standard volume of contrast, the identical contrast agent, and preferably the same scanner). Tumour evaluations must be consistently performed every 8 weeks (i.e. at 8 weeks, 16 weeks, 24 weeks, 32 weeks etc. from randomization) until objective disease progression is documented (as described in Section 8). Sites should adhere to this calendar-based schedule regardless of any delays in treatment. If a radiology scan is done off schedule, future protocol-required scans should still be performed based on the original schedule, i.e. every 8 weeks counting from randomization (not from the date of the off schedule scan).
8. For women of childbearing potential only. May be urine or serum. Pregnancy test (in women of childbearing potential), as part of Pre-Study Evaluation, may include an ultrasound to rule-out pregnancy.
9. See Section 12.0 and the CO.26 Correlative Studies Laboratory Manual for details.
10. Whole blood, plasma and serum should be obtained as close to the time of objective progression as possible and should be done within 28 days (e.g. at the next clinic visit). Blood for correlatives done within 28 days PRIOR to date of progression does not need to be repeated.
11. Adverse Events to be evaluated using the NCI Common Terminology Criteria for Adverse Events (CTCAE v4.0) (see Appendix V).
12. See Section 7 and Appendix III for additional monitoring of patients with toxicity.
13. Follow every adverse event felt related to study therapy until resolved to ≤ grade 2 – i.e. follow-up every 12 weeks until deterioration to ECOG PS 4 and/or end of life care or until all toxicities thought to be possibly-, probably-, or definitely-related to durvalumab or tremelimumab have resolved, returned to baseline, or been deemed irreversible, whichever is longer.
14. To be completed by patient in clinic.

5.3 Follow-up for Ineligible Patients

The follow-up requirements for ineligible patients:

- Randomized to **Arm 1** (BSC) include submission of the Baseline Report plus annual follow-up using the Minimal Follow-up Report.
- Randomized to **Arm 2** (tremelimumab and durvalumab) but who have received no protocol therapy include submission of the Baseline Report plus annual follow-up using the Minimal Follow-up Report. Data submission for ineligible participants who have received at least one dose of protocol therapy should be followed according to the protocol to allow for treatment and adverse event assessment.

Note: The dates of objective progression and death must be reported for ineligible patients.

6.0 ENTRY/RANDOMIZATION PROCEDURES

6.1 Entry Procedures

All registration/randomizations will be done through the CCTG web-based, password-protected Electronic Data Capture (EDC) system. Complete details regarding obtaining a password, accessing the system and registering/randomizing patients will be provided at the time of study activation and will also be included in the "EDC Data Management Guidebook", posted on the CO.26 trial specific website. If sites experience difficulties accessing the system and/or registering/randomizing patients please contact the help desk (link in EDC) or the CO.26 Study Coordinator.

All eligible patients enrolled on the study by each participating centre will be assigned a serial number which must be used on all documentation and correspondence with CCTG.

The following information will be required:

- trial code (CCTG CO.26)
- investigator CCTG user ID
- patient's initials (may be coded)
- informed consent version date, date signed by patient, name of person conducting consent discussion and date signed
- tissue banking consent version date
- confirmation of the requirements listed in Section 4.0, including dates of essential tests and actual laboratory values
- stratification factors

6.2 Stratification

Subjects will be stratified by:

- ECOG Performance Status: 0 vs1
- Site of tumour:
  - right colon (caecum, ascending colon, hepatic flexure) *vs*
  - transverse colon *vs*
  - left colon (within the splenic flexure, descending colon, sigmoid colon, or rectosigmoid junction) *vs*
  - rectum *vs*
  - unknown

6.3 Randomization

Randomization will be provided electronically.

Note: The validity of results of the trial depends on the authenticity of and the follow-up of all patients entered into the trial. Under no circumstances, therefore, may an allocated patient's data be withdrawn prior to final analysis, unless the patient withdraws from the trial and requests that data collection/submission cease from the point in time of withdrawal.

All eligible patients admitted to the trial will be followed by the coordinating centre. It is the responsibility of the treating physician to satisfy himself or herself that the patient is indeed eligible before requesting registration/randomization.

All randomized patients are to be followed until death or until sites are informed by CCTG that further follow-up is no longer required. The follow-up requirements for ineligible patients are outlined in Section 4.2.

7.0 TREATMENT PLAN

Although the Canadian Cancer Clinical Trials Group acts as the coordinating agency for the trial, the responsibility for the treatment of patients rests with each individual investigator.

In accordance with CCTG policy, protocol treatment is to begin within 2 working days of patient randomization.

7.1 Protocol Treatment Plan

7.1.1 *Drug Administration*

Patients will be randomized at a 2:1 ratio to receive durvalumab plus tremelimumab plus best supportive care or best supportive care only to a planned sample size of 180.

Best supportive care is defined as those measures designed to provide palliation of symptoms and improve quality of life as much as possible.

When durvalumab is given with tremelimumab, tremelimumab will be administered first. Durvalumab will start immediately after the tremelimumab infusion (during the post-tremelimumab observation period).

One cycle will be defined as 28 days (4 weeks). Study drugs will be started on cycle 1. Tremelimumab will only be given for the first 4 cycles.

Arm	Agent(s)	Dose	Route	Duration* (infusion)	Schedule
1	Best Supportive Care	NA	NA	NA	NA
2	Tremelimumab	75 mg	IV	60 minutes	Day 1, cycles 1-4
	Durvalumab	1500 mg	IV	60 minutes	Day 1 every 28 days
	Best Supportive Care	NA	NA	NA	NA

* *Duration (overall) of durvalumab and tremelimumab is until unequivocal progression or unacceptable toxicity.*

Arm 2: DURVALUMAB and TREMELIMUMAB

The major toxic effects of durvalumab or tremelimumab which are anticipated to limit dosing are hypersensitivity/ infusion related reactions and possible class related immune related AEs, based on the mechanism of action of durvalumab ± tremelimumab leading to T-cell activation and proliferation. Potential immune related AEs include pneumonitis, hepatitis, diarrhea/colitis and intestinal perforation, endocrinopathies (hypo and hyperthyroidism, adrenal insufficiency, hypophysitis/hypopituitarism diabetes insipidus and Type 1 diabetes mellitus), nephritis, rash/dermatitis, pancreatitis, myocarditis, myositis/polymyositis and rare/less frequent irAEs including neuromuscular toxicities such as myasthenia gravis and Guillain-Barre syndrome. Other inflammatory responses that are rare with a potential immune-mediated etiology include, but are not limited to, pericarditis, sarcoidosis, uveitis and other events involving the eye, skin, hematological and rheumatological events. It is possible that events with an inflammatory or immune mediated mechanism could occur in nearly all organs. With anti-PD-L1 and anti-CTLA-4 combination therapy, the occurrence of overlapping or increasing cumulative toxicities that include immune related AEs could potentially occur at higher frequencies than with either durvalumab or tremelimumab monotherapy.

7.1.2 *Premedication (Arm 2)*

No routine premedication (e.g. for nausea) or prophylaxis for hypersensitivity is required. Management of symptoms should take place as necessary. Premedication is not expected to be required. See Appendix III with respect to premedication of patients that have had a prior < Grade 2 infusion-related reaction. Details of any premedication or concomitant medication given to manage or prevent adverse events should be recorded on the electronic case report form (eCRF).

7.1.3 *Patient Monitoring (Arm 2)*

Patients will be monitored before, during infusion and after the infusion of tremelimumab and durvalumab with assessment of vital signs as specified in Section 5.2. A 1-hour observation period is recommended after the first infusion of tremelimumab, at which time durvalumab may be administered. A second 1-hour observation period is recommended after the first infusion of durvalumab. After the first cycle of tremelimumab and durvalumab therapy, subsequent observation periods can be at the discretion of the investigator (30 minutes is suggested – i.e. 30 minutes after tremelimumab infusion, during durvalumab infusion, and for 30 minutes after durvalumab infusion). For patients who receive only durvalumab, the recommended observation period is 30 minutes after infusion.

Patients should be monitored for signs and symptoms of immune related AEs. In the absence of an alternate etiology (e.g. infection or relapse), signs or symptoms of events with a potential inflammatory or immune-mediate mechanism should be considered to be immune- related.

Drug Administration and Patient Monitoring/Vitals for Arm 2
(Durvalumab + Tremelimumab + BSC)

Drug administration	Infusion duration	Vital signs and Monitoring*	
Tremelimumab	60 min	Vital signs ≤ 30 minutes prior to start of infusion then every 30 ±5 minutes during infusion and observation periods	60 min observation period after administration of 1st cycle of durvalumab/tremelimumab, for subsequent cycles 30 min (recommended)
Durvalumab	60 min		
* *Guidelines for management of infusion-related reaction are summarized in 7.1.6 and Appendix III.*			

**Tremelimumab infusion**	Tremelimumab observation*	
	**Durvalumab infusion**	Durvalumab observation*
<------------- 60 minutes ------------->	*<------------- 60 minutes ------------->*	*<------------- 60 minutes ------------->*

* 60 minutes observation period (patient monitoring) after administration of 1st cycle of durvalumab/tremelimumab. For subsequent cycles 30 minutes (recommended).

Note: Vital signs ≤ 30 minutes prior to start of each infusion then every 30 ±5 minutes during infusion and observation periods.

7.1.4 *Dose Modifications*

Guidelines for dose modification and toxicity management of immune related and non-immune related adverse events are summarized in Appendix III.

The guidelines outline dose adjustments and recommended interventions for several of these immune related adverse events. If a patient experiences several adverse events and there are conflicting recommendations, please use the recommended dose adjustment that requires the greatest dose hold or discontinuation. Adverse events will be graded using the NCI Common Terminology Criteria for Adverse Events (CTCAE) (see Appendix V).

Important: If the infusion cannot be administered, it should be omitted until the next planned infusion.

Centres must contact CCTG in the event of severe immune related adverse event(s), especially when the use of drugs such as infliximab is considered.

7.1.5 Management of Toxicity

The following general guidance should be followed for management of toxicities:

1. Treat each of the toxicities with maximum supportive care (including slowing / interrupting / omitting the agent suspected of causing the toxicity where required).
2. If the symptoms promptly resolve with supportive care, consideration should be given to continuing the same doses of durvalumab and tremelimumab along with appropriate continuing supportive care.

In addition to the criteria for permanent discontinuation of study drug/regimen based on CTCAE grade/severity (tables below), permanently discontinue study drugs for the following conditions:

- Inability to reduce corticosteroid to a dose of ≤ 10 mg of oral prednisone per day (or equivalent) within 12 weeks after last doses of study drug(s).
- Recurrence of a previously experienced Grade 3 treatment-related AE following resumption of dosing.

In addition to the dose adjustments shown in Appendix III, the following are recommended:

- Patient evaluation to identify any alternative etiology.
- In the absence of a clear alternative etiology, all events of an inflammatory nature should be considered to be immune-related.
- Symptomatic and topical therapy should be considered for low-grade events.
- For persistent (greater than 3 to 5 days) low-grade (Grade 2) or severe (≥ Grade 3) events promptly start prednisone PO 1-2 mg/kg/day or IV equivalent.
- If symptoms recur or worsen during corticosteroid tapering (≥ 4 weeks of taper), increase the corticosteroid dose (prednisone dose [e.g. up to 2-4 mg/kg/day or IV equivalent]) until stabilization or improvement of symptoms, then resume corticosteroid tapering at a slower rate.
- More potent immunosuppressives (refer to individual sections of Appendix III - immune related adverse events for specific type of immunosuppressive) should be considered for events not responding to systemic steroids.
- Discontinuation of study drugs is not mandated for Grade 3 / Grade 4 inflammatory reactions attributed to local tumour response (e.g. inflammatory reaction at sites of metastatic disease, lymph nodes etc.). Continuation of study drugs in this situation should be based upon a benefit/risk analysis for that patient and be discussed with CCTG.

7.1.6 <u>*Management of Infusion Reactions*</u>

Guidelines for management of infusion-related reaction are summarized in Appendix III.

The standard infusion times for both durvalumab and tremelimumab are 1 hour. However, if there are interruptions during infusion, the total allowed time should not exceed 8 hours at room temperature.

Study personnel must be trained to recognize and treat anaphylaxis. The study site must have immediate access to appropriate drugs and medical equipment to treat acute anaphylactic reactions, emergency resuscitation teams and equipment in addition to the ability to admit patients to an intensive care unit if necessary.

7.1.7 *Duration of Therapy*

In the absence of unequivocal disease progression, as defined in Section 10.2.5, intolerable toxicity or withdrawal of consent to further participation, patients may continue to receive study treatment of durvalumab and tremelimumab at the discretion of the Investigator.

If, in the opinion of the Investigator, a patient is deriving clinical benefit from study therapy despite the documentation of objective disease progression per RECIST 1.1 criteria, then provided the patient does not have any significant, unacceptable, or irreversible toxicities that indicate that continuing treatment will not further benefit the patient, the patient may remain on study therapy pending their next disease assessment. However, appropriate imaging studies must be repeated within 4-8 weeks to re-assess treatment response. A patient with objective disease progression confirmed on subsequent assessment by either RECIST 1.1 criteria or Immune-Related Response criteria (irRC) as appropriate should be discontinued from the study. Patients should also be discontinued from the study if objective disease progression occurs in a target lesion that has previously shown a confirmed response.

For a complete list of general criteria for stopping study treatment, please see Section 10.0.

7.1.8 *Patient Compliance*

Treatment compliance will be monitored by drug accountability, as well as recording drug administration in the patient's medical record and case report form (CRF).

Arm 1: Patients randomized to ARM 1 will receive best supportive care per institutional standards at each participating centre.

7.2 Concomitant Therapy

The Investigator must be informed as soon as possible about any medication taken from the time of screening until the end of the clinical phase of the study (final study visit). Any concomitant medication(s), including herbal preparations, taken during the study are to be recorded in the eCRF.

7.2.1 *Permitted*

Investigators may prescribe concomitant medications or treatments deemed necessary to provide adequate prophylactic or supportive care except for those medications identified as "not permitted" below. In addition, the following medications or treatments are permitted during the study:

- Growth factors may be used according to centre policy to treat life threatening toxicity but cannot be used in place of protocol defined dose adjustments. Please consult CCTG in the case of patients experiencing multiple delays as exceptions may be made for patients who are benefitting from protocol therapy.
- Other best supportive and palliative care (e.g. pain control) as required throughout the study.
- Anti-emetics or anti-diarrheal agents as required.

7.2.2 *Not Permitted*

- Cytokines
- Other anti-cancer treatment (administration of any other anti-cancer therapy is not permitted while the patient is receiving protocol therapy. Thereafter, patients may be treated at the investigator's discretion
- Other investigational therapy
- Concurrent radiation treatment; Note: local radiation treatment of isolated lesions for palliative intent is acceptable. Protocol therapy should be held prior to and during the radiation (consult CCTG in these cases).
- Corticosteroids IV or PO (except for the treatment of ≥ Grade 3 infusion reaction and nausea prophylaxis for chemotherapy). Note: Topical applications (e.g. rash), inhaled sprays (e.g. obstructive airways diseases), eye drops or local injections (e.g. intra-articular) are allowed, as are oral dose of steroids equivalent to 10 mg or less of prednisone.
- Other immunosuppressive medications including methotrexate, azathioprine, and TNF-α blockers. Use of immunosuppressive medications for the management of investigational product-related AEs is acceptable.
- Live attenuated vaccines (examples include, but are not limited to, vaccines for measles, mumps, and rubella, live attenuated influenza vaccine (nasal), chicken pox vaccine, oral polio vaccine, rotavirus vaccine, yellow fever vaccine, BCG vaccine, typhoid vaccine and typhus vaccine) within 30 days of durvalumab and tremelimumab dosing (i.e., 30 days prior to the first dose, during treatment with durvalumab and tremelimumab and for 30 days post discontinuation of durvalumab and tremelimumab). Inactivated vaccines, such as the injectable influenza vaccine, are permitted.

8.0 CRITERIA FOR MEASUREMENT OF STUDY ENDPOINTS

8.1 Definitions

8.1.1 *Evaluable for Adverse Events*

All patients will be evaluable for adverse event evaluation from the time of their first treatment.

8.1.2 Evaluable for Response

All patients who have received at least one cycle of therapy and have their disease re-evaluated will be considered evaluable for response (exceptions will be those who exhibit objective disease progression prior to the end of cycle 1 who will also be considered evaluable). Patients on therapy for at least this period and who meet the other listed criteria will have their response classified according to the definitions set out below *[Eisenhauer 2009]*.

8.1.3 *Evaluable for Quality of Life Assessment*

All patients who have completed the quality of life questionnaire are evaluable for quality of life.

8.2 Response and Evaluation Endpoints

Response and progression will be evaluated in this study using the revised international criteria (1.1) proposed by the RECIST (Response Evaluation Criteria in Solid Tumours) committee as well as the Immune-Related Response Criteria (irRECIST). In the event of disease progression using the RECIST 1.1 criteria (unconfirmed PD (uPD)), investigators should continue treatment, if the patient is clinically stable (i.e. stable performance status and disease related symptoms) until disease progression is confirmed at a subsequent scan at least 4 weeks later (unequivocal confirmed disease progression (cPD)). This is particularly important for patients in whom pseudoprogression may have occurred. Follow up response assessments must be continued until unequivocal disease progression has occurred.

8.2.1 *Measurable Disease*.

Measurable *tumour lesions* are defined as those that can be accurately measured in at least one dimension (longest diameter to be recorded) as ≥ 20 mm with chest x-ray and as ≥ 10 mm with CT scan or clinical examination. Bone lesions are considered measurable only if assessed by CT scan and have an identifiable soft tissue component that meets these requirements (soft tissue component ≥ 10 mm by CT scan). *Malignant lymph nodes* must be ≥ 15 mm in the short axis to be considered measurable; only the short axis will be measured and followed. All tumour measurements must be recorded in millimetres (or decimal fractions of centimetres). Previously irradiated lesions are not considered measurable unless progression has been documented in the lesion.

8.2.2 *Non-measurable Disease*

All other lesions (or sites of disease), including small lesions are considered non-measurable disease. Bone lesions without a measurable soft tissue component, leptomeningeal disease, ascites, pleural/pericardial effusions, lymphangitis cutis/pulmonis, inflammatory breast disease, lymphangitic involvement of lung or skin and abdominal masses followed by clinical examination are all non-measurable. Lesions in previously irradiated areas are non-measurable, unless progression has been demonstrated.

8.2.3 *Target Lesions*

When more than one measurable tumour lesion is present at baseline all lesions up to *a maximum of 5 lesions total* (and a maximum of *2 lesions per organ*) representative of all involved organs should be identified as target lesions and will be recorded and measured at baseline. Target lesions should be selected on the basis of their size (lesions with the longest diameter), be representative of all involved organs, but in addition should be those that lend themselves to *reproducible repeated measurements*. Note that pathological nodes must meet the criterion of a short axis of ≥ 15 mm by CT scan and only the *short* axis of these nodes will contribute to the baseline sum. All other pathological nodes (those with short axis ≥ 10 mm but < 15 mm) should be considered non-target lesions. Nodes that have a short axis < 10 mm are considered non-pathological and should not be recorded or followed (see 8.2.4). At baseline, the sum of the target lesions (longest diameter of tumour lesions plus short axis of lymph nodes: overall maximum of 5) is to be recorded.

After baseline, a value should be provided on the CRF for all identified target lesions for each assessment, even if very small. If extremely small and faint lesions cannot be accurately measured but are deemed to be present, a default value of 5 mm may be used. If lesions are too small to measure and indeed are believed to be absent, a default value of 0 mm may be used.

8.2.4 *Non-target Lesions*

All non-measurable lesions (or sites of disease) plus any measurable lesions over and above those listed as target lesions are considered *non-target lesions*. Measurements are not required but these lesions should be noted at baseline and should be followed as “present” or “absent”.

8.2.5 *New Lesions*

The transitory appearance of new lesions has been well described for immunotherapies. All new lesions must be recorded on the CRF up to a maximum of 5 lesions total (and a maximum of 2 lesions per organ) representative of all involved organs, using standard RECIST 1.1 criteria for nodal and non-nodal disease. Each new lesion should be measured at all subsequent assessments but is NOT included in the sum of target lesions for evaluation of response or progression.

8.2.6 *RECIST 1.1 Response*

8.2.6.1 *RECIST 1.1 - Patients With Measurable Disease*

RECIST 1.1 response will be classified as outlined below:

Complete Response (CR): disappearance of *target* and *non-target* lesions and normalization of tumour markers. Pathological lymph nodes must have short axis measures < 10 mm (Note: continue to record the measurement even if < 10 mm and considered CR). Residual lesions (other than nodes < 10 mm) thought to be non-malignant should be further investigated (by cytology specialized imaging or other techniques as appropriate for individual cases *[Eisenhauer 2009]*) before CR can be accepted.

Partial Response (PR): at least a 30% decrease in the sum of measures (longest diameter for tumour lesions and short axis measure for nodes) of target lesions, taking as reference the baseline sum of diameters. Non target lesions must be non-PD.

Stable Disease (SD): Neither sufficient shrinkage to qualify for PR nor sufficient increase to qualify for PD taking as reference the smallest sum of diameters on study.

Progressive Disease (PD): at least a 20% increase in the sum of diameters of measured lesions taking as references the smallest sum of diameters recorded on study (including baseline) AND an absolute increase of ≥ 5 mm. Appearance of new lesions will also constitute progressive disease (including lesions in previously unassessed areas). In exceptional circumstances, unequivocal progression of non-target disease may be accepted as evidence of disease progression, where the overall tumour burden has increased sufficiently to merit discontinuation of treatment or where the tumour burden appears to have increased by at least 73% in volume. Modest increases in the size of one or more non-target lesions are NOT considered unequivocal progression. If the evidence of PD is equivocal (target or non-target), treatment may continue until the next assessment, but if confirmed, the earlier date must be used.

Table 1: Integration of Target, non-Target and New Lesions into Response Assessment *for patients with measurable disease*:

Target Lesions	Non-Target Lesions	New Lesions	Overall Response	Best Response for this Category also Requires
Target lesions ± non target lesions				
CR	CR	No	CR	Normalization of tumour markers, tumour nodes < 10 mm
CR	Non-CR/Non-PD	No	PR	
CR	Not all evaluated	No	PR	
PR	Non-PD/ not all evaluated	No	PR	
SD	Non-PD/ not all evaluated	No	SD	Documented at least once ≥ 4 weeks from baseline
Not all evaluated	Non-PD	No	NE	
PD	Any	Any	PD	
Any	PD	Any	PD	
Any	Any	Yes	PD	
Non target lesions ONLY				
No Target	CR	No	CR	Normalization of tumour markers, tumour nodes < 10 mm
No Target	Non-CR/non-PD	No	Non-CR/non-PD	
No Target	Not all evaluated	No	NE	
No Target	Unequivocal PD	Any	PD	
No Target	Any	Yes	PD	
Note: Patients with a global deterioration of health status requiring discontinuation of treatment without objective evidence of disease progression at that time should be reported as "symptomatic deterioration". This is a reason for stopping therapy, but is NOT objective PD. Every effort should be made to document the objective progression even after discontinuation of treatment.				

8.2.6.2 *RECIST 1.1 - Patients With Non-Measurable Disease Only*

Patients with only non-measurable (but evaluable) disease, may only have an overall RECIST 1.1 response or SD or PD as follows:

Complete Response (CR): disappearance of non-target lesions. Residual lesions thought to be non-malignant should be further investigated (by cytology specialized imaging or other techniques as appropriate for individual cases *[Eisenhauer 2009]* before CR can be accepted.

Stable Disease (SD): steady state of disease. No new lesions and not sufficient progression of non-target lesions to qualify for PD.

Progressive Disease (PD): the appearance of new lesions and/or unequivocal progression of non-target lesions.

Table 2: Integration of Target, non-Target and New Lesions into Response Assessment *for patients with only non-measurable, evaluable, lesions*:

<table>
<tr><th>Non-Measurable Lesions*</th><th>New Lesions</th><th>Overall Response</th><th>Best Overall Response for this category also requires</th></tr>
<tr><td>Complete disappearance</td><td>No</td><td>CR</td><td></td></tr>
<tr><td>Non-PD</td><td>No</td><td>SD</td><td>Documented at least once ≥ 4 weeks from baseline</td></tr>
<tr><td>PD**</td><td>No</td><td>PD</td><td rowspan="2">No prior SD</td></tr>
<tr><td>Any</td><td>Yes</td><td>PD</td></tr>
<tr><td colspan="4">* Note that these lesions should be recorded under the "Non-Target" lesions table on the CRFs.
** Unequivocal progression in non-measurable lesions will be accepted as disease progression.
Note: Patients with a global deterioration of health status requiring discontinuation of treatment without objective evidence of disease progression at that time should be reported as “symptomatic deterioration”. This is a reason for stopping therapy, but is NOT objective PD. Every effort should be made to document the objective progression even after discontinuation of treatment.</td></tr>
</table>

8.2.7 *irRECIST Response*

8.2.7.1 *irRECIST - Patients With Measurable Disease*

Table 3: Immune-Related RECIST Criteria (irRECIST) *for patients with measurable disease*:

Response	Description of Response	Confirmation by
Complete remission: irCR	Complete disappearance of all lesions (whether measurable or not, and no new lesions). Patient may have had prior uPD by RECIST 1.1 criteria.	Repeat, consecutive assessment no less than 4 weeks from the date first documented
Partial Remission: irPR	Decrease in tumour burden ≥ 30% relative to baseline. Patient may have had prior uPD by RECIST 1.1 criteria.	Consecutive assessment at least 4 weeks after first documentation
Stable Disease: irSD	Not meeting criteria for irCR or irPR, in absence of irPD. Patient may have had prior uPD by RECIST 1.1 criteria	
Progressive disease: irPD	Increase in tumour burden ≥ 20% relative to nadir (minimum recorded tumour burden)	Confirmation by a repeat, consecutive assessment no less than 4 weeks from the date first documented is required
New measurable lesions (i.e. ≥ 10 mm in long axis)	Measured but not incorporated into overall assessment of target lesions.	
New, non-measurable lesions	Recorded	
unidimensional measurements will be collected per RECIST 1.1		

8.2.7.2 *irRECIST - Patients With Non-Measurable Disease Only*

Table 4: Immune-Related RECIST Criteria (irRECIST) *for patients with only non-measurable, evaluable, lesions*:

Response	Description of Response	Confirmation by
Complete remission: irCR	Complete disappearance of all non-measurable lesions (and no new lesions). Patient may have had prior uPD by RECIST 1.1 criteria.	Repeat, consecutive assessment no less than 4 weeks from the date first documented
Stable Disease: irSD	Not meeting criteria for irCR in absence of irPD. Patient may have had prior uPD by RECIST 1.1 criteria.	
Progressive disease: irPD	Unequivocal increase in tumour burden	Confirmation by a repeat, consecutive assessment no less than 4 weeks from the date first documented is required
New measurable lesions (i.e. ≥ 10 mm in long axis)	Measured but not incorporated into of overall assessment of target lesions.	
New, non-measurable lesions	Recorded	

8.3 Response Duration

Response duration will be measured from the time measurement criteria for CR/PR (whichever is first recorded) are first met until the first date that recurrent or progressive disease is objectively documented, taking as reference the smallest measurements recorded on study (including baseline).

8.4 Stable Disease Duration

Stable disease duration will be measured from randomization until the criteria for progression are met, taking as reference the smallest sum on study (including baseline).

8.5 Methods of Measurement

The same method of assessment and the same technique should be used to characterize each identified and reported lesion at baseline and during follow-up. Assessments should be identified on a calendar schedule and should not be affected by delays in therapy. While on study, all lesions recorded at baseline should have their actual measurements recorded at each subsequent evaluation, even when very small (e.g. 2 mm). If it is the opinion of the radiologist that the lesion has likely disappeared, the measurement should be recorded as 0 mm. If the lesion is believed to be present and is faintly seen but too small to measure, a default value of 5 mm should be assigned. For lesions which fragment/split add together the longest diameters of the fragmented portions; for lesions which coalesce, measure the maximal longest diameter for the “merged lesion”.

8.5.1 *Clinical Lesions*

Clinical lesions will only be considered measurable when they are superficial and ≥ 10 mm as assessed using callipers (e.g. skin nodules). For the case of skin lesions, documentation by colour photography including a ruler to estimate the size of the lesion is recommended. If feasible, imaging is preferred.

8.5.2 *Chest X-ray*

Chest CT is preferred over chest X-ray, particularly when progression is an important endpoint, since CT is more sensitive than X-ray, particularly in identifying new lesions. However, lesions ≥ 20 mm on chest X-ray may be considered measurable if they are clearly defined and surrounded by aerated lung.

8.5.3 *CT, MRI*

CT is the best currently available and reproducible method to measure lesions selected for response assessment. This guideline has defined measurability of lesions on CT scan based on the assumption that CT slice thickness is 5 mm or less. When CT scans have slice thickness greater than 5 mm, the minimum size for a measurable lesion should be twice the slice thickness. MRI is also acceptable in certain situations (e.g. for body scans). Other specialized imaging or other techniques may also be appropriate for individual case *[Eisenhauer 2009]*. For example, while PET scans are not considered adequate to measure lesions, PET-CT scans may be used providing that the measures are obtained from the CT scan and the CT scan is of identical diagnostic quality to a diagnostic CT (with IV and oral contrast).

8.5.4 *Ultrasound*

Ultrasound is not useful in assessment of lesion size and should not be used as a method of measurement. If new lesions are identified by ultrasound in the course of the study, confirmation by CT is advised.

8.5.5 *Endoscopy, Laparoscopy*

The utilization of these techniques for objective tumour evaluation is not advised. However, they can be useful to confirm complete pathological response when biopsies are obtained or to determine relapse in trials where recurrence following complete response or surgical resection is an endpoint.

8.5.6 *Tumour Markers*

Tumour markers alone cannot be used to assess objective tumour response. If markers are initially above the upper normal limit, however, they must normalize for a patient to be considered in complete response.

8.5.7 *Cytology, Histology*

These techniques can be used to differentiate between PR and CR in rare cases if required by protocol (for example, residual lesions in tumour types such as germ cell tumours, where known residual benign tumours can remain). When effusions are known to be a potential adverse effect of treatment (e.g. with certain taxane compounds or angiogenesis inhibitors), the cytological confirmation of the neoplastic origin of any effusion that appears or worsens during treatment when the measurable tumour has met criteria for response or stable disease is advised to differentiate between response or stable disease and progressive disease.

9.0 SERIOUS ADVERSE EVENT REPORTING

The descriptions and grading scales found in the NCI Common Terminology Criteria for Adverse Events (CTCAE) will be utilized for Adverse Event (AE) reporting (version can be found in Appendix V). All appropriate treatment areas should have access to a copy of the CTCAE. A copy of the CTCAE can be downloaded from the CTEP web site: (http://ctep.cancer.gov/protocolDevelopment/electronic_applications/ctc.htm).

All serious adverse events (SAE) defined as per ICH guidelines (see below) and other adverse events must be recorded on case report forms. In addition, all "reportable" serious adverse events are subject to expedited reporting using the CCTG SAE form. The term 'reportable SAE' is used in the definitions which follow to describe those SAEs which are subject to expedited reporting to CCTG.

9.1 Definition of a Reportable Serious Adverse Event *(Durvalumab +/- Tremelimumab Arm)*

- For patients randomized to receive best supportive care (BSC), SAE reporting is NOT required.
- All serious adverse events, regardless of whether they are unexpected or related to protocol treatment, occurring during the treatment period and within 30 days after the last protocol treatment administration, must be reported in an expedited manner. Any late serious adverse event occurring after this 30-day period which is related to protocol treatment must also be reported in an expedited manner (see Section 9.2 for reporting instructions).
- A serious adverse event (SAE) is any adverse event that at any dose:
  - results in death
  - is life-threatening
  - requires inpatient hospitalization or prolongation of existing hospitalization (excluding hospital admissions for study drug administration, transfusional support, scheduled elective surgery and admissions for palliative or terminal care)
  - results in persistent or significant disability or incapacity
  - is a congenital anomaly/birth defect

Medical and scientific judgement should be exercised in deciding whether expedited reporting is appropriate in other situations such as important medical events that may not be immediately life-threatening or result in death or hospitalization but may jeopardize the patient or may require intervention to prevent one of the events listed above.

Important Notes:

1. Any immune-related adverse event (irAE) requiring high dose steroids is by definition medically significant and must be reported as such (i.e., as expedited events using the SAE reporting system).
2. If a patient shows an AST or ALT $\geq$ 3 x ULN together with total bilirubin $\geq$ 2 x ULN, refer to Appendix III for further instructions on cases of increases in liver biochemistry and evaluation of Hy's Law. These cases should be reported as SAEs if, after evaluation, they meet the criteria for a Hy's law case or if any of the individual liver test parameters fulfill any of the SAE criteria.

9.1.1 *Durvalumab +/- Tremelimumab Adverse Events of Special Interest*

An adverse event of special interest (AESI) is one of scientific and medical interest specific to understanding of the Investigational Product and may require close monitoring. An AESI may be serious or non-serious.

AESIs for durvalumab ± tremelimumab include, but are not limited to events with a potential inflammatory or immune-mediated mechanism and which may require more frequent monitoring and/or interventions such as steroids, immunosuppressants and/or hormone replacement therapy. **These AESIs may require close monitoring in the treatment arms with durvalumab ± tremelimumab.**

An immune-mediated adverse event (imAE) is defined as an AESI that is associated with drug exposure and is consistent with an immune-mediated mechanism of action and where there is no clear alternate etiology. Serologic, immunologic, and histologic (biopsy) data, as appropriate, should be used to support an imAE diagnosis. Appropriate efforts should be made to rule out neoplastic, infectious, metabolic, toxin, or other etiologic causes of the imAE.

If the Investigator has any questions in regards to an event being an imAE, the Investigator should promptly contact the CCTG.

AESIs observed with durvalumab ± tremelimumab include:

- Diarrhea / Colitis, and intestinal perforation;
- Pneumonitis / Interstitial Lung Disease (ILD);
- ALT/AST increases / hepatitis / hepatotoxicity;
- Neuropathy / neuromuscular toxicities (e.g. Guillain-Barré syndrome, and myasthenia gravis);
- Endocrinopathies (i.e. events of hypophysitis, hypopituitarism, adrenal insufficiency, diabetes insipidus, hyper- and hypothyroidism and type I diabetes mellitus);
- Rash / Dermatitis;
- Nephritis / Blood creatinine increases;
- Pancreatitis (or labs suggestive of pancreatitis - increased serum lipase , increased serum amylase);
- Myocarditis;
- Myositis/polymyositis
- Other inflammatory responses that are rare with a potential immune-mediated aetiology include, but are not limited to, pericarditis, sarcoidosis, uveitis and other events involving the eye, skin, hematological and rheumatological events.

In addition, infusion-related reactions and hypersensitivity/anaphylactic reactions with a different underlying pharmacological etiology are also considered AESIs.

Further information on these risks (e.g. presenting symptoms) can be found in the current version of the durvalumab and tremelimumab Investigator’s Brochures. More specific guidelines for their evaluation and treatment are described in detail in Appendix III.

9.1.2 *Events **Not** to be Treated as SAEs (Durvalumab +/- Tremelimumab Arm)*

- Serious adverse events which are unequivocally related to the underlying malignancy or disease progression do **NOT** require expedited reporting. These include such adverse events as admission for pain control, palliative care or paracentesis of malignant effusions.
- In addition, the following events will **NOT** be recorded as AEs (or SAEs):
  - lack of efficacy /disease progression (will be recorded separately on CRF);
  - laboratory abnormalities for protocol specified tests (these are derived electronically from actual values supplied and need not be reported separately in adverse event tables on CRFs);
  - elective hospitalization for medical, radiological or surgical procedures for treatment of disease or to simplify treatment for study procedures (will be recorded separately on CRF);
  - hospitalization for palliative care or pain control.

9.2 Serious Adverse Event Reporting Instructions

All reportable serious adverse events must be reported using a web-based Electronic Data Capture (EDC) system being used for this trial. For details about accessing the EDC system and completing the on-line SAE report form, please refer to the CCTG Generic Data Management Guidebook for EDC Studies posted on the CO.26 section of the CCTG website (www.ctg.queensu.ca).

Within 24 hours:	Complete preliminary Serious Adverse Event Report and submit to CCTG via EDC system.
Within 10 days:	Update Serious Adverse Event Report as much as possible and submit report to CCTG via EDC system.

EDC SAE web application interruption:
In the rare event that internet connectivity to the EDC SAE system is disrupted, please print and complete a paper copy of the SAE Report, available from the trial specific website.

FAX paper SAE Report to:

CO.26 Study Coordinator
Canadian Cancer Trials Group
Fax No.: 613-533-2941

Please use the same timelines for submission as for direct EDC reporting.

Once internet connectivity is restored, the information that was FAXED to CCTG on the paper SAE Report must also be entered by the site into the EDC SAE web application.

Local internet interruption:
If you are unable to access the EDC SAE system, and cannot access a paper copy of the SAE Report from the trial website, please phone the CO.26 trial team (613-533-6430) to obtain a copy of the SAE Report by FAX. Once completed, the report must be FAXED back to CCTG as indicated above. Once internet connectivity is restored, the information that was FAXED to CCTG on the paper SAE Report must also be entered by the site into the EDC SAE web application.

In cases of prolonged internet interruptions, please contact the CCTG Safety Desk for further instructions (613-533-6430).

9.3 Other Protocol Reportable Events – Pregnancy Reporting

9.3.1 *Pregnancy Prevention*

Women of Childbearing Potential (WOCBP) and males who are enrolled in the trial must have agreed to use contraceptive method(s) as described in Eligibility Criterion 4.1.15. Investigators may wish to additionally advise the female partners of male participants about pregnancy prevention guidelines when appropriate and compliant with local policy.

A highly effective method of contraception is defined as one that results in a low failure rate (i.e. less than 1% per year) when used consistently and correctly. Highly effective methods of contraception are described in the table below. Note that some contraception methods are not considered highly effective (e.g. male or female condom with or without spermicide; female cap, diaphragm, or sponge with or without spermicide; non-copper containing intrauterine device; progestogen-only oral hormonal contraceptive pills where inhibition of ovulation is not the primary mode of action [excluding Cerazette/desogestrel which is considered highly effective]; and triphasic combined oral contraceptive pills).

Highly Effective* Methods of Contraception	
Barrier/Intrauterine Methods	Hormonal Methods
• Copper T intrauterine device<br>• Levonorgesterel-releasing intrauterine system (e.g. Mirena®)**	• Etonogestrel implants: e.g. Implanon or Norplan<br>• Intravaginal device: e.g. ethinylestradiol and etonogestrel<br>• Medroxyprogesterone injection: e.g. Depo-Provera<br>• Normal and low dose combined oral contraceptive pill<br>• Norelgestromin/ethinylestradiol transdermal system<br>• Cerazette (desogestrel)
* Highly effective (i.e. failure rate of <1% per year).<br>** This is also considered a hormonal method.	

9.3.2 *Pregnancy Reporting*

If a patient becomes pregnant during the course of the study, the investigational agent should be discontinued immediately.

Pregnancy itself - occurring in female participants, and female partners of male participants - is not regarded as an AE unless there is a suspicion that the IP under study may have interfered with the effectiveness of a contraceptive medication. The outcome of all pregnancies (spontaneous miscarriage, elective termination, ectopic pregnancy, normal birth, or congenital abnormality) should be followed up and documented even if the patient was discontinued from the study.

The investigator is required to report to CCTG any pregnancy occurring in female participants, and female partners of male participants. Pregnancies occurring up to 6 months after the last dose of study treatment must also be reported.

The investigator should report the pregnancy in a timely manner, within 24 hours of learning of the pregnancy using the CCTG Pregnancy Reporting Form available from the trial webpage.

Once informed consent has been obtained, the form should be updated to provide further pregnancy information and to reflect the outcome of the pregnancy. All follow-up reports must be submitted to CCTG in a timely manner. For pregnant partner of trial participant (and pregnant participants, if required by local policy), a copy of the signed signature page of the pregnancy follow-up consent must be submitted to CCTG.

Documents outlined above (including updates) must be sent to the CCTG safety desk (613-533-2812/ safety-desk@ctg.queensu.ca).

If the pregnancy results in death (e.g. spontaneous abortion, stillbirth); is life-threatening; requires inpatient hospitalization or prolongation of existing hospitalization; results in persistent or significant disability/incapacity; is a congenital anomaly/birth defect, then an SAE report must be additionally submitted as described above. Please note, hospitalization for labour/delivery alone does not constitute an 'inpatient hospitalization' for the purposes of pregnancy reporting.

9.4 CCTG Responsibility for Reporting Serious Adverse Events to Health Canada

The CCTG will provide expedited reports of SAEs to Health Canada (Office of Clinical Trials) for those events which meet regulatory requirements for expedited reporting, i.e. events which are BOTH serious AND unexpected, AND which are thought to be related to protocol treatment (or for which a causal relationship with protocol treatment cannot be ruled out).

9.5 CCTG Reporting Responsibility to AstraZeneca

AstraZeneca will be notified of all protocol reportable serious adverse events (as defined in Section 9.1) within one working day of receipt of report at CCTG. CCTG, as sponsor, will determine regulatory reportability in Canada.

AstraZeneca will be notified of all pregnancies and outcomes of pregnancies within 30 days of receipt of the report at CCTG.

9.6 AstraZeneca Reporting Responsibilities

AstraZeneca will report all regulatory reportable serious adverse events from non-CCTG trials (Safety Updates) with durvalumab and tremelimumab to CCTG within the timelines outlined in the contract. CCTG will review these events to determine which meet the criteria (serious, unexpected, drug related) for reporting to CO.26 investigators. AstraZeneca will report these events to Health Canada.

9.7 Reporting Safety Reports to Investigators

CCTG will notify Investigators of all Safety Reports (Serious Adverse Events (SAEs) from this trial and Safety Updates (SUs) from other clinical trials) that are reportable to regulatory authorities in Canada as reported to the CCTG. This includes all serious events that are unexpected and related (i.e. possibly, probably, or definitely) to protocol treatment. The reports will be posted to the CCTG trial CO.26 web-based safety monitoring utility.

Investigators must notify their Research Ethics Boards (REBs) of events which involve corrective action(s) to be taken as a result of the event(s) such as protocol and/or informed consent changes. The date of REB Submission for these SAEs and SUs will need to be entered into the CCTG trial CO.26 web-based safety monitoring utility and documentation of REB submission must be retained in the study binder on site. The REB submission template provided by CCTG can be used to assist with tracking, submission, filing and monitoring.

The submission of events to your ethics board should be done as soon as possible (we suggest within 30 days). REB submissions greater than 90 days from the date of notification will be regarded as delinquent and a major deficiency will be assigned. These safety reports are to be filed in the trial files on site.

10.0 PROTOCOL TREATMENT DISCONTINUATION AND THERAPY AFTER STOPPING

10.1 Criteria for Discontinuing Protocol Treatment

Patients may stop protocol treatment in the following instances:

- Intercurrent illness which would, in the judgement of the investigator, affect assessments of clinical status to a significant degree, and require discontinuation of protocol therapy.
- Unacceptable toxicity as defined in Section 7.0 and Appendix III.
- Tumour progression or disease recurrence as defined in Section 8.0.
- Request by the patient.

Efforts should be made to maintain the investigations schedule and continue follow-up, even if patients discontinue protocol treatment prematurely and/or no longer attend the participating institution.

10.2 Duration of Protocol Treatment

Investigators are encouraged to continue therapy, as appropriate in the absence of unacceptable toxicity, until disease progression has been unequivocally documented. This is particularly important for patients in whom pseudoprogression may have occurred. In patients for whom study therapy is continued beyond the first documentation of objective disease progression (i.e. per RECIST 1.1 criteria) on the basis of clinical benefit, continued follow-up assessments per protocol must also be continued until disease progression has been unequivocally confirmed, either through RECIST 1.1 or Immune-Related Response Criteria (irRC) *[Wolchok 2009]* (i.e. treatment failure), at which time study therapy should cease.

10.3 Therapy After Protocol Treatment is Stopped

At the discretion of the investigator.

11.0 CENTRAL REVIEW PROCEDURES

11.1 Central Radiology Review

There will be no central radiology review for this study.

11.2 Central Pathology Review

There will be no central pathology review for this study.

12.0 CORRELATIVE STUDIES

Tissue samples collected on this trial may be used by researchers now or in the future to better understand the nature of colorectal cancer and how patients respond to treatment. Samples will be used for research purposes only and will not be sold. A scientific review process of any proposals to use the tissue will take place and any proposals approved will have undergone ethics approval. Patients will not be identified by name. The only identification of tissue will be by a tumour banking code assigned at the time of sample receipt. Material issued to researchers will be anonymized and only identified by tumour banking coded number.

Specific samples to be collected and priority assays are as follows:

12.1 Protocol-Mandated Correlative Studies

Archival FFPE Tumour Tissue Submission (Mandatory)

Archival Tumour Block/Slides:

The submission of a representative block of the diagnostic tumour tissue (from primary or metastatic tumour) and the adjacent normal tissue (part of the standard resection) is mandatory for participation on this trial. Blocks will be carefully stored as part of the CCTG Tumour Tissue Data Repository at Queen’s University in Kingston, Ontario, but the only assays done will be as part of this study. All patients on whom a diagnostic tumour block is collected will be aware of this retrieval and will have given their consent.

Blocks are the preferred material to collect, as it is well known that tissue materials (including protein and nucleic acid integrity) on unstained sections deteriorate rapidly within 3-6 months after preparation. One of the objectives will be to create tissue micro arrays. These will optimize the amount of tissue available to investigators and permit the preservation of the tumour block submitted.

If, at any time, the submitting hospital requires the block to be returned for medical or legal concerns, it will be returned by courier on request.

Samples will be used for research purposes only and will not be sold. Patients will not be identified by name. The only identification of tissue will be by a patient study number assigned at the time of registration to the trial, the surgical/ histology number and/or patient initials. Material issued to researchers will be anonymized and only identified by a coded number.

Testing specifically for hereditary genetic defects predisposing to malignant disease will not be carried out without the expressed consent of the patient.

Where no previously resected or biopsied tumour tissue exists, additional biopsy of the primary or metastatic tumour will be required.

Directions:

At the same time that the baseline form is submitted, original tumour block should be sent to the CCTG Pathology Coordinator. Centres should contact CCTG if they are unable to submit a tumour block, as sufficient tissue is required for the assays described above.

Within 4 weeks of randomization (note: a request letter will not be sent), complete the EDC Archival Tumour Tissue Submission Form. Print a copy of the completed form and ship tumour blocks/slides along with a Request for Payment form to:

Shakeel Virk
Pathology Coordinator, Canadian Cancer Trials Group
Richardson Labs Bldg, 4th Floor
88 Stuart St.
Queen’s University
Kingston, ON K7L 3N6
Tel: 613-533-2906
Fax: 613-548-2486
Email: virks@queensu.ca

Detailed instructions for FFPE sample acquisition, preparation, and shipping are found in the CO.26 Lab Manual.

Planned priority assays on archival tumour tissue include:

- Immunohistochemistry for PD-L1 and CTLA-4 expression;
- Immunohistochemistry for tumour infiltrating lymphocytes (CD8+ T cells);
- Other exploratory analysis by IHC and/or flow cytometry for more detailed characterization of immune cell population / additional immune markers (IDO, CD4, CD3, FOXP3);
- Immunohistochemistry for mismatch repair (MMR) proteins;
- Genomic instability measured by MSI assays or using SNP arrays;
- Mutational load measured using targeted next generation sequencing;
- Expression profiling to identify microsatellite instability immune, canonical, metabolic and mesenchymal subtypes of tumour.

Blood, Serum and Plasma Collection:

The CCTG is interested in exploring the use of surrogate tissues such as serum and plasma in evaluating potential prognostic or predictive biomarkers, or as evidence of pharmacodymanic effects. Blood, serum and plasma samples will be collected and banked for planned studies from all patients.

Planned priority assays on blood, serum and plasma include:

- Circulating tumour DNA

Detailed instructions for blood, serum and plasma sample acquisition, preparation, and shipping are found in the CO.26 Lab Manual.

12.2 Optional Banking of Samples

Banking of Tumour Tissue:

Mandatory submission of tumour tissue has been described above. The subsequent banking of collected diagnostic tissue is not mandatory for participation in the study, but the participation of all centres is strongly encouraged. Blocks and blood will be carefully banked as part of the CCTG tissue/tumour bank at Queen's University in Kingston, Ontario.

After patient consent, collection of paraffin tumour blocks will preferred, as one of the objectives will be to create tissue micro arrays. These will optimize the amount of tissue available to investigators and permit the preservation of the tumour block submitted. If tumour blocks are unavailable, then two x 2 mm cores of tumour from the block and 30 specimen slides are preferred. If, at any time, the submitting hospital requires the block to be returned for medical or legal concerns, it will be returned by courier on request.

Proposals to use the banked specimens for the purposes of assessing markers involved in predicting treatment response and outcomes may be submitted to the bank. A scientific review process of any proposals to use the tissue will take place and any proposals approved will have undergone ethics approval.

Banking of Blood, Serum, and Plasma:

Mandatory submission of whole blood, serum, and plasma has been described above. The subsequent banking of collected samples is not mandatory for participation in the study, but the participation of all centres is strongly encouraged. Samples will be carefully banked as part of the CCTG tissue/tumour bank at Queen's University in Kingston, Ontario.

Proposals to use the banked specimens for the purposes of assessing markers involved in predicting treatment response and outcomes may be submitted to the bank. A scientific review process of any proposals to use the tissue will take place and any proposals approved will have undergone ethics approval.

13.0 STATISTICAL CONSIDERATIONS

13.1 Objectives and Design

The primary objective of this phase II randomized study is to assess the effect of the combination of durvalumab and tremelimumab and best supportive care (BSC) on the Overall Survival of patients with refractory, advanced colorectal cancer. Patients will be randomized to receive either combination of durvalumab and tremelimumab plus best supportive care, or best supportive care only in a 2:1 ratio after stratification by ECOG performance status (0 versus 1). Secondary objectives include comparisons of Progression Free Survival, Objective Response Rate, and Adverse Events between the two treatment arms.

13.2 Study Endpoints and Analysis

Overall Survival:
Overall Survival, the primary endpoint of this study, is defined as the time from randomization to death from any cause. Patients who are alive at the time of the final analysis or who have become lost to follow-up will be censored at their last date known to be alive. Patients will be analyzed in the arm to which they are randomized regardless of the treatment they received (intent-to-treat analysis). The survival experience of patients in both treatment groups will be summarized by the Kaplan-Meier method and compared primarily by a stratified log-rank test adjusting for the stratification variable (ECOG performance status and site of tumour) at randomization. Secondary analyses based on stratified Cox proportional hazards model will also be performed. ECOG performance status (0 versus 1) and site of tumour (right colon, transverse colon, left colon, rectum, unknown) will be the stratification factors to define the stratified Cox proportional hazards model. Besides the treatment factor (combination of durvalumab and tremelimumab + BSC versus BSC alone), the following factors at patient entry will be included in the stratified Cox proportional hazards model:

- Age (< 65 versus ≥ 65)
- Sex (male versus female)
- Number of organ sites involved at baseline (≤ 2 versus > 2)
- Presence of liver metastases (yes versus no)

A formal pre-planned subset analysis for the primary endpoint (OS) will be conducted to address the benefit of combination of durvalumab and tremelimumab between the groups defined by the above factors and the following:

- ECOG Performance Status (0 versus 1)
- Race (white, black, Asian, other)
- Site of tumour: (right colon, transverse colon, left colon, rectum)

Progression-Free Survival:
Progression-Free Survival (PFS) is defined as the time from randomization to the first objective documentation of disease progression or death due to any cause. If a patient has not progressed or died at the time of final analysis, PFS will be censored on the date of the last tumour assessment. This includes patients who are lost to follow-up or have withdrawn consent. All analyses for OS will also be performed for PFS, using similar methodology. A sensitivity analysis of PFS will also be performed where PFS is defined as the time from randomization to the second documentation of unequivocal disease progression in those patients who continued study therapy beyond the first objective documentation of disease progression and with interim CR, PR or SD documented.

Objective Response Rate:
Objective Response Rate (ORR) is defined as the proportion of patients with a documented complete response and partial response based on RECIST 1.1. The primary estimate of ORR will be based on all patients randomized. A Cochran-Mantel-Haenszel test adjusting for the stratification factors (ECOG performance status, site of tumour) at the time of randomization will be used to compare the objective response rates between two arms. A sensitivity analysis of ORR will also be performed where response is defined as the proportion of patients with a documented complete response and partial response based on RECIST 1.1 and/or Immune-Related Response Criteria (irRC) *[Wolchok 2009]*.

Safety Analysis:
All patients who have received at least one dose of protocol treatment will be included in the safety analysis. The incidence of adverse events will be summarized by type of adverse event and severity using the NCI Common Terminology Criteria for Adverse Events. A Fisher's exact test will be used to compare adverse events between the two arms if required.

13.3 Sample Size and Duration of Study

The study is designed to have a power of 80% and a two-sided alpha of 10% to detect a 35% reduction in the continuous risk of death (HR 0.65, which corresponds to an increase of median survival from 4.5 to 6.9 months). It is estimated that 150 events will be required to detect this reduction. The final analysis will be performed when 150 events are observed. Assuming an accrual rate of around 10 patients per month, the required number of events would be observed by accruing a total of 180 patients and following them for a minimum of 6 months. The total duration of the trial would be around 24 months.

13.4 Safety Monitoring

Adverse events will be monitored on an ongoing basis by the central office and their frequencies reported annually at investigators' meetings.

13.5 Interim Analysis

No interim analysis will be performed.

14.0 PUBLICATION POLICY

14.1 Authorship of Papers, Meeting Abstracts, Etc.

14.1.1 The results of this study will be published. Prior to trial activation, the chair will decide whether to publish the trial under a group title, or with naming of individual authors. If the latter approach is taken, the following rules will apply:

- The first author will generally be the chair of the study.
- A limited number of the members of the Canadian Cancer Trials Group may be credited as authors depending upon their level of involvement in the study.
- Additional authors, up to a maximum of 15, will be those who have made the most significant contribution to the overall success of the study. This contribution will be assessed, in part but not entirely, in terms of patients enrolled and will be reviewed at the end of the trial by the study chair.
- In the event of a separate paper dealing with the quality of life outcomes, the first author will generally be the Quality of Life Coordinator on the trial committee.

14.1.2 In an appropriate footnote, or at the end of the article, the following statement will be made:

> "A study coordinated by the Canadian Cancer Trials Group. Participating investigators included: (a list of the individuals who have contributed patients and their institutions)."

14.2 Responsibility for Publication

It will be the responsibility of the Study Chair to write up the results of the study within a reasonable time of its completion. If after a period of six months following the analysis of study results the draft is not substantially complete, the central office reserves the right to make other arrangements to ensure timely publication.

14.3 Submission of Material for Presentation or Publication

Material may not be submitted for presentation or publication without prior review by the CCTG Senior Investigator, Senior Biostatistician, Study Coordinator, and approval of the Study Chair. Individual participating centres may not present outcome results from their own centres separately. Supporting groups and agencies will be acknowledged.

15.0 ETHICAL, REGULATORY AND ADMINISTRATIVE ISSUES

15.1 Regulatory Considerations

All institutions in Canada must conduct this trial in accordance with International Conference on Harmonization-Good Clinical Practice (ICH-GCP) Guidelines.

This trial is being conducted under a Clinical Trial Application (CTA) with Health Canada. As a result, the conduct of this trial must comply with Division 5 of the Canadian Regulations Respecting Food and Drugs (Food and Drugs Act).

15.2 Inclusivity in Research

CCTG does not exclude individuals from participation in clinical trials on the basis of attributes such as culture, religion, race, national or ethnic origin, colour, mental or physical disability (except incapacity), sexual orientation, sex/gender, occupation, ethnicity, income, or criminal record, unless there is a valid reason (i.e. safety) for the exclusion.

In accordance with the Declaration of Helsinki and the Tri-Council Policy Statement (TCPS), it is the policy of CCTG that vulnerable persons or groups will not be automatically excluded from a clinical trial (except for incompetent persons) if participation in the trial may benefit the patient or a group to which the person belongs.

However, extra protections may be necessary for vulnerable persons or groups. It is the responsibility of the local investigator and research ethics board (REB) to ensure that appropriate mechanisms are in place to protect vulnerable persons/groups. In accordance with TCPS, researchers and REBs should provide special protections for those who are vulnerable to abuse, exploitation or discrimination. As vulnerable populations may be susceptible to coercion or undue influence, it is especially important that informed consent be obtained appropriately.

Centres are expected to ensure compliance with local REB or institutional policy regarding participation of vulnerable persons/groups. For example, if a vulnerable person/group would be eligible for participation in a CCTG clinical trial under this policy but excluded by local policy, it is expected that they would not be enrolled in the trial. It is the centre's responsibility to ensure compliance with all local SOPs.

It is CCTG's policy that persons who cannot give informed consent (i.e. mentally incompetent persons, or those physically incapacitated such as comatose persons) are not to be recruited into CCTG studies. It is the responsibility of the local investigator to determine the subject's competency, in accordance with applicable local policies and in conjunction with the local REB (if applicable).

Subjects who were competent at the time of enrolment in the clinical trial but become incompetent during their participation do not automatically have to be removed from the study. When re-consent of the patient is required, investigators must follow applicable local policies when determining if it is acceptable for a substitute decision maker to be used. CCTG will accept re-consent from a substitute decision maker. If this patient subsequently regains capacity, the patient should be re-consented as a condition of continuing participation.

15.3 Obtaining Informed Consent

It is expected that consent will be appropriately obtained for each participant/potential participant in a CCTG trial, in accordance with ICH-GCP section 4.8. The centre is responsible for ensuring that all local policies are followed.

Additionally, in accordance with GCP 4.8.2, CCTG may require that participants/potential participants be informed of any new information may impact a participant's/potential participant's willingness to participate in the study.

Based upon applicable guidelines and regulations (Declaration of Helsinki, ICH-GCP), a participating investigator (as defined on the participants list) is ultimately responsible, in terms of liability and compliance, for ensuring informed consent has been appropriately obtained. CCTG recognizes that in many centres other personnel (as designated on the participants list) also play an important role in this process. In accordance with GCP 4.8.5, it is acceptable for the Qualified Investigator to delegate the responsibility for conducting the consent discussion.

CCTG requires that each participant sign a consent form prior to their enrolment in the study to document his/her willingness to take part. CCTG may also require, as indicated above, that participants/potential participants be informed of new information if it becomes available during the course of the study. In conjunction with GCP 4.8.2, the communication of this information should be documented.

CCTG allows the use of translators in obtaining informed consent. Provision of translators is the responsibility of the local centre. Centres should follow applicable local policies when procuring or using a translator for the purpose of obtaining informed consent to participate in a clinical trial.

In accordance with ICH-GCP 4.8.9, if a subject is unable to read then informed consent may be obtained by having the consent form read and explained to the subject.

15.3.1 *Obtaining Consent for Pregnancy*

Information from and/or about the subject (i.e. the pregnant female, the newborn infant, male partner, exposed individual) should not be collected about or from them unless or until they are a willing participant in the research. The rights and protections offered to participants in research apply and consent must be obtained prior to collecting any information about or from them. If the main consent form adequately addresses the collection of information regarding the outcome of a pregnancy of a trial participant, a "Pregnancy Follow-up consent form will not be required by CCTG.

Trial-specific consent forms for "Pregnancy Follow-up" can be found on the trial webpage. The appropriate consent form must be used to obtain consent from any non-trial participant (such as the pregnant partner or exposed individual).

Participants will not be withdrawn from the main trial as a result of refusing or withdrawing permission to provide information related to the pregnancy. Similarly, male participants will not be withdrawn from the main study should their partner refuse/withdraw permission.

Obtaining Consent for Research on Children

In the case of collecting information about a child (i.e. the child resulting from a pregnant participant/partner or an exposed child), consent must be obtained from the parent/guardian.

15.4 Discontinuation of the Trial

If this trial is discontinued for any reason by the CCTG all centres will be notified in writing of the discontinuance and the reason(s) why. If the reason(s) for discontinuance involve any potential risks to the health of patients participating on the trial or other persons, the CCTG will provide this information to centres as well.

If this trial is discontinued at any time by the centre (prior to closure of the trial by the CCTG), it is the responsibility of the qualified investigator to notify the CCTG of the discontinuation and the reason(s) why.

Whether the trial is discontinued by the CCTG or locally by the centre, it is the responsibility of the qualified investigator to notify the local Research Ethics Board and all clinical trials subjects of the discontinuance and any potential risks to the subjects or other persons.

15.5 Retention of Patient Records and Study Files

All essential documents must be maintained as per C.05.012 and in accordance with ICH-GCP.

The Qualified Investigator must ensure compliance with the Regulations and the GCP Guideline from every person involved in the conduct of the clinical trial at the site.

Essential documents must be retained for 25 years following the completion of the trial at the centre (25 years post final analysis, last data collected, or closure notification to REB, whichever is later), or until notified by CCTG that documents no longer need to be retained.

In accordance with GCP 4.9.7, upon request by the monitor, auditor, REB or regulatory authority, the investigator/institution must make all required trial-related records available for direct access.

CCTG will inform the investigator/institution as to when the essential documents no longer need to be retained.

15.6 Centre Performance Monitoring

This study is eligible for inclusion in the Centre Performance Index (CPI).

Forms are to be submitted according to the schedule in the protocol. There are minimum standards for performance.

15.7 On-Site Monitoring/Auditing

CCTG site monitoring/auditing will be conducted at participating centres in the course of the study as part of the overall quality assurance program. The monitors/auditors will require access to patient medical records to verify the data, as well as essential documents, standard operating procedures (including electronic information), ethics and pharmacy documentation (if applicable).

As this trial is conducted under a CTA with Health Canada, your site may be subject to an inspection by the Health Canada Inspectorate.

AstraZeneca has reserved the right to audit participating centres. Audits may only be conducted after consultation with CCTG.

15.8 Case Report Forms

A list of forms to be submitted, as well as expectation dates, are to be found in Appendix IV.

This trial will use a web-based Electronic Data Capture (EDC) system for all data collection. For details of accessing the EDC system and completing the on-line Case Report Forms please refer to the “Registration/Randomization and Data Management Guidebook” posted on the CO.26 area of the CCTG web-site (www.ctg.queensu.ca).

16.0 REFERENCES

Andorsky DJ, Yamada RE, Said J, Pinkus GS, Betting DJ, and Timmerman JM. Programmed death ligand 1 is expressed by non-hodgkin lymphomas and inhibits the activity of tumour-associated T cells. Clin Cancer Res. 2011. 17(13):4232-4244

Antonia[a] SJ, Brahmer JR, Gettinger SN, Chow LQM, Juergens RA, Shepherd FA, Laurie SA, Gerber DE, Goldman JW, Shen Y, Harbison C, Alaparthy S, Chen AC, Borghaei H, Rizvi NA. Nivolumab (anti-PD-1; BMS-936558, ONO-4538) in combination with platinum-based doublet chemotherapy (PT-DC) or erlotinib (ERL) in advanced non-small cell lung cancer (NSCLC) Abstract presented at the European Society for Medical Oncology (ESMO) Annual Meeting, Madrid, Spain, September 26–30, 2014a.

Antonia[b] S., Ou S.-H., Khleif S.N., Brahmer J., Blake-Haskins A., Robbins P.B., et al. Clinical activity and safety of MEDI4736, an anti-programmed cell death ligand-1 (PD-L1) antibody, in patients with NSCLC. Poster presented at the European Society for Medical Oncology (ESMO) Annual Meeting, Madrid, Spain, September 26–30, 2014. Ann Oncol. 2014;25(suppl 4):iv466 (1325P), 2014b.

Antonia[a] SJ, Goldberg SB, Balmanoukian AS et al. Phase Ib study of MEDI4736, a programmed cell death ligand-1 (PD-L1) antibody, in combination with tremelimumab, a cytotoxic T-lymphocyte-associated protein-4 (CTLA-4) antibody, in patients (pts) with advanced NSCLC. J Clin Oncol 33, 2015a (suppl; abstr 3014).

Antonia[b] SJ, Bendell J, Taylor M, et al. Phase I/II study (CheckMate 032) of nivolumab with or without ipilimumab for treatment of recurrent small cell lung cancer (SCLC). J Clin Oncol 33, 2015b (suppl; abstr 7503).

Brahmer JR, Drake CG, Wollner I, Powderly JD, Picus J, et al. Phase I study of single agent anti-programmed death-1 (MDX-1106) in refractory solid tumours: safety, clinical activity, pharmacodynamics, and immunologic correlates. J Clin Oncol. 2010 Jul 1;28(19):3167-75.

Brahmer JR, Tykodi SS, Chow LQ et al. Safety and activity of anti-PD-L1 antibody in patients with advanced cancer. N Engl J Med 2012;366: 2455-65.

Brusa D, Serra S, Coscia M, Rossi D, D'Arena G, Laurenti L, et al. The PD-1/PD-L1 axis contributes to T-cell dysfunction in chronic lymphocytic leukemia. Haematologica. 2013 Jun; 98(6):953-963.

Canadian Cancer Statistics 2015, Canadian Cancer Society, 2015.

Eisenhauer E, Therasse P, Bogaerts J, Schwartz LH, Sargent D, Ford R, Dancey J, Arbuck S, Gwyther S, Mooney M, Rubinstein L, Shankar L, Dodd L, Kaplan R, Lacombe D, Verweij J. New response evaluation criteria in solid tumours: Revised RECIST guideline version 1.1. Eur J Can 45: 228-47, 2009.

Fife BT, Pauken KE, Eagar TN et al. Interactions between PD-1 and PD-L1 promote tolerance by blocking the TCR-induced stop signal. Nat Immunol 2009; 10: 1185-1192.

Gatalica Z, Snyder C, Maney T, Ghazalpour A, Holterman DA, Xiao N, et al. Programmed cell death 1 (PD-1) and its ligand (PD-L1) in common cancers and their correlation with molecular cancer type. Cancer Epidemiol Biomarkers Prev. 2014 Dec;23(12):2965-70.

Grothey A, Van Cutsem E, Sobrero A, Siena S, Falcone A, Ychou M, Humblet Y, BouchéO, Mineur L, Barone C, Adenis A, Tabernero J, Yoshino T, Lenz HJ, Goldberg RM, Sargent DJ, Cihon F, Cupit L, Wagner A, Laurent D, CORRECT Study Group: Regorafenib monotherapy for previously treated metastatic colorectal cancer (CORRECT): an international, multicentre, randomised, placebo-controlled, phase 3 trial. Lancet. 2013;381(9863): 303.

Hamanishi J, Mandai M, Iwasaki M, Okazaki T, Tanaka Y, Yamaguchi K, et al. Programmed cell death 1 ligand 1 and tumour-infiltrating CD8+ T lymphocytes are prognostic factors of human ovarian cancer. Proc Nat Acad Sci U S A. 2007;104(9):3360-5.

Hodi FS, O'Day SJ, McDermott DF, Weber RW, Sosman JA, Haanan JB, et al. Improved survival with ipilimumab in patients with metastatic melanoma [published erratum appears in N Engl J Med 2010;363(13):1290]. N Engl J Med 2010;363(8):711-23.

Keir ME, Butte MJ, Freeman GJ, Sharpe AH. PD-1 and its ligands in tolerance and immunity. Annu Rev Immunol. 2008;26:677-704.

Khleif S, Lutzky J, Segal N et al. MEDI4736, an anti-PD-L1 antibody with modified Fc domain: preclinical evaluation and early clinical results from a phase I study in patients with advanced solid tumours. (Abst 802) European Cancer Congress 2013, Amsterdam, Netherlands Sept 27-Oct 1, 20

Kirkwood JM, Lorigan P, Hersey P, Hauschild A, Robert C, McDermott D, et al. Phase II trial of tremelimumab (CP-675,206) in patients with advanced refractory or relapsed melanoma. Clin Cancer Res 2010;16(3):1042-8.

Korn EL, Liu PY, Lee SJ, Chapman JA, Niedzwiecki D, Suman VJ, et al. Meta-analysis of phase II cooperative group trials in metastatic stage IV melanoma to determine progression-free and overall survival benchmarks for future phase II trials. J Clin Oncol 2008;26(4):527-34.

Krambeck AE, Dong H, Thompson RH, Kuntz SM, Lohse CM, Leibovich BC, et al. Survivin and B7-H1 are collaborative predictors of survival and represent potential therapeutic targets for patients with renal cell carcinoma. Clin Cancer Res. 2007;13(6):1749-56.

Le DT, Uram JN, Wang H, Bartlett BR, Kemberling H, Eyring AD, et al. PD-1 Blockade in Tumors with Mismatch-Repair Deficiency. N Engl J Med. 2015 Jun 25;372(26):2509-20.

Llosa NJ, Cruise M, Tam A, Wicks EC, Hechenbleikner EM, Taube JM, et al. The vigorous immune microenvironment of microsatellite instable colon cancer is balanced by multiple counter-inhibitory checkpoints. Cancer Discov. 2015 Jan;5(1):43-51.

Loos M, Giese NA, Kleeff J, Giese T, Gaida MM, Bergmann F, et al. Clinical significance and regulation of the costimulatory molecule B7-H1 in pancreatic adenocarcinoma. Cancer Lett. 2008;268(1):98-109.

Mayer RJ, Van Cutsem E, Falcone A, Yoshino T, Garcia-Carbonero R, Mizunuma N, et al. Randomized trial of TAS-102 for refractory metastatic colorectal cancer. N Engl J Med. 2015;372(20):1909-19.

Mu CY, Huang JA, Chen Y, Chen C, Zhang XG. High expression of PD-L1 in lung cancer may contribute to poor prognosis and tumour cells immune escape through suppressing tumour infiltrating dendritic cells maturation. Med Oncol. 2011 Sep;28(3):682-8.

Narwal R, Roskos LK, Robbie GJ (2013) Population Pharmacokinetics of Sifalimumab, an Investigational Anti-Interferonalpha Monoclonal Antibody, in Systemic Lupus Erythematosus. Clin Pharmacokinet 52:1017–1027.

Ng CM, Lum BL, Gimenez V, Kelsey S, Allison D. Rationale for fixed dosing of pertuzumab in cancer patients based on population pharmacokinetic analysis. Pharm Res. 2006 Jun;23(6):1275-84.

Nomi T, Sho M, Akahori T, Hamada K, Kubo A, Kanehiro H, Nakamura S, Enomoto K, Yagita H, Azuma M, Nakajima Y. Clinical significance and therapeutic potential of the programmed death-1 ligand/programmed death-1 pathway in human pancreatic cancer. Clin Cancer Res 2007; 13(7):2151-2157.

Pardoll DM. The blockade of immune checkpoints in cancer immunotherapy. Nat Rev Cancer 2012;12:252-64.

Pardoll D. Immunotherapy: it takes a village. Science. 2014;344(6180):149.

Park JJ, Omiya R, Matsumura Y, Sakoda Y, Kuramasu A, Augustine MM, Yao S, Tsushima F, Narazaki H, Anand S, Liu Y, Strome SE, Chen L, Tamada K. B7-H1/CD80 interaction is required for the induction and maintenance of peripheral T-cell tolerance**.** Blood 2010; 116(8):1291-1298.

Ribas A, Kefford R, Marshall MA, Punt CJA, Haanen JB, Marmol M, et al. Phase III randomized clinical trial comparing tremelimumab with standard-of-care chemotherapy in patients with advanced melanoma. J Clin Oncol. 2013;31;616-22.

Robert C., Thomas L., Bondarenko I., et al. Ipilimumab plus dacarbazine for previously untreated metastatic melanoma. N Engl J Med 2011; 364:2517-2526.

Song M, Chen D, Lu B, Wang C, Zhang J, Huang L, et al. PTEN loss increases PD-L1 protein expression and affects the correlation between PD-L1 expression and clinical parameters in colorectal cancer. PLoS One. 2013 Jun 13;8(6):e65821.

Tarhini AA. Tremelimumab: a review of development to date in solid tumours. Immunotherapy 2013;5:215-29.

Thompson RH, Webster WS, Cheville JC, Lohse CM, Dong H, Leibovich BC, Kuntz SM, Sengupta S, Kwon ED, Blute ML. B7-H1 glycoprotein blockade: A novel strategy to enhance immunotherapy in patients with renal cell carcinoma **.** Urology 2005; 66 (Supplement 5A): 10-14.

Thompson RH, Kwon ED. Significance of B7-H1 Overexpression in Kidney Cancer. Clinical Genitourinary Cancer 2006; 5(3): 206-211.

Venook AP, Niedzwiecki D, Lenz H-J, et al. CALGB/SWOG 80405: Phase III trial of irinotecan/5-FU/leucovorin (FOLFIRI) or oxaliplatin/5-FU/leucovorin (mFOLFOX6) with bevacizumab (BV) or cetuximab (CET) for patients (pts) with KRAS wild-type (wt) untreated metastatic adenocarcinoma of the colon or rectum (MCRC). J Clin Oncol 32: 5s, 2014 (suppl; abstr LBA3.

Wang DD, Zhang S, Zhao H, et al. Fixed dosing versus body size based dosing of monoclonal antibodies in adult clinical trials. J Clin Pharmacol. 2009;49(9):1012–24.

Wang L, Ma Q, Chen X, Guo K, Li J, Zhang M. Clinical Significance of B7-H1 and B7-1 Expressions in Pancreatic Carcinoma. World Journal of Surgery 2010; 34(5): 1059-1065.

Wang Z, Han J, Cui Y, Fan K, Zhou X. Circulating microRNA-21 as noninvasive predictive biomarker for response in cancer immunotherapy. Med Hypotheses 2013;81(1):41-3.

Wolchok JD, Hoos A, O’Day S, Weber JS, Hamid O, Lebbe C, Maio M, Binder M, Bohnsack O, Nichol G, Humphrey R, Hodi FS. Guidelines for the Evaluation of Immune Therapy Activity in Solid Tumors: Immune-Related Response Criteria. Clin Cancer Res 2009;15(23):7412-7420.

Wolchok J.D., Kluger H., Callahan M.K., et al. Nivolumab plus Ipilimumab in Advanced Melanoma. N Engl J Med 2013; 369:122-133.

Zhang S, Shi, R, Li, C, Parivar K, Wang DD. Fixed dosing versus body size-based dosing of therapeutic peptides and proteins in adults. J Clin Pharmacol. 2012Jan;52(1):18-28.

Zou W, Chen L. Inhibitory B7-family molecules in the tumour microenvironment. Nature Reviews Immunology 2008; 8.6:467-477.

APPENDIX I - PERFORMANCE STATUS SCALES/SCORES

PERFORMANCE STATUS CRITERIA

Karnofsky and Lansky performance scores are intended to be multiples of 10.

ECOG (Zubrod)		Karnofsky		Lansky*	
Score	Description	Score	Description	Score	Description
0	Fully active, able to carry on all pre-disease performance without restriction.	100	Normal, no complaints, no evidence of disease.	100	Fully active, normal.
		90	Able to carry on normal activity; minor signs or symptoms of disease.	90	Minor restrictions in physically strenuous activity.
1	Restricted in physically strenuous activity but ambulatory and able to carry out work of a light or sedentary nature, e.g. light housework, office work.	80	Normal activity with effort; some signs or symptoms of disease.	80	Active, but tires more quickly.
		70	Cares for self, unable to carry on normal activity or do active work.	70	Both greater restriction of and less time spent in play activity.
2	Ambulatory and capable of all selfcare but unable to carry out any work activities. Up and about more than 50% of waking hours.	60	Requires occasional assistance, but is able to care for most of his/her needs.	60	Up and around, but minimal active play; keeps busy with quieter activities.
		50	Requires considerable assistance and frequent medical care.	50	Gets dressed, but lies around much of the day; no active play; able to participate in all quiet play and activities.
3	Capable of only limited selfcare; confined to bed or chair more than 50% of waking hours.	40	Disabled, requires special care and assistance.	40	Mostly in bed; participates in quiet activities.
		30	Severely disabled, hospitalization indicated. Death not imminent.	30	In bed; needs assistance even for quiet play.
4	Completely disabled. Cannot carry on any selfcare. Totally confined to bed or chair.	20	Very sick, hospitalization indicated. Death not imminent.	20	Often sleeping; play entirely limited to very passive activities.
		10	Moribund, fatal processes progressing rapidly.	10	No play; does not get out of bed.

* The conversion of the Lansky to ECOG scales is intended for NCI reporting purposes only.

APPENDIX II - DRUG DISTRIBUTION, SUPPLY AND CONTROL

Details of Drug Distribution, Supply and Control/Accountability are provided in the *CO.26 Pharmacy Information Manual*, available on the CO.26 website (http://www.ctg.queensu.ca/trials/gi/CO26/CO26.html).

APPENDIX III - DOSE MODIFICATION AND TOXICITY MANAGEMENT GUIDELINES FOR IMMUNE-MEDIATED, INFUSION RELATED AND NON IMMUNE-MEDIATED REACTIONS
(MEDI4736 Monotherapy or Combination Therapy With Tremelimumab or Tremelimumab Monotherapy) 1Nov2017 Version

Dosing Modification and Toxicity Management Guidelines for Immune-Mediated, Infusion-Related, and Non-Immune–Mediated Reactions (MEDI4736 Monotherapy or Combination Therapy With Tremelimumab or Tremelimumab Monotherapy) 1 November 2017 Version

<table>
<tr><th colspan="2">General Considerations</th></tr>
<tr><th>Dose Modifications</th><th>Toxicity Management</th></tr>
<tr><td>Drug administration modifications of study drug/study regimen will be made to manage potential immune-related AEs based on severity of treatment-emergent toxicities graded per NCI CTCAE v4.03.
In addition to the criteria for permanent discontinuation of study drug/study regimen based on CTC grade/severity (table below), permanently discontinue study drug/study regimen for the following conditions:
• Inability to reduce corticosteroid to a dose of ≤10 mg of prednisone per day (or equivalent) within 12 weeks after last dose of study drug/study regimen
• Recurrence of a previously experienced Grade 3 treatment-related AE following resumption of dosing
Grade 1 No dose modification
Grade 2 Hold study drug/study regimen dose until Grade 2 resolution to Grade ≤1.
If toxicity worsens, then treat as Grade 3 or Grade 4.
Study drug/study regimen can be resumed once event stabilizes to Grade ≤1 after completion of steroid taper.
Patients with endocrinopathies who may require prolonged or continued steroid replacement can be retreated with study drug/study regimen on the following conditions:
1. The event stabilizes and is controlled.
2. The patient is clinically stable as per Investigator or treating physician's clinical judgement.
3. Doses of prednisone are at ≤10 mg/day or equivalent.
Grade 3 Depending on the individual toxicity, study drug/study regimen may be permanently discontinued. Please refer to guidelines below.
Grade 4 Permanently discontinue study drug/study regimen.</td><td>It is recommended that management of immune-mediated adverse events (imAEs) follows the guidelines presented in this table:
– It is possible that events with an inflammatory or immune mediated mechanism could occur in nearly all organs, some of them not noted specifically in these guidelines.
– Whether specific immune-mediated events (and/or laboratory indicators of such events) are noted in these guidelines or not, patients should be thoroughly evaluated to rule out any alternative etiology (e.g., disease progression, concomitant medications, and infections) to a possible immune-mediated event. In the absence of a clear alternative etiology, all such events should be managed as if they were immune related. General recommendations follow.
– Symptomatic and topical therapy should be considered for low-grade (Grade 1 or 2, unless otherwise specified) events.
– For persistent (>3 to 5 days) low-grade (Grade 2) or severe (Grade ≥3) events, promptly start prednisone 1 to 2 mg/kg/day PO or IV equivalent.
– Some events with high likelihood for morbidity and/or mortality – e.g., myo-carditis, or other similar events even if they are not currently noted in the guidelines – should progress rapidly to high dose IV corticosteroids (methylprednisolone at 2 to 4 mg/kg/day) even if the event is Grade 2, and if clinical suspicion is high and/or there has been clinical confirmation. Consider, as necessary, discussing with the study physician, and promptly pursue specialist consultation.
– If symptoms recur or worsen during corticosteroid tapering (28 days of taper), increase the corticosteroid dose (prednisone dose [e.g., up to 2 to 4 mg/kg/day PO or IV equivalent]) until stabilization or improvement of symptoms, then resume corticosteroid tapering at a slower rate (>28 days of taper).
– More potent immunosuppressives such as TNF inhibitors (e.g., infliximab) (also refer to the individual sections of the imAEs for specific type of immunosuppressive) should be considered for events not responding to systemic steroids. Progression to use of more potent immunosuppressives should proceed more rapidly in events with high likelihood for morbidity and/or mortality – e.g., myocarditis, or other similar events even if they are</td></tr>
</table>

Dosing Modification and Toxicity Management Guidelines for Immune-Mediated, Infusion-Related, and Non-Immune–Mediated Reactions (MEDI4736 Monotherapy or Combination Therapy With Tremelimumab or Tremelimumab Monotherapy) 1 November 2017 Version

General Considerations	
**Dose Modifications**	**Toxicity Management**
Note: For Grade ≥3 asymptomatic amylase or lipase levels, hold study drug/study regimen, and if complete work up shows no evidence of pancreatitis, study drug/study regimen may be continued or resumed.<br>Note: Study drug/study regimen should be permanently discontinued in Grade 3 events with high likelihood for morbidity and/or mortality – e.g., myocarditis, or other similar events even if they are not currently noted in the guidelines. Similarly, consider whether study drug/study regimen should be permanently discontinued in Grade 2 events with high likelihood for morbidity and/or mortality – e.g., myocarditis, or other similar events even if they are not currently noted in the guidelines – when they do not rapidly improve to Grade <1 upon treatment with systemic steroids and following full taper<br>Note: There are some exceptions to permanent discontinuation of study drug for Grade 4 events (i.e., hyperthyroidism, hypothyroidism, Type 1 diabetes mellitus).	not currently noted in the guidelines – when these events are not responding to systemic steroids.<br>– With long-term steroid and other immunosuppressive use, consider need for *Pneumocystis jirovecii* pneumonia (PJP, formerly known as *Pneumocystis carinii* pneumonia) prophylaxis, gastrointestinal protection, and glucose monitoring.<br>– Discontinuation of study drug/study regimen is not mandated for Grade 3/Grade 4 inflammatory reactions attributed to local tumor response (e.g., inflammatory reaction at sites of metastatic disease and lymph nodes). Continuation of study drug/study regimen in this situation should be based upon a benefit-risk analysis for that patient.

AE Adverse event; CTC Common Toxicity Criteria; CTCAE Common Terminology Criteria for Adverse Events; imAE immune-mediated adverse event; IV intravenous; NCI National Cancer Institute; PO By mouth.

Pediatric Considerations

Dose Modifications	Toxicity Management
The criteria for permanent discontinuation of study drug/study regimen based on CTC grade/severity is the same for pediatric patients as it is for adult patients, as well as to permanently discontinue study drug/study regimen if unable to reduce corticosteroid ≤ a dose equivalent to that required for corticosteroid replacement therapy **within 12 weeks** after last dose of study drug/study regimen	– All recommendations for specialist consultation should occur with a pediatric specialist in the specialty recommended.<br>– The recommendations for dosing of steroids (i.e., mg/kg/day) and for IV IG and plasmapheresis that are provided for adult patients should also be used for pediatric patients.<br>– The infliximab 5 mg/kg IV dose recommended for adults is the same as recommended for pediatric patients ≥ 6 years old. For dosing in children younger than 6 years old, consult with a pediatric specialist.<br>– For pediatric dosing of mycophenolate mofetil, consult with a pediatric specialist.<br>– With long-term steroid and other immunosuppressive use, consider need for PJP prophylaxis, gastrointestinal protection, and glucose monitoring.

Specific Immune-Mediated Reactions

Adverse Events	Severity Grade of the Event (NCI CTCAE version 4.03)	Dose Modifications	Toxicity Management
**Pneumonitis/Interstitial Lung Disease (ILD)**	**Any Grade**	**General Guidance**	**For Any Grade:**<br>– Monitor patients for signs and symptoms of pneumonitis or ILD (new onset or worsening shortness of breath or cough). Patients should be evaluated with imaging and pulmonary function tests, including other diagnostic procedures as described below.<br>– Initial work-up may include clinical evaluation, monitoring of oxygenation via pulse oximetry (resting and exertion), laboratory work-up, and high- resolution CT scan.
	**Grade 1**<br>(asymptomatic, clinical or diagnostic observations only; intervention not indicated)	No dose modifications required. However, consider holding study drug/study regimen dose as clinically appropriate and during diagnostic work-up for other etiologies.	**For Grade 1 (radiographic changes only):**<br>– Monitor and closely follow up in 2 to 4 days for clinical symptoms, pulse oximetry (resting and exertion), and laboratory work-up and then as clinically indicated.<br>– Consider Pulmonary and Infectious disease consult.
	**Grade 2**<br>(symptomatic; medical intervention indicated; limiting instrumental ADL)	Hold study drug/study regimen dose until Grade 2 resolution to Grade ≤ 1.<br>• If toxicity worsens, then treat as Grade 3 or Grade 4.<br>• If toxicity improves to Grade ≤ 1, then the decision to reinitiate study drug/study regimen will be based upon treating physician's clinical judgment and after completion of steroid taper.	**For Grade 2 (mild to moderate new symptoms):**<br>– Monitor symptoms daily and consider hospitalization.<br>– Promptly start systemic steroids (e.g., prednisone 1 to 2 mg/kg/day PO or IV equivalent).<br>– Reimage as clinically indicated.<br>– If no improvement within 3 to 5 days, additional workup should be considered and prompt treatment with IV methylprednisolone 2 to 4 mg/kg/day started<br>– If still no improvement within 3 to 5 days despite IV methylprednisolone at 2 to 4 mg/kg/day, promptly start immunosuppressive therapy such as TNF inhibitors (e.g., infliximab at 5 mg/kg every 2 weeks). Caution: It is important to rule out sepsis and refer to infliximab label for general guidance before using infliximab.<br>– Once the patient is improving, gradually taper steroids over $\geq$28 days and consider prophylactic antibiotics, antifungals, or

			anti-PJP treatment (refer to current NCCN guidelines for treatment of cancer-related infections [Category 2B recommendation])[a]<br>– Consider pulmonary and infectious disease consult.<br>– Consider, as necessary, discussing with study physician.
	**Grade 3 or 4**<br>(Grade 3: severe symptoms; limiting self-care ADL; oxygen indicated)<br><br>(Grade 4: life-threatening respiratory compromise; urgent intervention indicated [e.g., tracheostomy or intubation])	Permanently discontinue study drug/study regimen.	**For Grade 3 or 4 (severe or new symptoms, new/worsening hypoxia, life-threatening):**<br>– Promptly initiate empiric IV methylprednisolone 1 to 4 mg/kg/day or equivalent.<br>– Obtain Pulmonary and Infectious disease consult; consider, as necessary, discussing with study physician.<br>– Hospitalize the patient.<br>– Supportive care (e.g., oxygen).<br>– If no improvement within 3 to 5 days, additional workup should be considered and prompt treatment with additional immunosuppressive therapy such as TNF inhibitors (e.g., infliximab at 5 mg/kg every 2 weeks' dose) started. Caution: rule out sepsis and refer to infliximab label for general guidance before using infliximab.<br>– Once the patient is improving, gradually taper steroids over ≥28 days and consider prophylactic antibiotics, antifungals, and, in particular, anti-PJP treatment (refer to current NCCN guidelines for treatment of cancer-related infections [Category 2B recommendation]).[a]
**Diarrhea/Colitis**	**Any Grade**	**General Guidance**	**For Any Grade:**<br>– Monitor for symptoms that may be related to diarrhea/enterocolitis (abdominal pain, cramping, or changes in bowel habits such as increased frequency over baseline or blood in stool) or related to bowel perforation (such as sepsis, peritoneal signs, and ileus).<br>– Patients should be thoroughly evaluated to rule out any alternative etiology (e.g., disease progression, other medications, or infections), including testing for clostridium difficile toxin, etc.<br>– Steroids should be considered in the absence of clear alternative etiology, even for low-grade events, in order to prevent potential progression to higher grade event.

		– Use analgesics carefully; they can mask symptoms of perforation and peritonitis.
**Grade 1**<br>(Diarrhea: stool frequency of <4 over baseline per day)<br>(Colitis: asymptomatic; clinical or diagnostic observations only)	No dose modifications.	**For Grade 1:**<br>– Monitor closely for worsening symptoms.<br>– Consider symptomatic treatment, including hydration, electrolyte replacement, dietary changes (e.g., American Dietetic Association colitis diet), and loperamide. Use probiotics as per treating physician's clinical judgment.
**Grade 2**<br>(Diarrhea: stool frequency of 4 to 6 over baseline per day)<br>(Colitis: abdominal pain; mucus or blood in stool)	Hold study drug/study regimen until resolution to Grade ≤1<br>• If toxicity worsens, then treat as Grade 3 or Grade 4.<br>• If toxicity improves to Grade ≤1, then study drug/study regimen can be resumed after completion of steroid taper.	**For Grade 2:**<br>– Consider symptomatic treatment, including hydration, electrolyte replacement, dietary changes (e.g., American Dietetic Association colitis diet), and loperamide and/or budesonide.<br>– Promptly start prednisone 1 to 2 mg/kg/day PO or IV equivalent.<br>– If event is not responsive within 3 to 5 days or worsens despite prednisone at 1 to 2 mg/kg/day PO or IV equivalent, GI consult should be obtained for consideration of further workup, such as imaging and/or colonoscopy, to confirm colitis and rule out perforation, and prompt treatment with IV methylprednisolone 2 to 4 mg/kg/day started.<br>– If still no improvement within 3 to 5 days despite 2 to 4 mg/kg IV methylprednisolone, promptly start immunosuppressives such as infliximab at 5 mg/kg once every 2 weeks[a]. **Caution:** it is important to rule out bowel perforation and refer to infliximab label for general guidance before using infliximab.<br>– Consider, as necessary, discussing with study physician if no resolution to Grade ≤1 in 3 to 4 days.<br>– Once the patient is improving, gradually taper steroids over ≥28 days and consider prophylactic antibiotics, antifungals, and anti-PJP treatment (refer to current NCCN guidelines for treatment of cancer-related infections [Category 2B recommendation]).[a]
**Grade 3 or 4**	**Grade 3**<br>Permanently discontinue study drug/study	**For Grade 3 or 4:**

	(Grade 3 diarrhea: stool frequency of ≥7 over baseline per day; Grade 4 diarrhea: life threatening consequences) (Grade 3 colitis: severe abdominal pain, change in bowel habits, medical intervention indicated, peritoneal signs; Grade 4 colitis: life-threatening consequences, urgent intervention indicated)	regimen for Grade 3 if toxicity does not improve to Grade ≤1 within 14 days; study drug/study regimen can be resumed after completion of steroid taper.<br><br>**Grade 4**<br>Permanently discontinue study drug/study regimen.	– Promptly initiate empiric IV methylprednisolone 2 to 4 mg/kg/day or equivalent.<br>– Monitor stool frequency and volume and maintain hydration.<br>– Urgent GI consult and imaging and/or colonoscopy as appropriate.<br>– If still no improvement within 3 to 5 days of IV methylprednisolone 2 to 4 mg/kg/day or equivalent, promptly start further immunosuppressives (e.g., infliximab at 5 mg/kg once every 2 weeks). **Caution**: Ensure GI consult to rule out bowel perforation and refer to infliximab label for general guidance before using infliximab.<br>– Once the patient is improving, gradually taper steroids over ≥28 days and consider prophylactic antibiotics, antifungals, and anti-PJP treatment (refer to current NCCN guidelines for treatment of cancer-related infections [Category 2B recommendation]).[a]
**Hepatitis (elevated LFTs)**<br>Infliximab should not be used for management of immune-related hepatitis.	**Any Grade**	**General Guidance**	**For Any Grade:**<br>– Monitor and evaluate liver function test: AST, ALT, ALP, and TB.<br>– Evaluate for alternative etiologies (e.g., viral hepatitis, disease progression, concomitant medications).
	**Grade 1**<br>(AST or ALT >ULN and ≤3.0×ULN and/or TB > ULN and ≤1.5×ULN)	• No dose modifications.<br>• If it worsens, then treat as Grade 2 event.	**For Grade 1:**<br>– Continue LFT monitoring per protocol.
	**Grade 2**<br>(AST or ALT >3.0×ULN and ≤5.0×ULN and/or	• Hold study drug/study regimen dose until Grade 2 resolution to Grade ≤1.<br>• If toxicity worsens, then treat as Grade 3 or Grade 4.<br>• If toxicity improves to Grade ≤1 or baseline, resume study drug/study	**For Grade 2:**<br>– Regular and frequent checking of LFTs (e.g., every 1 to 2 days) until elevations of these are improving or resolved.<br>– If no resolution to Grade ≤1 in 1 to 2 days, consider, as necessary, discussing with study physician.

	TB >1.5×ULN and ≤3.0×ULN)	regimen after completion of steroid taper.	– If event is persistent (>3 to 5 days) or worsens, promptly start prednisone 1 to 2 mg/kg/day PO or IV equivalent.<br>– If still no improvement within 3 to 5 days despite 1 to 2 mg/kg/day of prednisone PO or IV equivalent, consider additional work up and start prompt treatment with IV methylprednisolone 2 to 4 mg/kg/day.<br>– If still no improvement within 3 to 5 days despite 2 to 4 mg/kg/day of IV methylprednisolone, promptly start immunosuppressives (i.e., mycophenolate mofetil).[a] Discuss with study physician if mycophenolate mofetil is not available. **Infliximab should NOT be used.**<br>– Once the patient is improving, gradually taper steroids over ≥28 days and consider prophylactic antibiotics, antifungals, and anti-PJP treatment (refer to current NCCN guidelines for treatment of cancer-related infections [Category 2B recommendation]).[a]
	**Grade 3 or 4**<br>(Grade 3: AST or ALT >5.0×ULN and ≤20.0×ULN and/or TB >3.0×ULN and ≤10.0×ULN)<br><br>(Grade 4: AST or ALT >20×ULN and/or TB >10×ULN)	**For Grade 3:**<br>For elevations in transaminases ≤8 × ULN, or elevations in bilirubin ≤5 × ULN:<br>• Hold study drug/study regimen dose until resolution to Grade ≤1 or baseline<br>• Resume study drug/study regimen if elevations downgrade to Grade ≤1 or baseline within 14 days and after completion of steroid taper.<br>• Permanently discontinue study drug/study regimen if the elevations do not downgrade to Grade ≤1 or baseline within 14 days<br>For elevations in transaminases >8 × ULN or elevations in bilirubin >5 × ULN, discontinue study drug/study regimen.<br>Permanently discontinue study drug/study regimen for any case meeting Hy's law criteria (AST and/or ALT >3 × ULN +	**For Grade 3 or 4:**<br>– Promptly initiate empiric IV methylprednisolone at 1 to 4 mg/kg/day or equivalent.<br>– If still no improvement within 3 to 5 days despite 1 to 4 mg/kg/day methylprednisolone IV or equivalent, promptly start treatment with immunosuppressive therapy (i.e., mycophenolate mofetil). Discuss with study physician if mycophenolate is not available. **Infliximab should NOT be used.**<br>– Perform hepatology consult, abdominal workup, and imaging as appropriate.<br>– Once the patient is improving, gradually taper steroids over ≥28 days and consider prophylactic antibiotics, antifungals, and anti-PJP treatment (refer to current NCCN guidelines for treatment of cancer-related infections [Category 2B recommendation]).[a]

		bilirubin >2 × ULN without initial findings of cholestasis (i.e., elevated alkaline P04) and in the absence of any alternative cause.[b]<br><br>**For Grade 4:**<br>Permanently discontinue study drug/study regimen.	
**Nephritis or renal dysfunction**<br>(elevated serum creatinine)	**Any Grade**	**General Guidance**	**For Any Grade:**<br>– Consult with nephrologist.<br>– Monitor for signs and symptoms that may be related to changes in renal function (e.g., routine urinalysis, elevated serum BUN and creatinine, decreased creatinine clearance, electrolyte imbalance, decrease in urine output, or proteinuria).<br>– Patients should be thoroughly evaluated to rule out any alternative etiology (e.g., disease progression or infections).<br>– Steroids should be considered in the absence of clear alternative etiology even for low-grade events (Grade 2), in order to prevent potential progression to higher grade event.
	**Grade 1**<br>(Serum creatinine > 1 to 1.5 × baseline; > ULN to 1.5 × ULN)	No dose modifications.	**For Grade 1:**<br>– Monitor serum creatinine weekly and any accompanying symptoms.<br>• If creatinine returns to baseline, resume its regular monitoring per study protocol.<br>• If creatinine worsens, depending on the severity, treat as Grade 2, 3, or 4.<br>– Consider symptomatic treatment, including hydration, electrolyte replacement, and diuretics.
	**Grade 2**	Hold study drug/study regimen until resolution to Grade ≤1 or baseline.	**For Grade 2:**<br>– Consider symptomatic treatment, including hydration, electrolyte replacement, and diuretics.

	(serum creatinine >1.5 to 3.0 × baseline; >1.5 to 3.0 × ULN)	• If toxicity worsens, then treat as Grade 3 or 4.<br>• If toxicity improves to Grade ≤1 or baseline, then resume study drug/study regimen after completion of steroid taper.	– Carefully monitor serum creatinine every 2 to 3 days and as clinically warranted.<br>– Consult nephrologist and consider renal biopsy if clinically indicated.<br>– If event is persistent (>3 to 5 days) or worsens, promptly start prednisone 1 to 2 mg/kg/day PO or IV equivalent.<br>– If event is not responsive within 3 to 5 days or worsens despite prednisone at 1 to 2 mg/kg/day PO or IV equivalent, additional workup should be considered and prompt treatment with IV methylprednisolone at 2 to 4 mg/kg/day started.<br>– Once the patient is improving, gradually taper steroids over ≥28 days and consider prophylactic antibiotics, antifungals, and anti-PJP treatment (refer to current NCCN guidelines for treatment of cancer-related infections [Category 2B recommendation]).[a]<br>– When event returns to baseline, resume study drug/study regimen and routine serum creatinine monitoring per study protocol.
	**Grade 3 or 4**<br>(Grade 3: serum creatinine >3.0 × baseline; >3.0 to 6.0 × ULN;<br><br>Grade 4: serum creatinine >6.0 × ULN)	Permanently discontinue study drug/study regimen.	**For Grade 3 or 4:**<br>– Carefully monitor serum creatinine on daily basis.<br>– Consult nephrologist and consider renal biopsy if clinically indicated.<br>– Promptly start prednisone 1 to 2 mg/kg/day PO or IV equivalent.<br>– If event is not responsive within 3 to 5 days or worsens despite prednisone at 1 to 2 mg/kg/day PO or IV equivalent, additional workup should be considered and prompt treatment with IV methylprednisolone 2 to 4 mg/kg/day started.<br>– Once the patient is improving, gradually taper steroids over ≥28 days and consider prophylactic antibiotics, antifungals, and anti-PJP treatment (refer to current NCCN guidelines for treatment of cancer-related infections [Category 2B recommendation]).[a]
**Rash**	**Any Grade**<br>(refer to NCI CTCAE v 4.03 for definition of	**General Guidance**	**For Any Grade:**<br>– Monitor for signs and symptoms of dermatitis (rash and pruritus).

(excluding bullous skin formations)	severity/grade depending on type of skin rash)		– IF THERE IS ANY BULLOUS FORMATION, THE STUDY PHYSICIAN SHOULD BE CONTACTED **AND STUDY DRUG DISCONTINUED.**
	**Grade 1**	No dose modifications.	**For Grade 1:**<br>– Consider symptomatic treatment, including oral antipruritics (e.g., diphenhydramine or hydroxyzine) and topical therapy (e.g., urea cream).
	**Grade 2**	For persistent (>1 to 2 weeks) Grade 2 events, hold scheduled study drug/study regimen until resolution to Grade ≤1 or baseline.<br>• If toxicity worsens, then treat as Grade 3.<br>• If toxicity improves to Grade ≤1 or baseline, then resume drug/study regimen after completion of steroid taper.	**For Grade 2:**<br>– Obtain dermatology consult.<br>– Consider symptomatic treatment, including oral antipruritics (e.g., diphenhydramine or hydroxyzine) and topical therapy (e.g., urea cream).<br>– Consider moderate-strength topical steroid.<br>– If no improvement of rash/skin lesions occurs within 3 to 5 days or is worsening despite symptomatic treatment and/or use of moderate strength topical steroid, consider, as necessary, discussing with study physician and promptly start systemic steroids such as prednisone 1 to 2 mg/kg/day PO or IV equivalent.<br>– Consider skin biopsy if the event is persistent for >1 to 2 weeks or recurs.
	**Grade 3 or 4**	**For Grade 3:**<br>Hold study drug/study regimen until resolution to Grade ≤1 or baseline.<br>If temporarily holding the study drug/study regimen does not provide improvement of the Grade 3 skin rash to Grade ≤1 or baseline within 30 days, then permanently discontinue study drug/study regimen.<br>**For Grade 4:**	**For Grade 3 or 4:**<br>– Consult dermatology.<br>– Promptly initiate empiric IV methylprednisolone 1 to 4 mg/kg/day or equivalent.<br>– Consider hospitalization.<br>– Monitor extent of rash [Rule of Nines].<br>– Consider skin biopsy (preferably more than 1) as clinically feasible.<br>– Once the patient is improving, gradually taper steroids over ≥28 days and consider prophylactic antibiotics, antifungals, and anti-PJP treatment (refer to current NCCN guidelines for treatment of cancer-related infections [Category 2B recommendation]).[a]

		Permanently discontinue study drug/study regimen.	– Consider, as necessary, discussing with study physician.
**Endocrinopathy**<br>(e.g., hyperthyroidism, hypothyroidism, Type 1 diabetes mellitus, hypophysitis, hypopituitarism, and adrenal insufficiency; exocrine event of amylase/lipase increased also included in this section)	**Any Grade**<br>(depending on the type of endocrinopathy, refer to NCI CTCAE v4.03 for defining the CTC grade/severity)	**General Guidance**	**For Any Grade:**<br>– Consider consulting an endocrinologist for endocrine events.<br>– Consider, as necessary, discussing with study physician.<br>– Monitor patients for signs and symptoms of endocrinopathies. Non-specific symptoms include headache, fatigue, behavior changes, changed mental status, vertigo, abdominal pain, unusual bowel habits, polydipsia, polyuria, hypotension, and weakness.<br>– Patients should be thoroughly evaluated to rule out any alternative etiology (e.g., disease progression including brain metastases, or infections).<br>– Depending on the suspected endocrinopathy, monitor and evaluate thyroid function tests: TSH, free T3 and free T4 and other relevant endocrine and related labs (e.g., blood glucose and ketone levels, HgA1c).<br>– For modest asymptomatic elevations in serum amylase and lipase, corticosteroid treatment is not indicated as long as there are no other signs or symptoms of pancreatic inflammation.<br>– If a patient experiences an AE that is thought to be possibly of autoimmune nature (e.g., thyroiditis, pancreatitis, hypophysitis, or diabetes insipidus), the investigator should send a blood sample for appropriate autoimmune antibody testing.
	**Grade 1**	No dose modifications.	**For Grade 1 (including those with asymptomatic TSH elevation):**<br>– Monitor patient with appropriate endocrine function tests.<br>– For suspected hypophysitis/hypopituitarism, consider consultation of an endocrinologist to guide assessment of early-morning ACTH, cortisol, TSH and free T4; also consider gonadotropins, sex hormones, and prolactin levels, as well as cosyntropin stimulation test (though it may not be useful in diagnosing early secondary adrenal insufficiency).<br>– If TSH < 0.5 × LLN, or TSH >2 × ULN, or consistently out of range in 2 subsequent measurements, include free T4 at subsequent cycles as clinically indicated and consider consultation of an endocrinologist.

**Grade 2**	For Grade 2 endocrinopathy other than hypothyroidism and Type 1 diabetes mellitus, hold study drug/study regimen dose until patient is clinically stable.<br>• If toxicity worsens, then treat as Grade 3 or Grade 4.<br>Study drug/study regimen can be resumed once event stabilizes and after completion of steroid taper.<br>Patients with endocrinopathies who may require prolonged or continued steroid replacement (e.g., adrenal insufficiency) can be retreated with study drug/study regimen on the following conditions:<br>1. The event stabilizes and is controlled.<br>2. The patient is clinically stable as per investigator or treating physician's clinical judgement.<br>3. Doses of prednisone are ≤10 mg/day or equivalent.	**For Grade 2 (including those with symptomatic endocrinopathy):**<br>– Consult endocrinologist to guide evaluation of endocrine function and, as indicated by suspected endocrinopathy and as clinically indicated, consider pituitary scan.<br>– For all patients with abnormal endocrine work up, except those with isolated hypothyroidism or Type 1 DM, and as guided by an endocrinologist, consider short-term corticosteroids (e.g., 1 to 2 mg/kg/day methylprednisolone or IV equivalent) and prompt initiation of treatment with relevant hormone replacement (e.g., hydrocortisone, sex hormones).<br>– Isolated hypothyroidism may be treated with replacement therapy, without study drug/study regimen interruption, and without corticosteroids.<br>– Isolated Type 1 diabetes mellitus (DM) may be treated with appropriate diabetic therapy, without study drug/study regimen interruption, and without corticosteroids.<br>– Once patients on steroids are improving, gradually taper immunosuppressive steroids (as appropriate and with guidance of endocrinologist) over ≥28 days and consider prophylactic antibiotics, antifungals, and anti-PJP treatment (refer to current NCCN guidelines for treatment of cancer-related infections [Category 2B recommendation]).[a]<br>– For patients with normal endocrine workup (laboratory assessment or MRI scans), repeat laboratory assessments/MRI as clinically indicated.
**Grade 3 or 4**	For Grade 3 or 4 endocrinopathy other than hypothyroidism and Type 1 diabetes mellitus, hold study drug/study regimen dose until endocrinopathy symptom(s) are controlled.<br>Study drug/study regimen can be resumed once event stabilizes and after completion of steroid taper.<br>Patients with endocrinopathies who may require prolonged or continued steroid replacement (e.g., adrenal insufficiency)	**For Grade 3 or 4:**<br>– Consult endocrinologist to guide evaluation of endocrine function and, as indicated by suspected endocrinopathy and as clinically indicated, consider pituitary scan. Hospitalization recommended.<br>– For all patients with abnormal endocrine work up, except those with isolated hypothyroidism or Type 1 DM, and as guided by an endocrinologist, promptly initiate empiric IV methylprednisolone 1 to 2 mg/kg/day or equivalent, as well as relevant hormone replacement (e.g., hydrocortisone, sex hormones).<br>– For adrenal crisis, severe dehydration, hypotension, or shock, immediately initiate IV corticosteroids with mineralocorticoid activity.

		can be retreated with study drug/study regimen on the following conditions:<br>1. The event stabilizes and is controlled.<br>2. The patient is clinically stable as per investigator or treating physician's clinical judgement.<br>3. Doses of prednisone are ≤10 mg/day or equivalent.	– Isolated hypothyroidism may be treated with replacement therapy, without study drug/study regimen interruption, and without corticosteroids.<br>– Isolated Type 1 diabetes mellitus may be treated with appropriate diabetic therapy, without study drug/study regimen interruption, and without corticosteroids.<br>– Once patients on steroids are improving, gradually taper immunosuppressive steroids (as appropriate and with guidance of endocrinologist) over ≥28 days and consider prophylactic antibiotics, antifungals, and anti-PJP treatment (refer to current NCCN guidelines for treatment of cancer-related infections [Category 2B recommendation]).[a]
**Neurotoxicity**<br>(to include but not be limited to limbic encephalitis and autonomic neuropathy, excluding Myasthenia Gravis and Guillain-Barre)	**Any Grade**<br>(depending on the type of neurotoxicity, refer to NCI CTCAE v4.03 for defining the CTC grade/severity)	**General Guidance**	**For Any Grade:**<br>– Patients should be evaluated to rule out any alternative etiology (e.g., disease progression, infections, metabolic syndromes, or medications).<br>– Monitor patient for general symptoms (headache, nausea, vertigo, behavior change, or weakness).<br>– Consider appropriate diagnostic testing (e.g., electromyogram and nerve conduction investigations).<br>– Perform symptomatic treatment with neurological consult as appropriate.<br>–
	**Grade 1**	No dose modifications.	**For Grade 1:**<br>– See "Any Grade" recommendations above.
	**Grade 2**	For acute motor neuropathies or neurotoxicity, hold study drug/study regimen dose until resolution to Grade ≤1.<br>For sensory neuropathy/neuropathic pain, consider holding study drug/study regimen dose until resolution to Grade ≤1.<br>If toxicity worsens, then treat as Grade 3 or 4.	**For Grade 2:**<br>– Consider, as necessary, discussing with the study physician.<br>– Obtain neurology consult.<br>– Sensory neuropathy/neuropathic pain may be managed by appropriate medications (e.g., gabapentin or duloxetine).<br>– Promptly start systemic steroids prednisone 1 to 2 mg/kg/day PO or IV equivalent.<br>– If no improvement within 3 to 5 days despite 1 to 2 mg/kg/day prednisone PO or IV equivalent, consider additional workup and promptly treat with additional immunosuppressive therapy (e.g., IV IG).

		Study drug/study regimen can be resumed once event improves to Grade ≤1 and after completion of steroid taper.	
	**Grade 3 or 4**	**For Grade 3:**<br>Hold study drug/study regimen dose until resolution to Grade ≤1.<br>Permanently discontinue study drug/study regimen if Grade 3 imAE does not resolve to Grade ≤1 within 30 days.<br>**For Grade 4:**<br>Permanently discontinue study drug/study regimen.	**For Grade 3 or 4:**<br>– Consider, as necessary, discussing with study physician.<br>– Obtain neurology consult.<br>– Consider hospitalization.<br>– Promptly initiate empiric IV methylprednisolone 1 to 2 mg/kg/day or equivalent.<br>– If no improvement within 3 to 5 days despite IV corticosteroids, consider additional workup and promptly treat with additional immunosuppressants (e.g., IV IG).<br>– Once stable, gradually taper steroids over ≥28 days.
**Peripheral neuromotor syndromes**<br>(such as Guillain-Barre and myasthenia gravis)	**Any Grade**	**General Guidance**	**For Any Grade:**<br>– The prompt diagnosis of immune-mediated peripheral neuromotor syndromes is important, since certain patients may unpredictably experience acute decompensations that can result in substantial morbidity or in the worst case, death. Special care should be taken for certain sentinel symptoms that may predict a more severe outcome, such as prominent dysphagia, rapidly progressive weakness, and signs of respiratory insufficiency or autonomic instability.<br>– Patients should be evaluated to rule out any alternative etiology (e.g., disease progression, infections, metabolic syndromes or medications). It should be noted that the diagnosis of immune-mediated peripheral neuromotor syndromes can be particularly challenging in patients with underlying cancer, due to the multiple potential confounding effects of cancer (and its treatments) throughout the neuraxis. Given the importance of prompt and accurate diagnosis, it is essential to have a low threshold to obtain a neurological consult.<br>– Neurophysiologic diagnostic testing (e.g., electromyogram and nerve conduction investigations, and "repetitive stimulation" if myasthenia is suspected) are routinely indicated upon suspicion of such conditions and may be best facilitated by means of a neurology consultation.

		– It is important to consider that the use of steroids as the primary treatment of Guillain-Barre is not typically considered effective. Patients requiring treatment should be started with IV IG and followed by plasmapheresis if not responsive to IV IG.
**Grade 1**	No dose modifications.	**For Grade 1:**<br>– Consider, as necessary, discussing with the study physician.<br>– Care should be taken to monitor patients for sentinel symptoms of a potential decompensation as described above.<br>– Obtain a neurology consult.
**Grade 2**	Hold study drug/study regimen dose until resolution to Grade ≤1.<br>Permanently discontinue study drug/study regimen if it does not resolve to Grade ≤1 within 30 days or if there are signs of respiratory insufficiency or autonomic instability.	**For Grade 2:**<br>– Consider, as necessary, discussing with the study physician.<br>– Care should be taken to monitor patients for sentinel symptoms of a potential decompensation as described above.<br>– Obtain a neurology consult<br>– Sensory neuropathy/neuropathic pain may be managed by appropriate medications (e.g., gabapentin or duloxetine).<br>*MYASTHENIA GRAVIS:*<br>○ Steroids may be successfully used to treat myasthenia gravis. It is important to consider that steroid therapy (especially with high doses) may result in transient worsening of myasthenia and should typically be administered in a monitored setting under supervision of a consulting neurologist.<br>○ Patients unable to tolerate steroids may be candidates for treatment with plasmapheresis or IV IG. Such decisions are best made in consultation with a neurologist, taking into account the unique needs of each patient.<br>○ If myasthenia gravis-like neurotoxicity is present, consider starting AChE inhibitor therapy in addition to steroids. Such therapy, if successful, can also serve to reinforce the diagnosis.<br>*GUILLAIN-BARRE:*<br>○ It is important to consider here that the use of steroids as the primary treatment of Guillain-Barre is not typically considered effective.

			○ Patients requiring treatment should be started with IV IG and followed by plasmapheresis if not responsive to IV IG.
	**Grade 3 or 4**	**For Grade 3:**<br>Hold study drug/study regimen dose until resolution to Grade ≤1.<br>Permanently discontinue study drug/study regimen if Grade 3 imAE does not resolve to Grade ≤1 within 30 days or if there are signs of respiratory insufficiency or autonomic instability.<br><br>**For Grade 4:**<br>Permanently discontinue study drug/study regimen.	**For Grade 3 or 4 (severe or life-threatening events):**<br>– Consider, as necessary, discussing with study physician.<br>– Recommend hospitalization.<br>– Monitor symptoms and obtain neurological consult.<br>*MYASTHENIA GRAVIS:*<br>○ Steroids may be successfully used to treat myasthenia gravis. They should typically be administered in a monitored setting under supervision of a consulting neurologist.<br>○ Patients unable to tolerate steroids may be candidates for treatment with plasmapheresis or IV IG.<br>○ If myasthenia gravis-like neurotoxicity present, consider starting AChE inhibitor therapy in addition to steroids. Such therapy, if successful, can also serve to reinforce the diagnosis.<br>*GUILLAIN-BARRE:*<br>○ It is important to consider here that the use of steroids as the primary treatment of Guillain-Barre is not typically considered effective.<br>○ Patients requiring treatment should be started with IV IG and followed by plasmapheresis if not responsive to IV IG.
**Myocarditis**	**Any Grade**	**General Guidance**<br>Discontinue drug permanently if biopsy-proven immune-mediated myocarditis.	**For Any Grade:**<br>– The prompt diagnosis of immune-mediated myocarditis is important, particularly in patients with baseline cardiopulmonary disease and reduced cardiac function.<br>– Consider, as necessary, discussing with the study physician.<br>– Monitor patients for signs and symptoms of myocarditis (new onset or worsening chest pain, arrhythmia, shortness of breath, peripheral edema). As some symptoms can overlap with lung toxicities, simultaneously evaluate for and rule out pulmonary toxicity as well as other causes (e.g., pulmonary embolism, congestive heart failure, malignant pericardial effusion). A

		Cardiology consultation should be obtained early, with prompt assessment of whether and when to complete a cardiac biopsy, including any other diagnostic procedures.<br>– Initial work-up should include clinical evaluation, BNP, cardiac enzymes, ECG, echocardiogram (ECHO), monitoring of oxygenation via pulse oximetry (resting and exertion), and additional laboratory work-up as indicated. Spiral CT or cardiac MRI can complement ECHO to assess wall motion abnormalities when needed.<br>– Patients should be thoroughly evaluated to rule out any alternative etiology (e.g., disease progression, other medications, or infections)
**Grade 1**<br>(asymptomatic with laboratory (e.g., BNP) or cardiac imaging abnormalities)	No dose modifications required unless clinical suspicion is high, in which case hold study drug/study regimen dose during diagnostic work-up for other etiologies. If study drug/study regimen is held, resume after complete resolution to Grade 0.	**For Grade 1 (no definitive findings):**<br>- Monitor and closely follow up in 2 to 4 days for clinical symptoms, BNP, cardiac enzymes, ECG, ECHO, pulse oximetry (resting and exertion), and laboratory work-up as clinically indicated.<br>- Consider using steroids if clinical suspicion is high.
**Grade 2, 3 or 4**<br>(Grade 2: Symptoms with mild to moderate activity or exertion)<br>(Grade 3: Severe with symptoms at rest or with minimal activity or exertion; intervention indicated)<br>(Grade 4: Life-threatening consequences; urgent intervention indicated (e.g., continuous IV	- If Grade 2 -- Hold study drug/study regimen dose until resolution to Grade 0. If toxicity rapidly improves to Grade 0, then the decision to reinitiate study drug/study regimen will be based upon treating physician's clinical judgment and after completion of steroid taper. If toxicity does not rapidly improve, permanently. discontinue study drug/study regimen.<br>If Grade 3-4, permanently discontinue study drug/study regimen.	**For Grade 2-4:**<br>– Monitor symptoms daily, hospitalize.<br>– Promptly start IV methylprednisolone 2 to 4 mg/kg/day or equivalent after Cardiology consultation has determined whether and when to complete diagnostic procedures including a cardiac biopsy.<br>– Supportive care (e.g., oxygen).<br>– If no improvement within 3 to 5 days despite IV methylprednisolone at 2 to 4 mg/kg/day, promptly start immunosuppressive therapy such as TNF inhibitors (e.g., infliximab at 5 mg/kg every 2 weeks). Caution: It is important to rule out sepsis and refer to infliximab label for general guidance before using infliximab.<br>– Once the patient is improving, gradually taper steroids over ≥28 days and consider prophylactic antibiotics, antifungals, or anti-PJP treatment (refer to current NCCN guidelines for treatment of cancer-related infections [Category 2B recommendation]).[a]

	therapy or mechanical hemodynamic support))		
**Myositis/Polymyositis (“Poly/myositis”)**	**Any Grade**	**General Guidance**	**For Any Grade:**<br>– Monitor patients for signs and symptoms of poly/myositis. Typically, muscle weakness/pain occurs in proximal muscles including upper arms, thighs, shoulders, hips, neck and back, but rarely affects the extremities including hands and fingers; also difficulty breathing and/or trouble swallowing can occur and progress rapidly. Increased general feelings of tiredness and fatigue may occur, and there can be new-onset falling, difficulty getting up from a fall, and trouble climbing stairs, standing up from a seated position, and/or reaching up.<br>– If poly/myositis is suspected, a Neurology consultation should be obtained early, with prompt guidance on diagnostic procedures. Myocarditis may co-occur with poly/myositis; refer to guidance under Myocarditis. Given breathing complications, refer to guidance under Pneumonitis/ILD. Given possibility of an existent (but previously unknown) autoimmune disorder, consider Rheumatology consultation.<br>– Consider, as necessary, discussing with the study physician.<br>– Initial work-up should include clinical evaluation, creatine kinase, aldolase, LDH, BUN/creatinine, erythrocyte sedimentation rate or C-reactive protein level, urine myoglobin, and additional laboratory work-up as indicated, including a number of possible rheumatological/antibody tests (i.e., consider whether a rheumatologist consultation is indicated and could guide need for rheumatoid factor, antinuclear antibody, anti-smooth muscle, antisynthetase [such as anti-Jo-1], and/or signal-recognition particle antibodies). Confirmatory testing may include electromyography, nerve conduction studies, MRI of the muscles, and/or a muscle biopsy. Consider Barium swallow for evaluation of dysphagia or dysphonia.<br>Patients should be thoroughly evaluated to rule out any alternative etiology (e.g., disease progression, other medications, or infections).
	**Grade 1**<br>(mild pain)	- No dose modifications.	**For Grade 1:**<br>– Monitor and closely follow up in 2 to 4 days for clinical symptoms and initiate evaluation as clinically indicated.

		– Consider Neurology consult.<br>– Consider, as necessary, discussing with the study physician.
**Grade 2**<br>(moderate pain associated with weakness; pain limiting instrumental activities of daily living [ADLs])	Hold study drug/study regimen dose until resolution to Grade ≤1.<br>- Permanently discontinue study drug/study regimen if it does not resolve to Grade ≤1 within 30 days or if there are signs of respiratory insufficiency.	**For Grade 2:**<br>– Monitor symptoms daily and consider hospitalization.<br>– Obtain Neurology consult, and initiate evaluation.<br>– Consider, as necessary, discussing with the study physician.<br>– If clinical course is rapidly progressive (particularly if difficulty breathing and/or trouble swallowing), promptly start IV methylprednisolone 2 to 4 mg/kg/day systemic steroids along with receiving input from Neurology consultant<br>– If clinical course is *not* rapidly progressive, start systemic steroids (e.g., prednisone 1 to 2 mg/kg/day PO or IV equivalent); if no improvement within 3 to 5 days, continue additional work up and start treatment with IV methylprednisolone 2 to 4 mg/kg/day<br>– If after start of IV methylprednisolone at 2 to 4 mg/kg/day there is no improvement within 3 to 5 days, consider start of immunosuppressive therapy such as TNF inhibitors (e.g., infliximab at 5 mg/kg every 2 weeks). Caution: It is important to rule out sepsis and refer to infliximab label for general guidance before using infliximab.<br>– Once the patient is improving, gradually taper steroids over ≥28 days and consider prophylactic antibiotics, antifungals, or anti-PJP treatment (refer to current NCCN guidelines for treatment of cancer-related infections [Category 2B recommendation]).[a]
**Grade 3 or 4**<br>(pain associated with severe weakness; limiting self-care ADLs)	**For Grade 3:**<br>Hold study drug/study regimen dose until resolution to Grade ≤1.<br>Permanently discontinue study drug/study regimen if Grade 3 imAE does not resolve to Grade ≤1 within 30 days or if there are signs of respiratory insufficiency.<br>**For Grade 4:**	**For Grade 3 or 4 (severe or life-threatening events):**<br>– Monitor symptoms closely; recommend hospitalization.<br>– Obtain Neurology consult, and complete full evaluation.<br>– Consider, as necessary, discussing with the study physician.<br>– Promptly start IV methylprednisolone 2 to 4 mg/kg/day systemic steroids along with receiving input from Neurology consultant.<br>– If after start of IV methylprednisolone at 2 to 4 mg/kg/day there is no improvement within 3 to 5 days, consider start of immunosuppressive therapy such as TNF inhibitors (e.g., infliximab at 5 mg/kg every 2 weeks). Caution: It is important to rule out sepsis and refer to infliximab label for general guidance before using infliximab.

	- Permanently discontinue study drug/study regimen.	– Consider whether patient may require IV IG, plasmapheresis.<br>– Once the patient is improving, gradually taper steroids over ≥28 days and consider prophylactic antibiotics, antifungals, or anti-PJP treatment (refer to current NCCN guidelines for treatment of cancer-related infections [Category 2B recommendation]).[a]

[a]ASCO Educational Book 2015 "Managing Immune Checkpoint Blocking Antibody Side Effects" by Michael Postow MD.

[b]FDA Liver Guidance Document 2009 Guidance for Industry: Drug Induced Liver Injury – Premarketing Clinical Evaluation.

AChE Acetylcholine esterase; ADL Activities of daily living; AE Adverse event; ALP Alkaline phosphatase test; ALT Alanine aminotransferase; AST Aspartate aminotransferase; BUN Blood urea nitrogen; CT Computed tomography; CTCAE Common Terminology Criteria for Adverse Events; ILD Interstitial lung disease; imAE immune-mediated adverse event; IG Immunoglobulin; IV Intravenous; GI Gastrointestinal; LFT Liver function tests; LLN Lower limit of normal; MRI Magnetic resonance imaging; NCI National Cancer Institute; NCCN National Comprehensive Cancer Network; PJP *Pneumocystis jirovecii* pneumonia (formerly known as *Pneumocystis carinii* pneumonia); PO By mouth; T3 Triiodothyronine; T4 Thyroxine; TB Total bilirubin; TNF Tumor necrosis factor; TSH Thyroid-stimulating hormone; ULN Upper limit of normal.

Infusion-Related Reactions

Severity Grade of the Event (NCI CTCAE version 4.03)	Dose Modifications	Toxicity Management
**Any Grade**	General Guidance	**For Any Grade:**<br>– Manage per institutional standard at the discretion of investigator.<br>– Monitor patients for signs and symptoms of infusion-related reactions (e.g., fever and/or shaking chills, flushing and/or itching, alterations in heart rate and blood pressure, dyspnea or chest discomfort, or skin rashes) and anaphylaxis (e.g., generalized urticaria, angioedema, wheezing, hypotension, or tachycardia).
**Grade 1 or 2**	**For Grade 1:**<br>The infusion rate of study drug/study regimen may be decreased by 50% or temporarily interrupted until resolution of the event.<br>**For Grade 2:**<br>The infusion rate of study drug/study regimen may be decreased 50% or temporarily interrupted until resolution of the event.<br>Subsequent infusions may be given at 50% of the initial infusion rate.	**For Grade 1 or 2:**<br>– Acetaminophen and/or antihistamines may be administered per institutional standard at the discretion of the investigator.<br>– Consider premedication per institutional standard prior to subsequent doses.<br>– Steroids should not be used for routine premedication of Grade ≤2 infusion reactions.
**Grade 3 or 4**	**For Grade 3 or 4:**<br>Permanently discontinue study drug/study regimen.	**For Grade 3 or 4:**<br>– Manage severe infusion-related reactions per institutional standards (e.g., IM epinephrine, followed by IV diphenhydramine and ranitidine, and IV glucocorticoid).

CTCAE Common Terminology Criteria for Adverse Events; IM intramuscular; IV intravenous; NCI National Cancer Institute.

Non–Immune-Mediated Reactions

Severity Grade of the Event (NCI CTCAE version 4.03)	Dose Modifications	Toxicity Management
**Any Grade**	Note: Dose modifications are not required for AEs not deemed to be related to study treatment (i.e., events due to underlying disease) or for laboratory abnormalities not deemed to be clinically significant.	Treat accordingly, as per institutional standard.
**Grade 1**	No dose modifications.	Treat accordingly, as per institutional standard.
**Grade 2**	Hold study drug/study regimen until resolution to ≤Grade 1 or baseline.	Treat accordingly, as per institutional standard.
**Grade 3**	Hold study drug/study regimen until resolution to ≤Grade 1 or baseline.<br>For AEs that downgrade to ≤Grade 2 within 7 days or resolve to ≤Grade 1 or baseline within 14 days, resume study drug/study regimen administration. Otherwise, discontinue study drug/study regimen.	Treat accordingly, as per institutional standard.
**Grade 4**	Discontinue study drug/study regimen (Note: For Grade 4 labs, decision to discontinue should be based on accompanying clinical signs/symptoms, the Investigator's clinical judgment, and consultation with the Sponsor.).	Treat accordingly, as per institutional standard.

Note: As applicable, for early phase studies, the following sentence may be added: "Any event greater than or equal to Grade 2, please discuss with Study Physician."

AE Adverse event; CTCAE Common Terminology Criteria for Adverse Events; NCI National Cancer Institute.

APPENDIX IV - DOCUMENTATION FOR STUDY

Follow-up is required for patients from the time of randomization and will apply to all eligible patients.

This trial will use a web-based Electronic Data Capture (EDC) system for all data collection. For details of accessing the EDC system and completing the on-line Case Report Forms please refer to the "CCTG EDC Generic Data Management Guidebook" posted on the CO.26 area of the CCTG web-site (www.ctg.queensu.ca).

The electronic CRFs to be used in this trial, through the EDC system, are as follows:

<table>
<tr><th>Electronic Folder</th><th>Timing</th><th>To be completed electronically</th><th>Supporting Documentation Required[1]</th></tr>
<tr><td>Eligibility Checklist</td><td></td><td>At the time of randomization</td><td rowspan="2">• consent form[2]
• relevant pathology report(s)
• RAS reports
• relevant operative report(s)
• relevant radiology reports (including CT/MRI abdomen/pelvis, CT/MRI chest, chest x-ray)
• tumour measurement worksheet
• ECG report</td></tr>
<tr><td>Baseline Report</td><td></td><td>Within 2 weeks of randomization</td></tr>
<tr><td rowspan="3">Correlative Studies Report (Tumour and Blood)</td><td rowspan="3">Continuous running-log folder

See Section 5.0</td><td>Information pertaining to tumour tissue submission must be completed as soon as possible after randomization, and tissue submitted within 4 weeks of randomization.</td><td rowspan="3">• Consent form[2]
• Diagnostic pathology report (for tumour tissue only)</td></tr>
<tr><td>Information pertaining to <u>baseline/pretreatment</u> blood collection for correlative studies (i.e. whole blood, plasma, serum) must be completed within 2 weeks of randomization.</td></tr>
<tr><td>Information pertaining to <u>post randomization</u> blood collection samples (i.e. whole blood, plasma, serum) for correlative studies and banking should be completed within 2 weeks after collection of blood specimens.</td></tr>
<tr><td>Concomitant Medication Report</td><td>Continuous running-log folder</td><td></td><td></td></tr>
<tr><td>Best Supportive Care Report (Arm 1 only)</td><td>Every 4 weeks (28 days) until objective progression</td><td>Within 2 weeks of the end of each reporting period</td><td>• If available/applicable:
• CT/MRI abdomen/pelvis report
• CT/MRI chest
• other radiology reports
• tumour measurement worksheet
• ECG report</td></tr>
<tr><td>Treatment Report (Arm 2 only)</td><td>Every 4 weeks (28 days) while patient is on protocol treatment</td><td>Within 2 weeks of the end of each 4 week reporting period</td><td>If available/applicable:
• CT/MRI abdomen/pelvis report
• CT/MRI chest
• other radiology reports
• tumour measurement worksheet
• ECG report</td></tr>
</table>

table continues on next page ...

Electronic Folder	Timing	To be completed electronically	Supporting Documentation Required[1]
End of Treatment Report(*Arm 2 only*)	As soon as permanent off treatment status is confirmed.	Within 2 weeks of end of treatment	
4-Week Post-Treatment Follow-Up Report (*Arm 2 only*)	4 weeks from the date of last infusion	Within 2 weeks of the 4-week post treatment follow up visit	• relevant radiology reports
Follow-up Report[3] (*Arm 2 only*)	Every 4 weeks (28 days) until objective progression	Within 2 weeks of follow-up visit	*If available/applicable:*<br>• CT/MRI abdomen/pelvis report<br>• tumour measurement worksheet<br>• CT/MRI chest report<br>• other radiology reports<br>• ECG report
Short Follow-up Report	Every 12 weeks after objective disease progression	Within 2 weeks of follow-up visit	
Relapse/Progression Report	Upon the patient's objective disease progression / relapse	Within 4 weeks of confirmation	• relevant radiology, operative and pathology reports
Death Report	Upon patient's death	Within 4 weeks of patient death	• autopsy/post-mortem report, if performed
Serious Adverse Event (SAE) Report[4]	Within 24 hours of event<br><br>At time of event and reported to CCTG	Within 1 working day[4]	
Minimal Follow-up Report[5]	Annual	Within 6 weeks of contact	*If available/applicable:*<br>• autopsy report<br>• CT/MRI report

1 Please scan and upload all required source documentation into EDC. Please ensure the patient's identifiers (e.g. name) are blacked-out on all source documentation.

2 It is acceptable to submit only the signature page(s) of the main consent and only the check box page(s)/signature page(s) of the optional consent provided that the version date of the consent form is indicated.

3 The aim of this folder: To collect follow-up information on all patients who have permanently discontinued Durvalumab and Tremelimumab.

4 See Section 9.0 Serious Adverse Event Reporting for details.

5 For ineligible patients who have received no protocol therapy (see Section 5.3 for details).

APPENDIX V - NCI COMMON TERMINOLOGY CRITERIA FOR ADVERSE EVENTS

The descriptions and grading scales found in the NCI Common Terminology Criteria for Adverse Events (CTCAE) version 4.0 will be utilized for Adverse Event (AE) reporting. All appropriate treatment areas should have access to a copy of the CTCAE version 4.0. A copy of the CTCAE version 4.0 can be downloaded from the CTEP website:
http://ctep.cancer.gov/protocolDevelopment/electronic_applications/ctc.htm.

APPENDIX VI - QUALITY OF LIFE ASSESSMENT

Introduction

The assumption that control of symptoms will automatically improve quality of life is probably true but hasn't yet been tested, especially in determining how certain symptoms may or may not affect quality of life. Current literature reveals interesting things; two in particular are:

- additional and useful information may be obtained from quality of life measurements
- a growing consensus that the goal of medical care today for most patients is the preservation of function and well-being in everyday life.

We have reached the stage where the collection of information about psychological distress, social disruption, emotional trauma and painful side-effects is not only necessary but a routine component in many protocols.

Quality of life data can be used in a variety of ways:

- to try to achieve the best possible outcome for patients
- to evaluate the extent of change in the quality of life of an individual or group across time
- to evaluate new treatments and technologies
- to support approval of new drug applications
- to try to provide the best value for health care dollars
- to compare costs and benefits of various financial and organizational aspects of health care services

In the future, approval of not only drugs but also new therapies or methods of delivery will most likely be based on a combination of quality of life, survival, response, and adverse event data.

Instructions for Administration of a Quality of Life Questionnaire. The instructions below are intended as a guide for the administration of the Quality of Life questionnaire.

1. Preamble

   Quality of life data are collected for research purposes, and will usually not be used for the patient's individual medical care. The assessment is in the form of a self report questionnaire. Therefore, it must be completed by the patient only, without translation, coaching or suggestions as to the "correct" answer by relatives or health care personnel.

   The usual scheduled times to obtain the questionnaires are as follows:

   - pre-randomization or pre-registration (baseline)
   - during treatment
   - during follow-up

   The information provided by the patient in the completed questionnaire is confidential and should not be discussed with or shown to anyone who is NOT mentioned in the consent form signed by the patient.

If a particular question has not been answered, please document the reason(s) in the appropriate space on the questionnaire. If the whole questionnaire has not been completed, please document the reason(s) on the appropriate case report forms.

2. Pretreatment Assessment

It should be explained to the patient that the purpose of the questionnaire is to assess the impact of treatment on different areas of the patient's life, e.g.: psychological distress, social disruption, side-effects, et cetera.

The CRA should collect the questionnaire as soon as it has been completed, check to see that each question has been answered and gently remind the patient to answer any inadvertently omitted questions. If a patient states that s/he prefers not to answer some questions and gives a reason(s), the reason(s) should be noted on the questionnaire. If a specific reason is not given, this also should be noted on the questionnaire.

3. Assessments During Treatment

The quality of life questionnaire should be given to the patient before being seen by the doctor, and prior to treatment on the day of treatment, as required by the schedule in the protocol. If the patient does not have a doctor visit scheduled, or if it was not possible for the patient to complete the questionnaire before being seen by the doctor, s/he should still complete the questionnaire prior to treatment.

4. Assessments During Follow-up

The quality of life questionnaire should be given to the patient before being seen by the doctor, on follow-up visits as required by the schedule.

A patient may, on occasion, be reluctant to complete the questionnaire because they feel unwell. In that case, you may express sympathy that things are below par, but state that this is exactly the information we require if we are to understand more about how quality of life is affected. You may also remind them that it takes only a few minutes to complete.

It defeats the whole purpose of the assessment if it is delayed until the patient feels better!

5. What If . . .

The patient should complete the questionnaires at the clinic. The exception is that the design of some trials may require the patient to take the questionnaire home with them after leaving the clinic, and complete it on the specific day, because a return visit to the clinic is not scheduled.

There may be circumstances when the patient does not complete the questionnaire as required in the clinic. Three situations are described below. In these cases, it is beneficial if quality of life data can still be collected.

A. The patient leaves the clinic before the questionnaire could be administered, or someone forgets to give the questionnaire to the patient.

Contact the patient by phone informing him or her that the questionnaire was not completed. Ask the patient if s/he is willing to complete one:

If yes, mail a blank questionnaire to the patient, and make arrangements for return of the questionnaire in a timely fashion. Record the date it was mailed and the date received on the questionnaire.

If this is not feasible, then ask the patient if s/he is willing to complete a questionnaire over the phone. If the patient agrees, read out the questions and range of possibilities, and record the answers. Make a note on the questionnaire that the questionnaire was completed over the phone.

If no, note the reason why the questionnaire was not completed on the appropriate case report form.

B. The patient goes on an extended vacation for several months and won't attend the clinic for regular visit(s).

Ensure that the patient has a supply of questionnaires, with instructions about when to complete them, and how to return them. If it is known beforehand, give the patient blank questionnaires at the last clinic visit; if the extended absence is not known in advance, mail the blank questionnaires to the patient. Written instructions may help ensure that the patient stays on schedule as much as possible.

C. The patient does not want to complete the questionnaire in clinic.

Should the patient not wish to answer the questionnaire in the clinic but insists on taking it home, and failing to comply with the patient's wishes is likely to result in the questionnaire not being completed at all, then the patient may take the questionnaire home with instructions that it is to be completed the same day. When the questionnaire is returned, the date on which the questionnaire was completed should be noted and a comment made on the questionnaire as to why the patient took it away from the clinic before completion.

6. Waiving the Quality of Life Component

The only time that we will not require a patient to complete the quality of life questionnaires is if s/he cannot comprehend either English or French (or other languages that the questionnaire may be available in). In other words, if the assistance of a translator is required to comprehend the questions and reply, the questionnaires should not be completed. Translation of the questions is not acceptable. Please indicate on questionnaire.

7. Unwillingness to Complete Quality of Life Questionnaire

If a patient speaks and reads English or French (or other languages that the questionnaires may be available in), but does not wish to complete the questionnaires then s/he is NOT eligible and should NOT be put on study.

8. Inability to Complete Quality of Life Questionnaire (for reason other than illiteracy in English or French)

An eligible patient may be willing but physically unable to complete the questionnaires, because of blindness, paralysis, etc. If the patient is completing the QOL assessment in the clinic, the questionnaire should be read to them and the answers recorded by a health care professional (e.g. preferably the clinical research associate assigned to the trial, but another clinic nurse, a doctor or social worker who is familiar with the instructions for administering the questionnaires would be acceptable). If the patient is completing the questionnaire at home, and a telephone interview by the clinical research associate is not possible, then a spouse or friend may read the questions to the patient and record the answers. However, this method should be a last resort, and the spouse or friend should be instructed to not coach or suggest answers to the patient. Whichever method is used, it should be recorded on the questionnaire.

If these special arrangements are not possible or feasible, then the patient would not be required to complete the questionnaires, and this should be reported on the appropriate case report form.

Quality of Life Questionnaire – ENGLISH

CCTG Trial: **CO.26**

This **page** to be completed by the Clinical Research Associate

Patient Information

CCTG Patient Serial No: ___________ Patient Initials: ____ ____ ____ (first-middle-last)

Institution: ____ Investigator: ________________________________

Scheduled time to obtain quality of life assessment: please check (✓)

☐ Prior to randomization

After randomization:
☐ 4 weeks ☐ 8 weeks ☐ 12 weeks ☐ 16 weeks ☐ 24 weeks ☐ _____ weeks

Were ALL questions answered? ___ Yes ___ No If no, reason: ____

Was assistance required? ___ Yes ___ No If yes, reason: ____

Where was questionnaire completed: ☐ home ☐ clinic ☐ another centre

Comments: ____

____

Date Completed: __ __ __ __ - __ __ __ - __ __
yyyy mmm dd

PLEASE ENSURE THIS PAGE IS FOLDED BACK BEFORE HANDING TO THE PATIENT FOR QUESTIONNAIRE COMPLETION.

CCTG use only

Logged: _______ Study Coord: _______
______ - ____ - ____ ______ - ____ - ____

This box to be completed by the clinical research associate: Pt. Serial #: ______________ Pt. Initials: ____ ____ ____

European Organization for Research and Treatment of Cancer (EORTC)

Quality of Life Questionnaire (CO.26)

We are interested in some things about you and your health. Please answer all the questions **yourself** by circling the number that best applies to you. There are no 'right' or 'wrong' answers. Choose the best **single** response that applies to you. The information that you provide is for research purposes and will remain strictly confidential. The individuals (e.g. doctors, nurses, etc.) directly involved in your care will not usually see your responses to these questions -- if you wish them to know this information, please bring it to their attention.

	Not At All	A Little	Quite a Bit	Very Much
1. Do you have any trouble doing strenuous activities, like carrying a heavy shopping bag or a suitcase?	1	2	3	4
2. Do you have any trouble taking a long walk?	1	2	3	4
3. Do you have any trouble taking a short walk outside of the house?	1	2	3	4
4. Do you need to stay in a bed or a chair during the day?	1	2	3	4
5. Do you need help with eating, dressing, washing yourself or using the toilet?	1	2	3	4

During the past week:	Not At All	A Little	Quite a Bit	Very Much
6. Were you limited in doing either your work or other daily activities?	1	2	3	4
7. Were you limited in pursuing your hobbies or other leisure time activities?	1	2	3	4
8. Were you short of breath?	1	2	3	4

Please go on to the next page

EORTC QLQ-C30 (Core 30 Questionnaire Version 3.0 © copyright 1995 EORTC Quality of Life Study Group. All rights reserved.)

This box to be completed by the clinical research associate: Pt. Serial #: ______________ Pt. Initials: ____ ____ ____

During the past week:	Not At All	A Little	Quite a Bit	Very Much
9. Have you had pain?	1	2	3	4
10. Did you need to rest?	1	2	3	4
11. Have you had trouble sleeping?	1	2	3	4
12. Have you felt weak?	1	2	3	4
13. Have you lacked appetite?	1	2	3	4
14. Have you felt nauseated?	1	2	3	4
15. Have you vomited?	1	2	3	4
16. Have you been constipated?	1	2	3	4
17. Have you had diarrhea?	1	2	3	4
18. Were you tired?	1	2	3	4
19. Did pain interfere with your daily activities?	1	2	3	4
20. Have you had difficulty in concentrating on things, like reading a newspaper or watching television?	1	2	3	4
21. Did you feel tense?	1	2	3	4
22. Did you worry?	1	2	3	4

Please go on to the next page

This box to be completed by the clinical research associate: Pt. Serial #: ____________ Pt. Initials: ____ ____ ____

During the past week:	**Not At All**	**A Little**	**Quite a Bit**	**Very Much**
23. Did you feel irritable?	1	2	3	4
24. Did you feel depressed?	1	2	3	4
25. Have you had difficulty remembering things?	1	2	3	4
26. Has your physical condition or medical treatment interfered with your family life?	1	2	3	4
27. Has your physical condition or medical treatment interfered with your social activities?	1	2	3	4
28. Has your physical condition or medical treatment caused you financial difficulties?	1	2	3	4

For the following questions please circle the number between 1 and 7 that best applies to you.

29. How would you rate your overall health during the past week?

1	2	3	4	5	6	7
Very Poor						Excellent

30. How would you rate your overall quality of life during the past week?

1	2	3	4	5	6	7
Very Poor						Excellent

Please check to make sure you have answered all the questions.

Please fill in your initials to indicate that you have completed this questionnaire: ______________

Today's date (Year, Month, Day): ________________________________

Thank you.

LIST OF CONTACTS

	Contact	Tel. #	Fax #
ELIGIBILITY CHECKLIST<br>Must be completed prior to allocation.	Julia Baran and Vicki Classen<br>Clinical Trials Assistants, CCTG<br>Email:<br>jbaran@ctg.queensu.ca or<br>vclassen@ctg.queensu.ca	613-533-6430	613-533-2941
STUDY SUPPLIES<br>Forms, Protocols	Available on CCTG Website:<br>http://www.ctg.queensu.ca<br>under: *Clinical Trials*		
PRIMARY CONTACTS FOR GENERAL PROTOCOL-RELATED QUERIES<br>(including eligibility questions and protocol management)	Nadine Magoski<br>Study Coordinator, CCTG<br>Email: nmagoski@ctg.queensu.ca<br>or:<br>Dr. Chris O'Callaghan<br>Senior Investigator, CCTG<br>Email: cocallaghan@ctg.queensu.ca	613-533-6430	613-533-2941
STUDY CHAIR	Dr. Eric Chen<br>Study Chair<br>Email: eric.chen@uhn.on.ca	416-946-2263	416-946-4467
SERIOUS ADVERSE EVENT REPORTING<br>See protocol Section 9.0 for details of reportable events.	Dr. Chris O'Callaghan<br>Senior Investigator, CCTG<br>or:<br>Nadine Magoski<br>Study Coordinator, CCTG	613-533-6430	613-533-2941
DRUG ORDERING<br>See Appendix II and the CO.26 pharmacy information manual for full details.	See Appendix III and trial website:<br>http://www.ctg.queensu.ca/trials/gi/CO26/CO26.html<br>for details and contact information		
ELECTRONIC DATA CAPTURE (EDC) AND RIPPLE<br>(technical support)	CCTG Home Page (Toolbox):<br>https://scooby.ctg.queensu.ca<br>Email Support Staff at: support@ctg.queensu.ca		
