# Supplementary material for: Liver Metastases and Immune Checkpoint Inhibitor Efficacy in Patients With Refractory Metastatic Colorectal Cancer: A Secondary Analysis of a Randomized Clinical Trial
Source: JAMA Netw Open. 2023 Dec 5;6(12):e2346094. doi: 10.1001/jamanetworkopen.2023.46094 (PMC10698621; doi:10.1001/jamanetworkopen.2023.46094)
Supplement: Supplement 2. — eFigure. Comparisons of pTMB and Common Gene Alterations Between Patients With and Without Liver Metastases [file jamanetwopen-e2346094-s002.pdf]

# Supplementary Online Content

Chen EX, Loree JM, Titmiss E, et al. Liver metastases and immune checkpoint inhibitor efficacy in refractory metastatic colorectal cancer: a secondary analysis of a randomized clinical trial. *JAMA Netw Open*. 2023;6(12):e2346094.
doi:10.1001/jamanetworkopen.2023.46094

**eFigure.** Comparisons of pTMB and Common Gene Alterations Between Patients With and Without Liver Metastases

This supplementary material has been provided by the authors to give readers additional information about their work.

© 2023 Chen EX et al. *JAMA Network Open*.

**eFigure.** Comparisons of pTMB and Common Gene Alterations Between Patients With and Without Liver Metastases

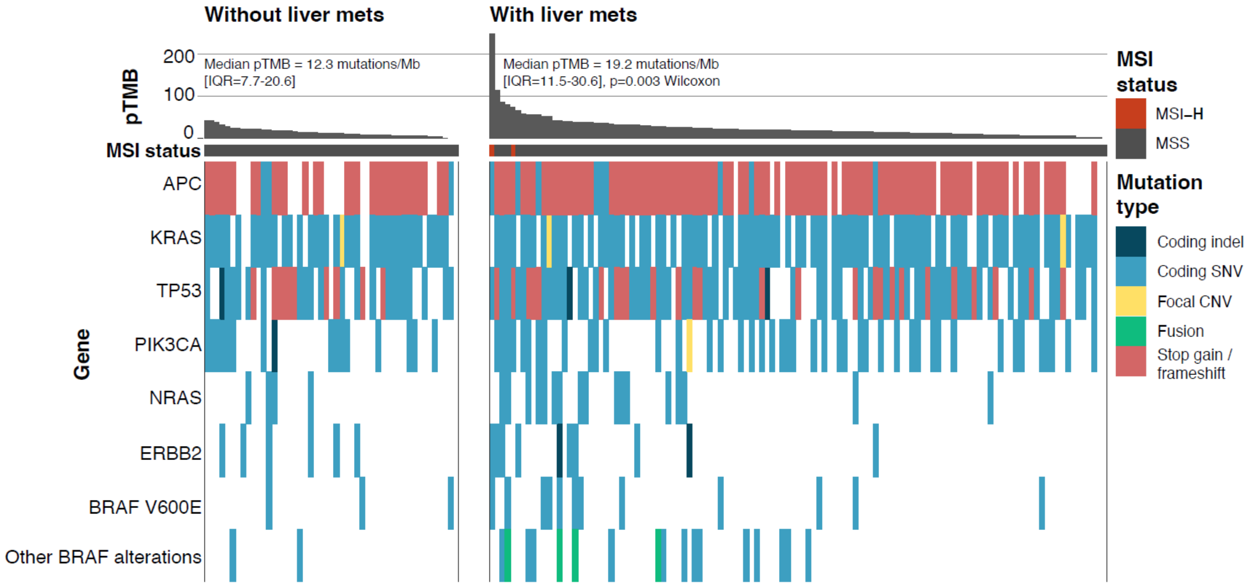


© 2023 Chen EX et al. *JAMA Network Open*.
