# Supplementary material for: Liver Metastases and Immune Checkpoint Inhibitor Efficacy in Patients With Refractory Metastatic Colorectal Cancer: A Secondary Analysis of a Randomized Clinical Trial
Source: JAMA Netw Open. 2023 Dec 5;6(12):e2346094. doi: 10.1001/jamanetworkopen.2023.46094 (PMC10698621; doi:10.1001/jamanetworkopen.2023.46094)
Supplement: Supplement 3. — Data Sharing Statement [file jamanetwopen-e2346094-s003.pdf]

Data Sharing Statement

Chen. Liver Metastases and Immune Checkpoint Inhibitor Efficacy in Patients With Refractory Metastatic Colorectal Cancer. *JAMA Netw Open*. Published December 05, 2023.
doi:10.1001/jamanetworkopen.2023.46094

Data
Data available: Yes
Data types: Deidentified participant data
How to access data: https://www.ctg.queensu.ca/
When available: With publication

Supporting Documents
Document types: None

Additional Information
Who can access the data: researchers whose proposal has been approved.
Types of analyses: specific purpose, with proposal
Mechanisms of data availability: after approval of proposal
